# Supplementary material for: Metagenomics survey unravels diversity of biogas microbiomes with potential to enhance productivity in Kenya
Source: PLoS One. 2021 Jan 4;16(1):e0244755. doi: 10.1371/journal.pone.0244755 (PMC7781671; doi:10.1371/journal.pone.0244755)
Supplement: S1 Table — (PDF) [file pone.0244755.s001.pdf]

S1_Table. The quality control statistics of the utilized scaffolds of the twelve treatments

	Statistics of the filtered scaffolds for downstream analysis					Statistics of scaffolds GC content and ambiquity				
**Sample id**	**Base pair**	**Sequence**	**Length (bp)**	**Av. Length Bp**	**Av. Std**	**GC%**	**Std dev.**	**GC-ratio**	**Std dev.**	**Amb. Reads**
S_1	89,283332	313,836	56-136,866	284	348.635	52.385	11.093	1.009	0.492	2,600
S_2	107,701,975	394,463	56-64,851	273	189.197	55.033	11.236	0.908	0.463	1,5580
S_3	90,908,777	310,336	56-55621	292	276.697	52.446	10.810	1.00	0.471	2,800
S_4	83,626,041	275,624	55-34,847	303	372.306	49.967	10.900	1.108	0.514	1,220
S_5	74,806,357	280,930	55-5,606	266	85.933	49.220	11.339	1.154	0.560	470
S_6	75,808,027	253,949	56-55,621	298	355.114	53.626	10.547	0.948	0.441	10,026
S_7	133,718,872	428,821	56-95,185	311	510.825	49.603	11.172	1.133	0.549	5,020
S_8	63,285,799	212,099	56-56,718	298	292.399	50.066	10.639	1.100	0.507	1,620
S_9	188,414,920	621,662	56-44,371	303	305.181	49.315	11.075	1.145	0.548	7,284
S_10	56,387,075	217,027	56-64,831	259	171.992	55.568	11.027	0.884	0.439	340
S_11	59,129,493	198,327	56-216,425	298	996.110	48.738	10.866	1.167	0.546	2,491
S_12	96,394,918	312,853	56-238,965	308	1024.420	50.749	10.631	1.065	0.468	3,865

*S_*1: *Biogas Biome* 1; *S_2*: *Biogas Biome* 2.0; *S_3*: *Biogas Biome* 3.10; *S_4*: *Biogas Biome* 4.10; *S_5*: *Biogas Biome* 5.10; *S_6*: *Biogas Biome* 6.10; *S_7*: *Biogas Biome* 7.10; *S_8*: *Biogas Biome* 8.10; *S_*9: *Biogas Biome* 9.10; *S_10*: *Biogas Biome* 10.10; *S_11*: *Biogas Biome* 11.10. *S_*12: *Biogas Biome* 12.10
