# Supplementary material for: Metagenomics survey unravels diversity of biogas microbiomes with potential to enhance productivity in Kenya
Source: PLoS One. 2021 Jan 4;16(1):e0244755. doi: 10.1371/journal.pone.0244755 (PMC7781671; doi:10.1371/journal.pone.0244755)
Supplement: S1 Fig — Barchart showing unfiltered and filtered sequencing reads (a) and the known and unknown protein genes (b) in our samples. More than 53.07% of the filtered nucleotide reads in our samples contained unknown proteins. (PDF) [file pone.0244755.s002.pdf]

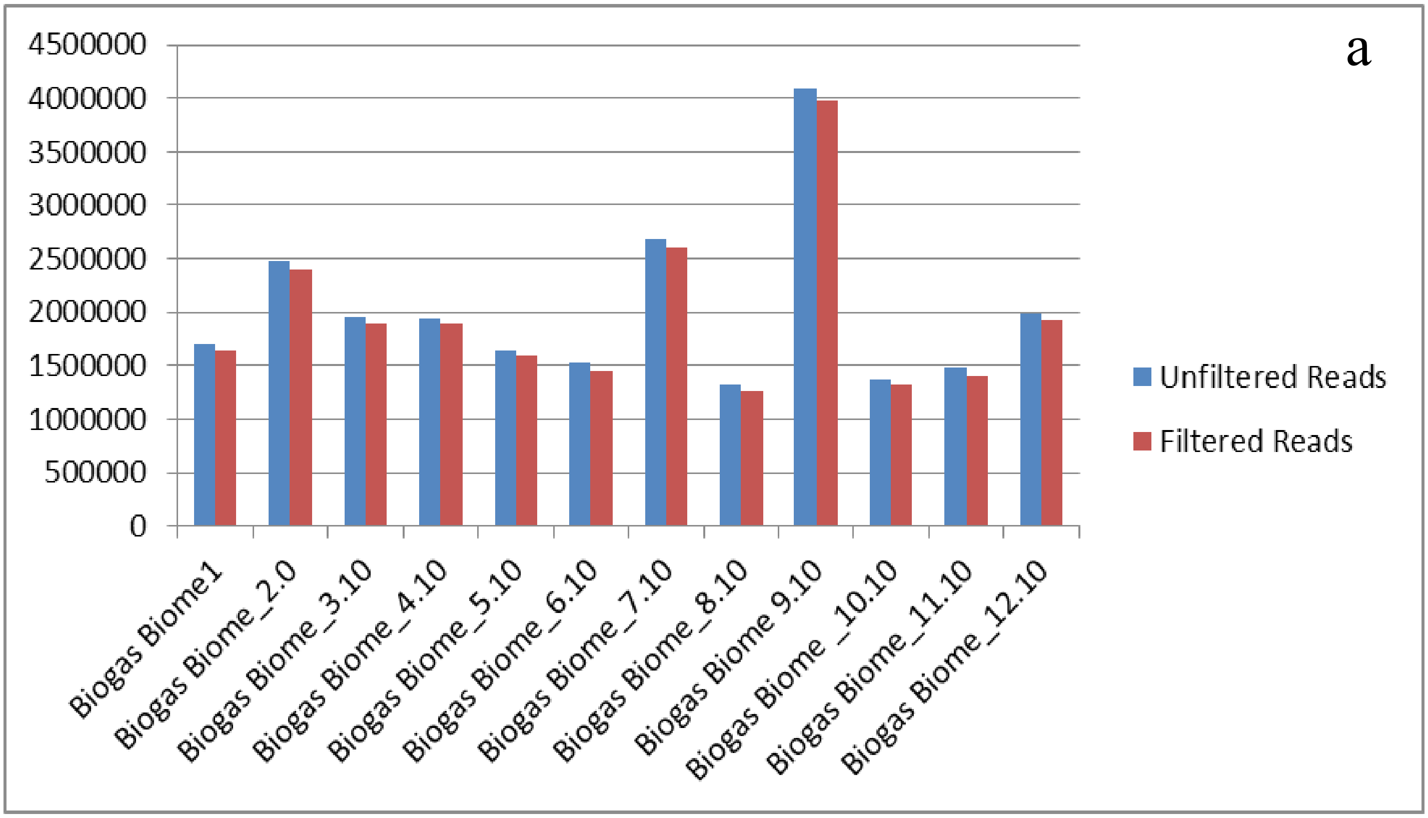

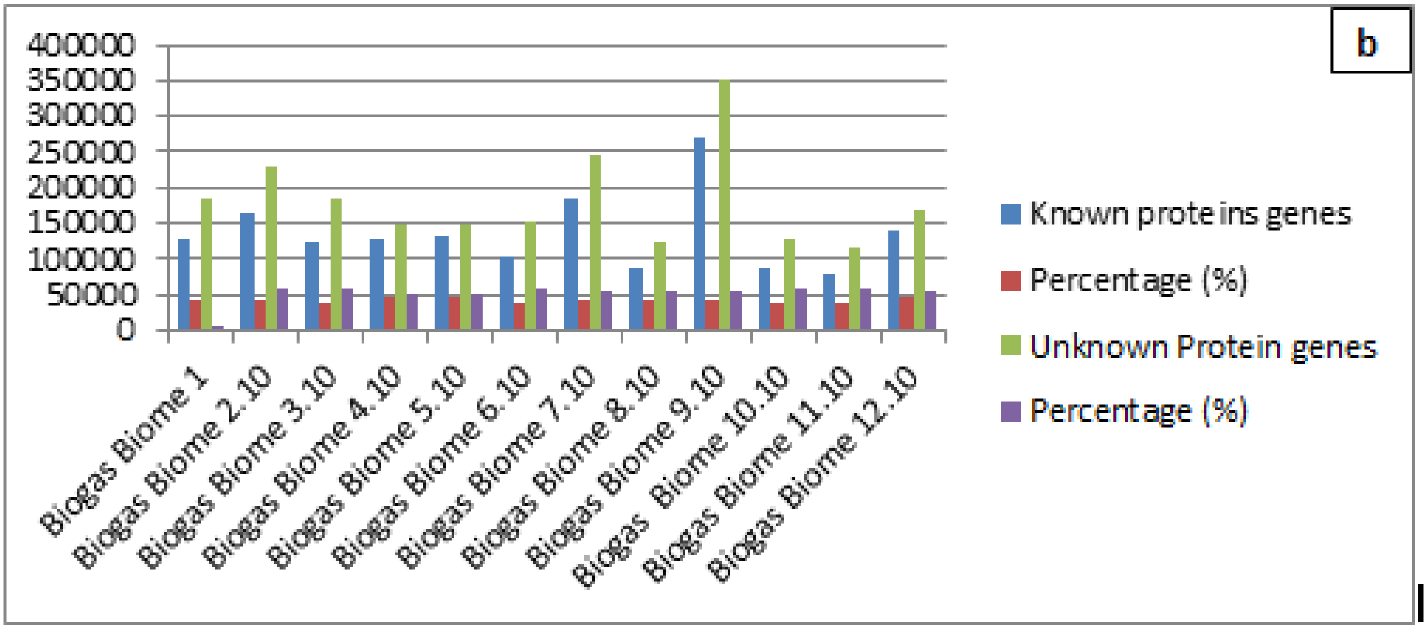

S1 Fig . Bar charts showing a). Unfiltered and filtered sequencing reads and b). The known and unknown protein genes in our samples. More than 53.07% of the filtered nucleotide reads in our samples contained unknown proteins.
