# Supplementary material for: Metagenomics survey unravels diversity of biogas microbiomes with potential to enhance productivity in Kenya
Source: PLoS One. 2021 Jan 4;16(1):e0244755. doi: 10.1371/journal.pone.0244755 (PMC7781671; doi:10.1371/journal.pone.0244755)
Supplement: S2 Fig — Stacked barchat showing some (21 phyla) of the identified Bacteria domain phyla, relative abundances (a) and the PCoA plot (phylum level), based on the Euclidean model (b). The communities’ nucleotide composition of reactor 2 and 10 and those of reactors 4 and 9 partially clustered in the upper left and right quadrants of the plot respectively. (PDF) [file pone.0244755.s003.pdf]

a

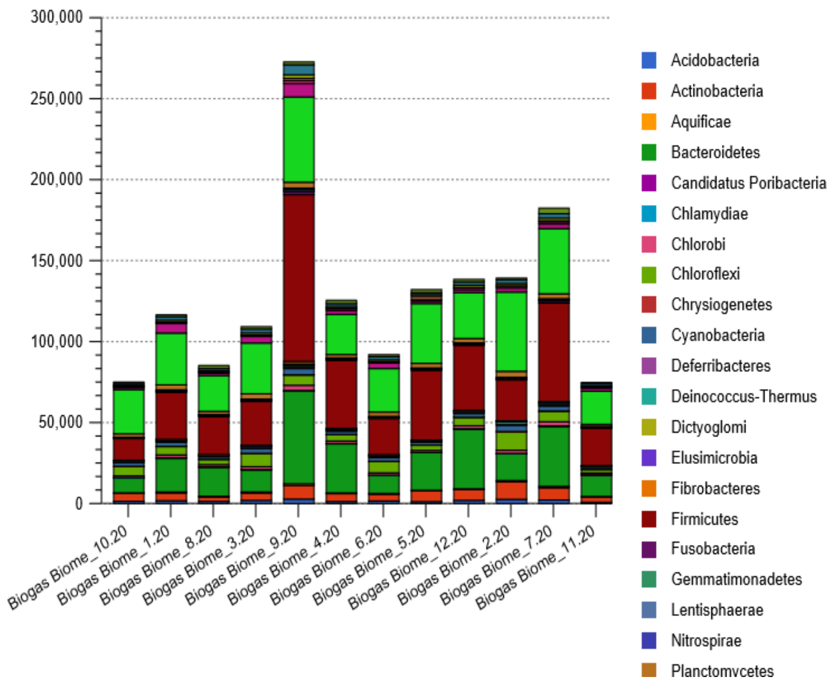

b

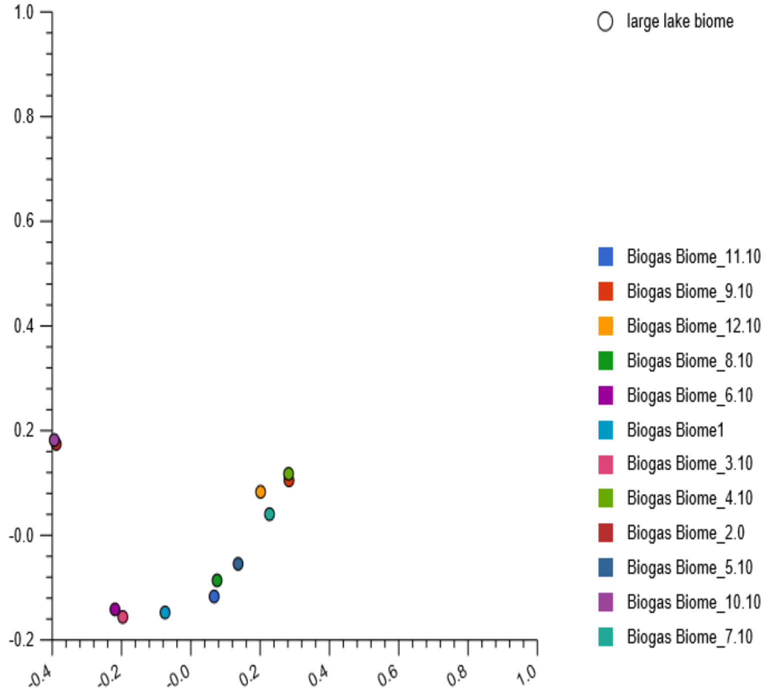

S2 Fig. Stacked barchat (a) showing some (21 phyla) of the identified bacteria domain phyla, relative abundances and the PCoA plot (phylum level) (b), based on the Euclidean model. The communities' nucleotide composition of reactor 2 and 10 and those of reactors 4 and 9 partially clustered in the upper left and right quadrants of the plot respectively.
