# Supplementary material for: Metagenomics survey unravels diversity of biogas microbiomes with potential to enhance productivity in Kenya
Source: PLoS One. 2021 Jan 4;16(1):e0244755. doi: 10.1371/journal.pone.0244755 (PMC7781671; doi:10.1371/journal.pone.0244755)
Supplement: S3 Fig — The PCoA plot showing the relative abundances variation at the class (a) and order level (b). At the class and order level, the nucleotide composition of reactor 2 and 10, were positioned on the lower left quadrant of the plot, those of reactor 3 and 6, on the upper left quadrant, the communities of reactor 8 and 11, on the upper right quadrant, while the composition of reactor 4 and 9, were postioned on the lower right quadrant of the plot. All clustered partially at both level. (PDF) [file pone.0244755.s004.pdf]

a

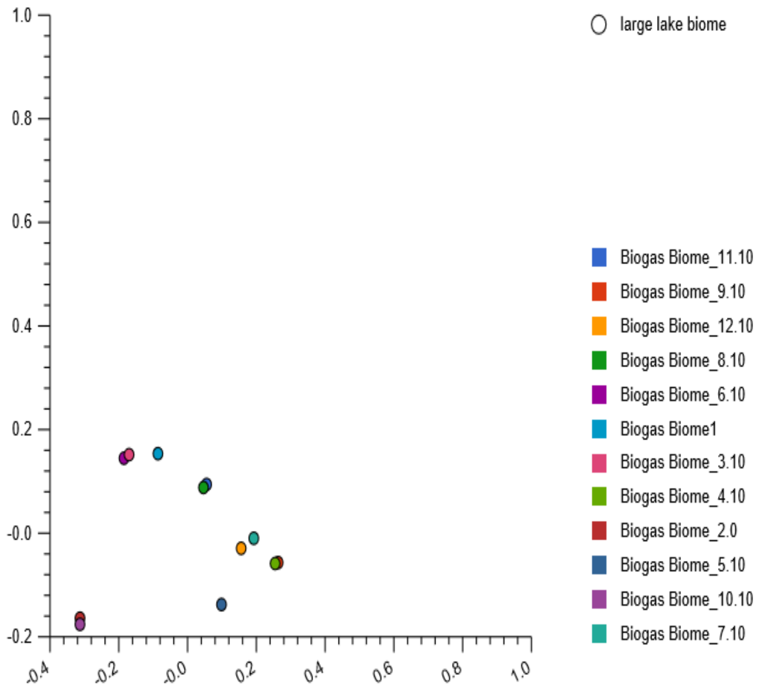

b

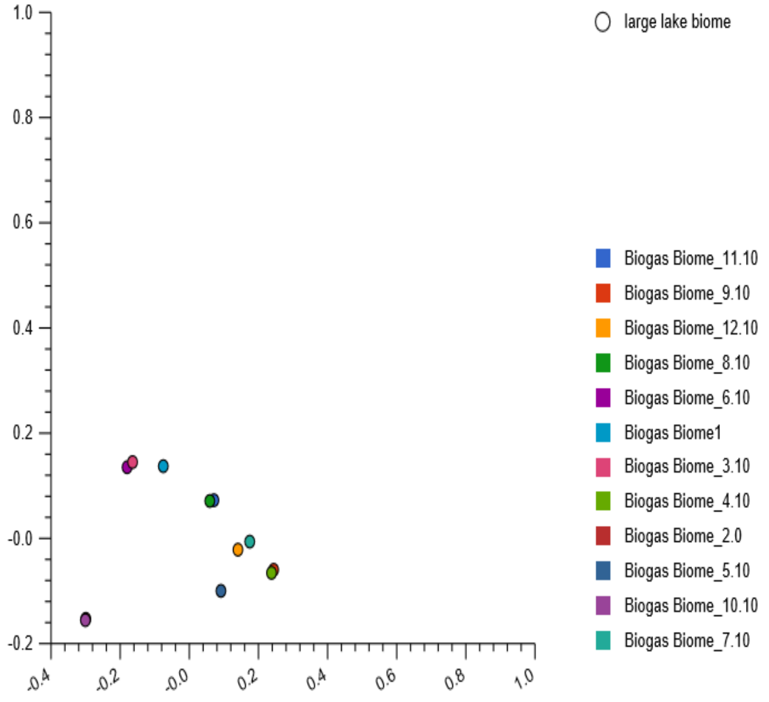

S3 Fig. The PCoA plot showing the (a) the nucleotide composition variation at the class and (b) the nucleotide composition variation at order level. At the class and order level, the nucleotide composition of reactor 2 and 10, were positioned on the lower left quadrant of the plot, those of reactor 3 and 6, on the upper left quadrant, the communities of reactor 8 and 11, on the upper right quadrant, while the composition of reactor 4 and 9, were postioned on the lower right quadrant of the plot. All clustered partially at both level.
