# Supplementary material for: Metagenomics survey unravels diversity of biogas microbiomes with potential to enhance productivity in Kenya
Source: PLoS One. 2021 Jan 4;16(1):e0244755. doi: 10.1371/journal.pone.0244755 (PMC7781671; doi:10.1371/journal.pone.0244755)
Supplement: S4 Fig — The stacked barchart revealing six classes of Proteobacteria communities, relative abundances (a) and their PCoA plots based on the Euclidean model (b). The nucleotide composition for reactor 1, 3 and 6 clustered on the upper right quadrant of the plot. Similarly, the composition of reactor 4, 7 and 12 clustered partially on the lower right, while those of reactor 5 were singly positioned on the upper left quadrant of the plot. (PDF) [file pone.0244755.s005.pdf]

a

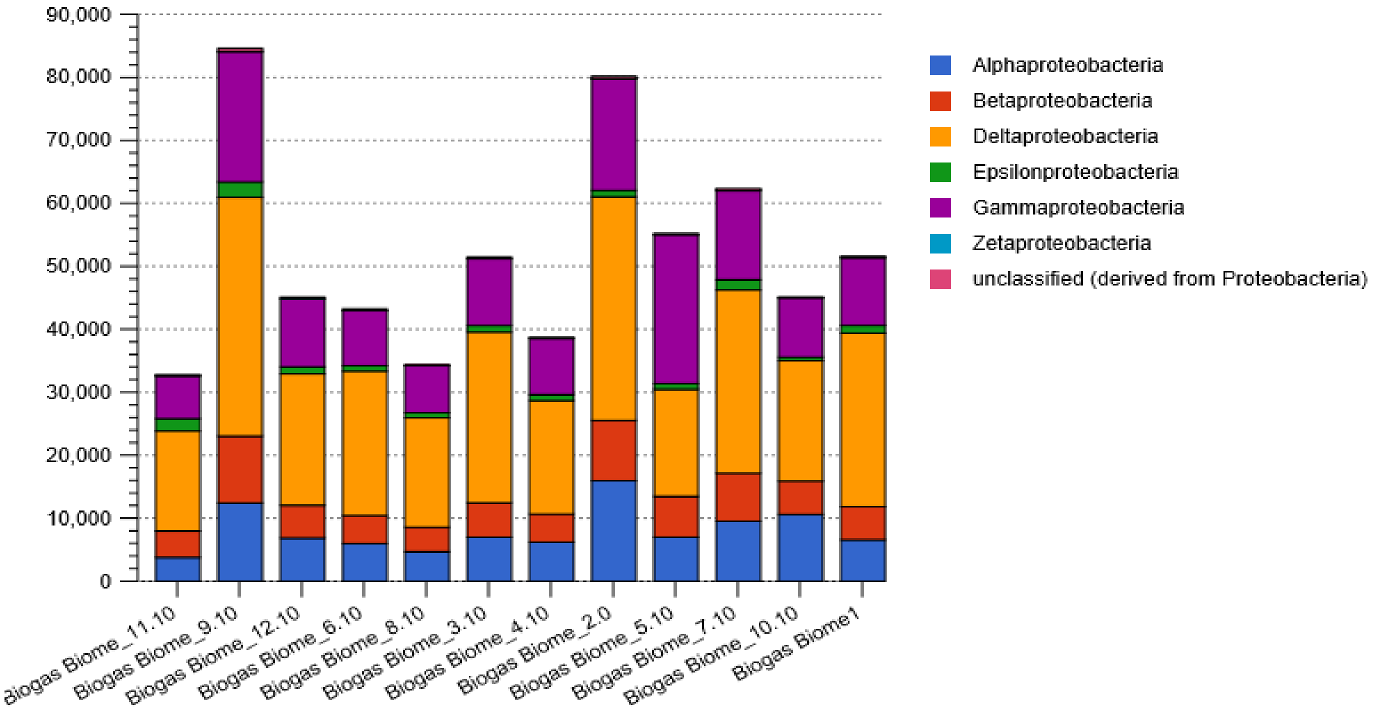

b

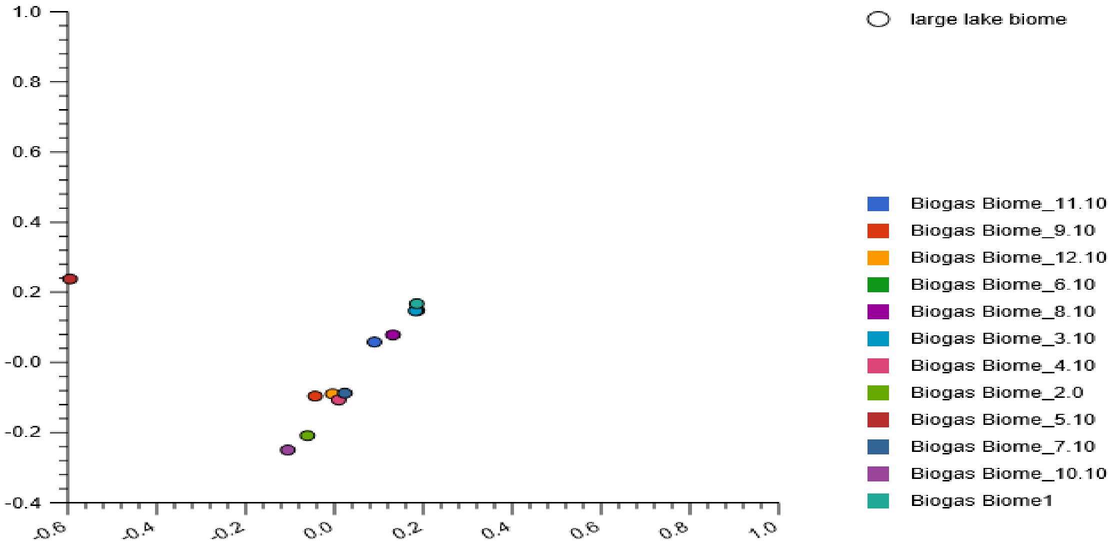

S4 Fig. The stacked barchart revealing six Proteobacteria classes, the proportions of their relative abundances (a) and their PCoA plots (b) based on the Euclidean model. The nucleotide composition for reactor 1, 3 and 6 clustered on the upper right quadrant of the plot. Similarly, the composition of reactor 4, 7 and 12 clustered partially on the lower right, while those of reactor 5 were singly positioned on the upper left quadrant of the plot.
