# Supplementary material for: Metagenomics survey unravels diversity of biogas microbiomes with potential to enhance productivity in Kenya
Source: PLoS One. 2021 Jan 4;16(1):e0244755. doi: 10.1371/journal.pone.0244755 (PMC7781671; doi:10.1371/journal.pone.0244755)
Supplement: S5 Fig — The PCoA plot indicated partial similarities of the nucleotide composition in half of the studied treatments, including reactor 1, 3 and 6, upper right quadrant; reactor 4, 7 and 12, lower right quadrant of the plot at the class level. Similar observations were made at the order level, with an exception of few treatments (reactor 4, 7, 8 and 9 that formed cluster). (PDF) [file pone.0244755.s006.pdf]

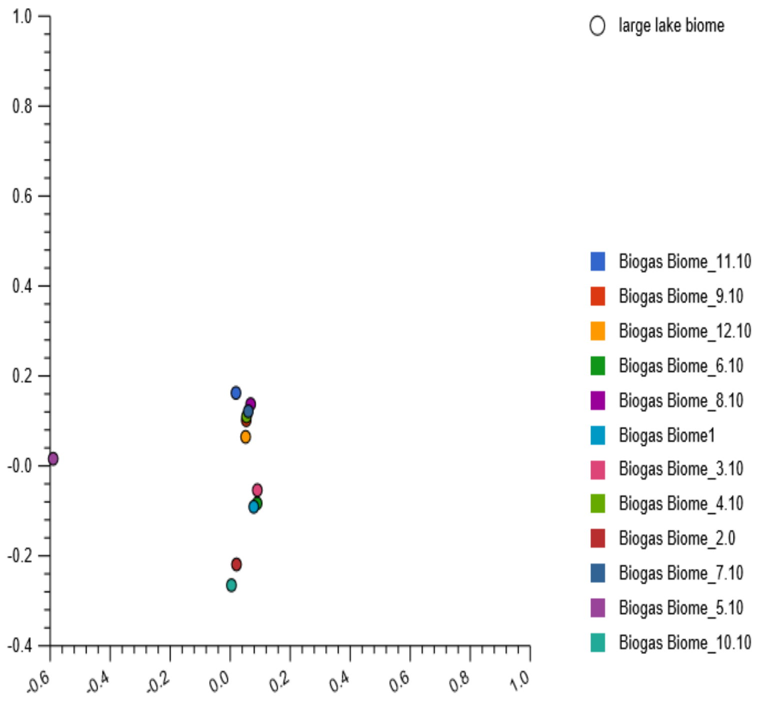

S5 Fig. The PCoA plot based on the Euclidean model for *Proteobacteria* order. The PCoA plot indicated partial similarities of the nucleotide composition in half of the studied treatments, including reactor 1, 3 and 6, upper right quadrant; reactor 4, 7 and 12, lower right quadrant of the plot at the class level. Similar observations were made at the order level, with an exception of few treatments (reactor 4, 7, 8 and 9 that formed cluster).
