# Supplementary material for: Metagenomics survey unravels diversity of biogas microbiomes with potential to enhance productivity in Kenya
Source: PLoS One. 2021 Jan 4;16(1):e0244755. doi: 10.1371/journal.pone.0244755 (PMC7781671; doi:10.1371/journal.pone.0244755)
Supplement: S6 Fig — Stacked barchat showing seven δ-Proteobacteria orders, relative abundances (a) and their PCoA plot revealing nucleotide composition variations, based on the Euclidean model (b). The plot revealed dissimilarity of nucleotide composition among the treatments, with an exception of the composition of reactor 4 and 7 that were found to cluster on the lower left quadrant of the plot. (PDF) [file pone.0244755.s007.pdf]

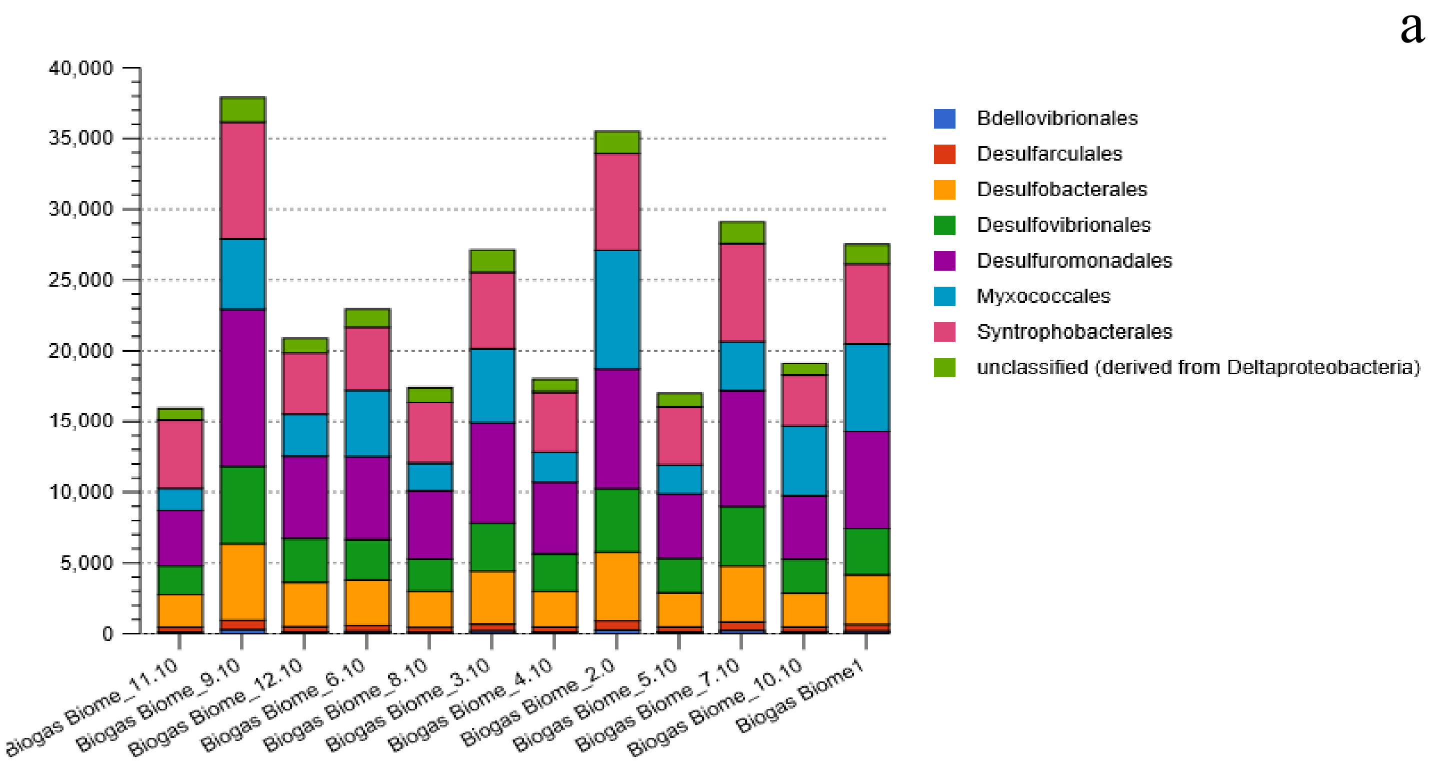


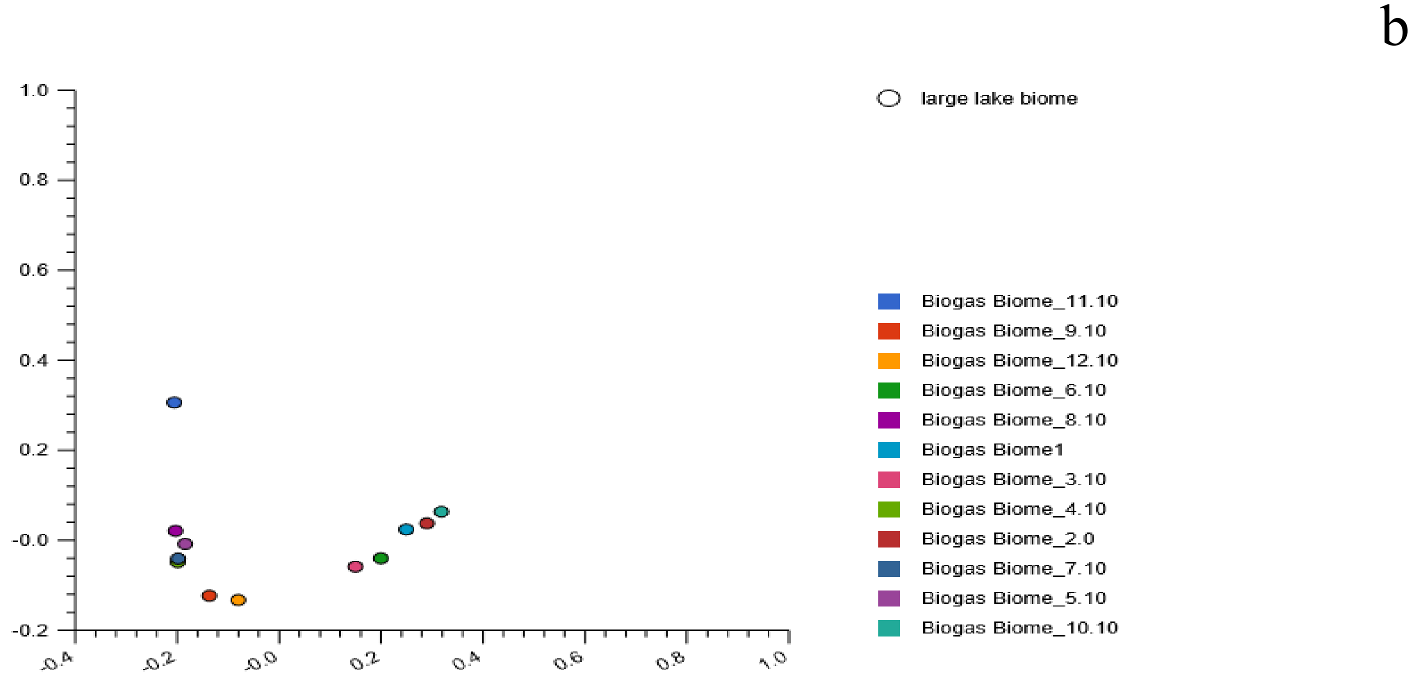


**S6 Fig. Stacked barchat showing seven δ-Proteobacteria orders, relative abundances (a) and their PCoA plot revealing nucleotide composition variations, based on the Euclidean model.** The plot revealed dissimilarity of nucleotide composition among the treatments, with an exception of the composition of reactor 4 and 7 that were found to cluster on the lower left quadrant of the plot.
