# Supplementary material for: Metagenomics survey unravels diversity of biogas microbiomes with potential to enhance productivity in Kenya
Source: PLoS One. 2021 Jan 4;16(1):e0244755. doi: 10.1371/journal.pone.0244755 (PMC7781671; doi:10.1371/journal.pone.0244755)
Supplement: S7 Fig — Stacked barchart showing fourteen γ-Proteobacteria orders (a) and their PCoA plot based on the Eucleaden model (b). The plot revealed clustering of the reactor 1 and 3 nucleotide composition, which were in close proximity with the composition of reactor 7. The communities of reactor 7 clustered partially with those of reactor 8 and 12. However, the composition of reactor 4 and 9 were in close proximity. Interestingly, all the clustered treatments were positioned on the upper right quadrant of the plot. Moreover, the nucleotide compositions of reactor 5 were singly positioned on the upper left quadrant of the plot. (PDF) [file pone.0244755.s008.pdf]

a

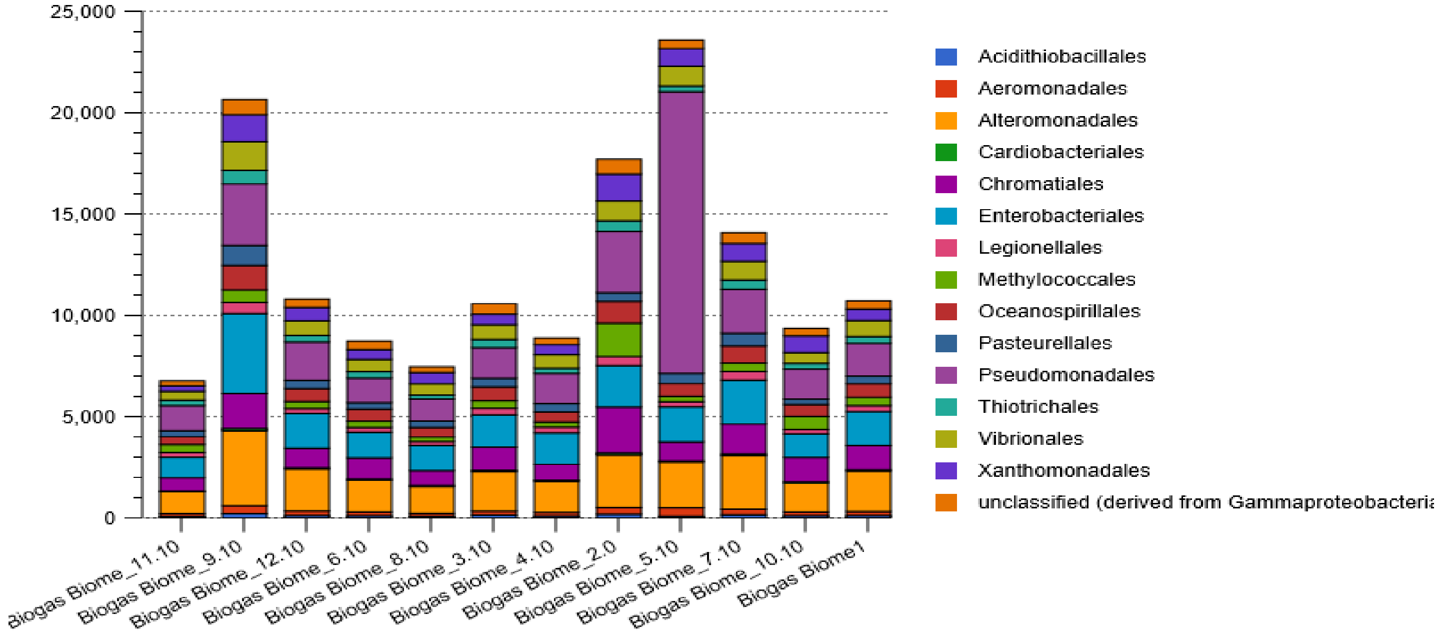

c

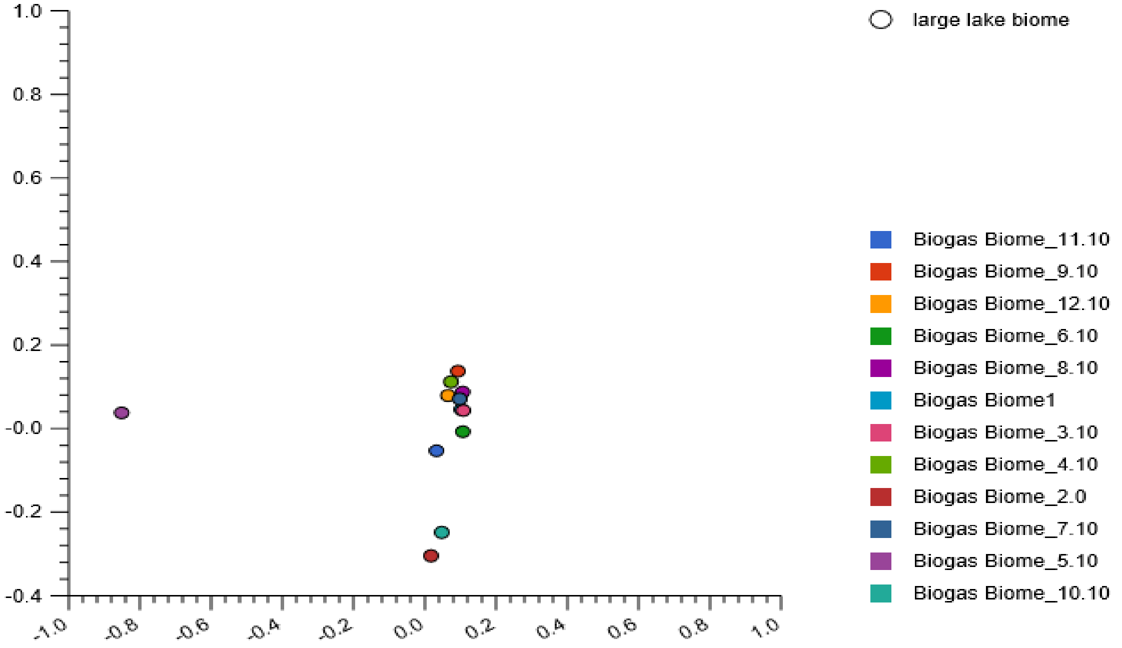

S7 Fig. Stacked barchart showing fourteen γ-Proteobacteria orders (a) and their PCoA plot based on the Eucleaden model. The plot revealed clustering of the reactor 1 and 3 nucleotide composition, which were in close proximity with the composition of reactor 7. The communities of reactor 7 clustered partially with those of reactor 8 and 12. However, the composition of reactor 4 and 9 were in close proximity. Interestingly, all the clustered treatments were positioned on the upper right quadrant of the plot. Moreover, the nucleotide compositions of reactor 5 were singly positioned on the upper left quadrant of the plot.
