# Supplementary material for: Metagenomics survey unravels diversity of biogas microbiomes with potential to enhance productivity in Kenya
Source: PLoS One. 2021 Jan 4;16(1):e0244755. doi: 10.1371/journal.pone.0244755 (PMC7781671; doi:10.1371/journal.pone.0244755)
Supplement: S8 Fig — Stacked barchat showing seven β-proteobacteria orders, relative abundances (a) and their PCoA plot revealing nucleotide composition (dis)similarities among the treatments (b). The plots revealed partial clustering of reactor 7 and 8 nucleotide compositions while the nucleotide composition of reactor 3, 6 and 12 were found to cluster on the lower left quadrant of the plot. The majority of the treatments comprised dissimilar β-Proteobacteria nucleotide compositions. (PDF) [file pone.0244755.s009.pdf]

a

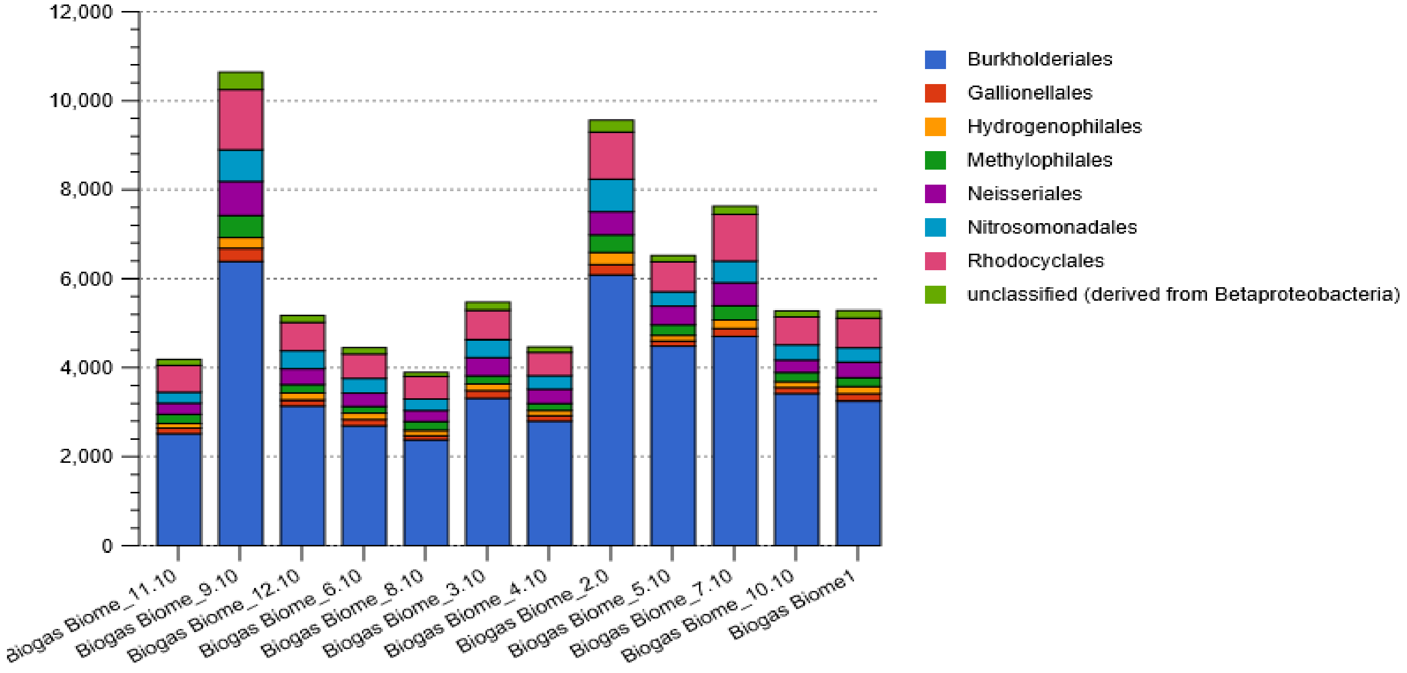

b

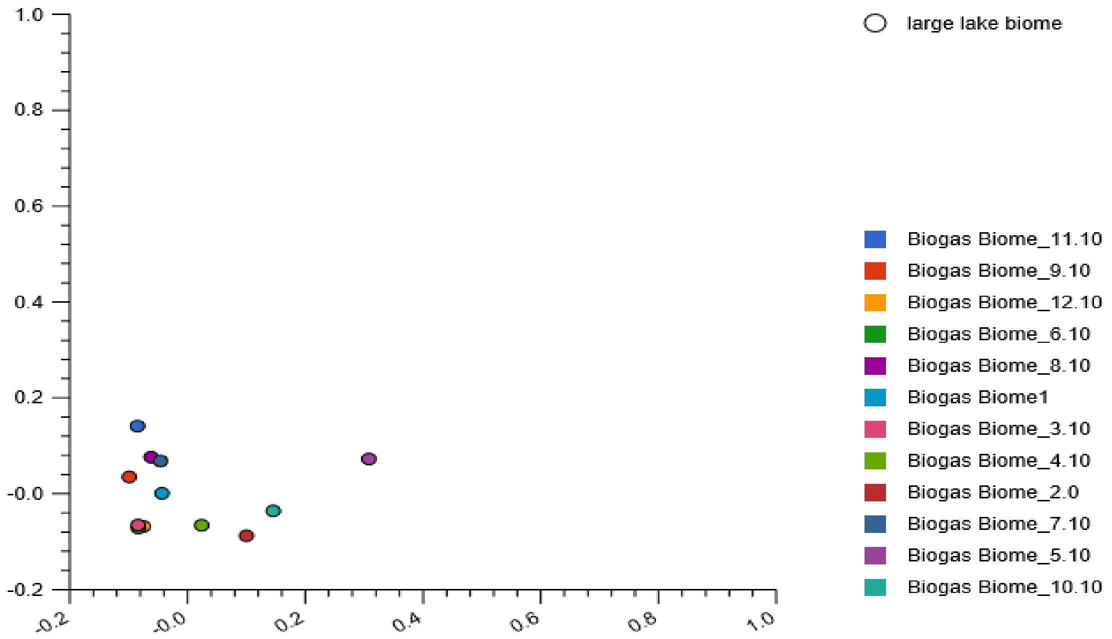

S8 Fig. Stacked barchat showing seven β-proteobacteria orders, relative abundances (a) and their PCoA plot (b) revealing nucleotide composition (dis)similarities among the treatments. The plots revealed partial clustering of reactor 7 and 8 nucleotide compositions while the nucleotide composition of reactor 3, 6 and 12 were found to cluster on the lower left quadrant of the plot. The majority of the treatments comprised dissimilar *β-Proteobacteria* nucleotide compositions.
