# Supplementary material for: Metagenomics survey unravels diversity of biogas microbiomes with potential to enhance productivity in Kenya
Source: PLoS One. 2021 Jan 4;16(1):e0244755. doi: 10.1371/journal.pone.0244755 (PMC7781671; doi:10.1371/journal.pone.0244755)
Supplement: S9 Fig — Stacked barchat showing the seven α-Proteobacteria orders, relative abundances (a) and their PCoA plots, revealing the nucleotide composition variation among the reactors based on the Euclidean model (b). The plots revealed close proximity of reactor 1 and 6 nucleotide composition and were positioned on the y-axis. However, the composition of reactor 3 and 11 clustered partially on the upper right quadrant while the nucleotide compositions of the other treatments were found to reveal dissimilarity. (PDF) [file pone.0244755.s010.pdf]

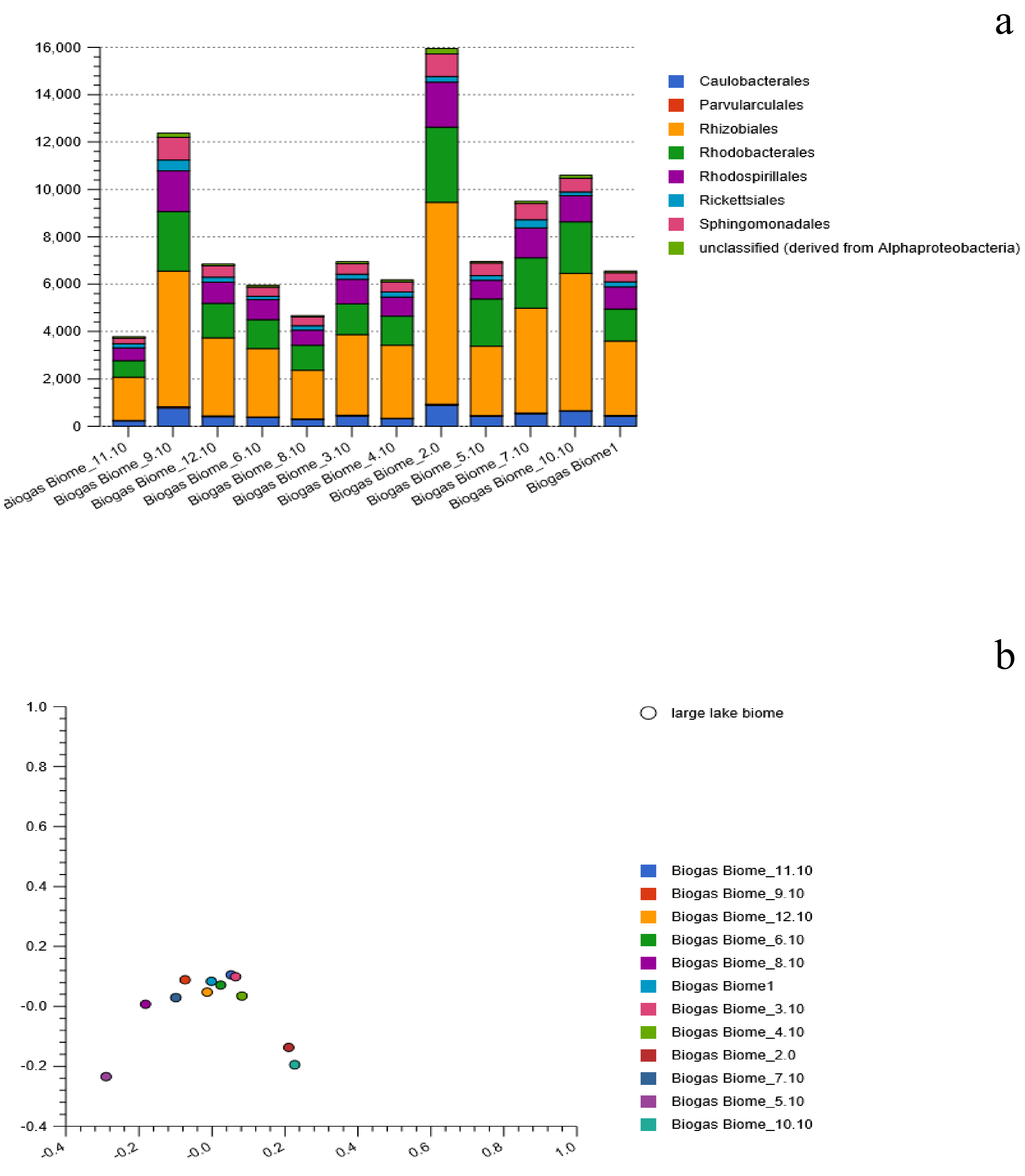

S9 Fig. Stacked barchat (a) showing the seven *α-Proteobacteria* orders, relative abundances and their PCoA plots (b), revealing the nucleotide composition variation among the reactors based on the Euclidean model. The plots revealed close proximity of reactor 1 and 6 nucleotide composition and were positioned on the y-axis. However, the composition of reactor 3 and 11 clustered partially on the upper right quadrant while the nucleotide compositions of the other treatments were found to reveal dissimilarity.
