# Supplementary material for: Metagenomics survey unravels diversity of biogas microbiomes with potential to enhance productivity in Kenya
Source: PLoS One. 2021 Jan 4;16(1):e0244755. doi: 10.1371/journal.pone.0244755 (PMC7781671; doi:10.1371/journal.pone.0244755)
Supplement: S10 Fig — Stacked barchat showing two ε-Proteobacteria orders, relative abundances (a) and their PCoA plot based on Euclidean model (b). The PCoA plot for reactor 4 and 8 clustered on the upper left quadrant of the plot while those of reactor 5 and 9 clustered partially on the upper right quadrant. (PDF) [file pone.0244755.s011.pdf]

a

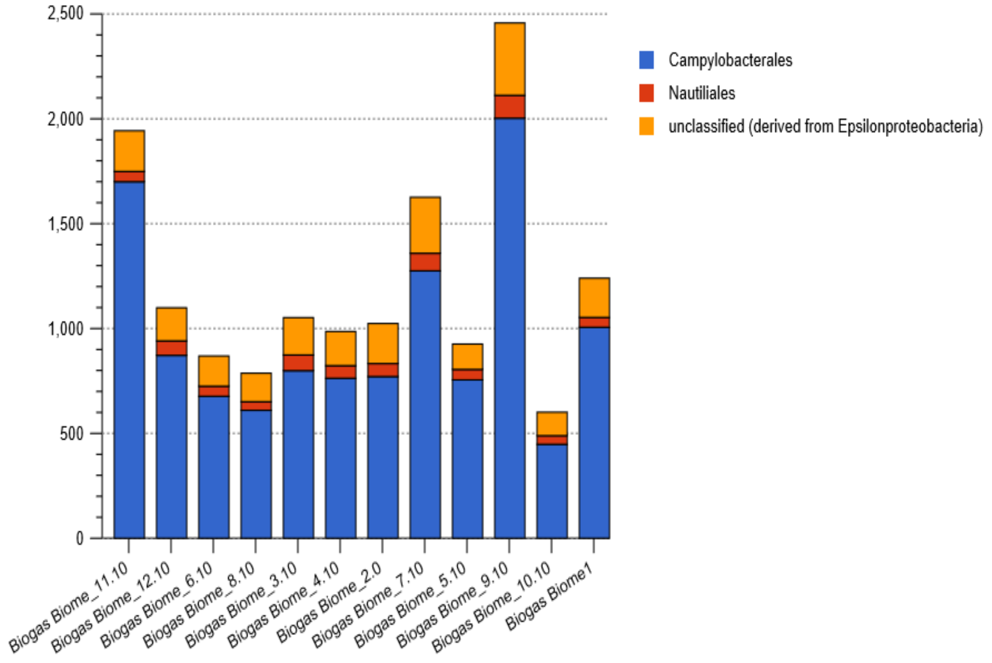

b

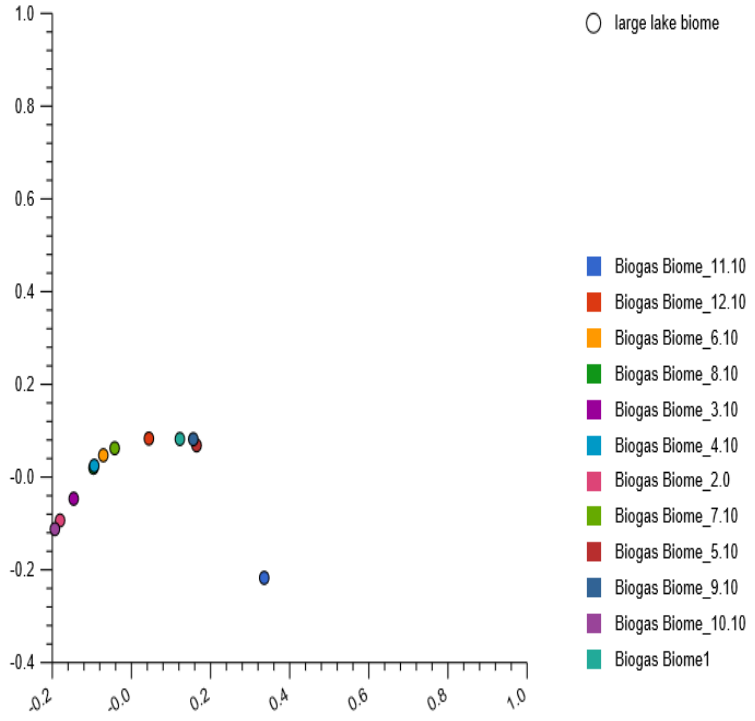

S10 Fig. Stacked barchat (a) showing two ε-Proteobacteria orders, relative abundances and their PCoA plot (b) based on Euclidean model. The PCoA plot for reactor 4 and 8 clustered on the upper left quadrant of the plot while those of reactor 5 and 9 clustered partially on the upper right quadrant.
