# Supplementary material for: Metagenomics survey unravels diversity of biogas microbiomes with potential to enhance productivity in Kenya
Source: PLoS One. 2021 Jan 4;16(1):e0244755. doi: 10.1371/journal.pone.0244755 (PMC7781671; doi:10.1371/journal.pone.0244755)
Supplement: S11 Fig — The stacked barchat revealing four Firmicutes classes, the relative abundances (a) and their PCoA plots, revealing variance among the twelve reactors based on the Euclidean model (b). The PCoA plots revealed close proximity of reactor 4 and 9 and reactor 1 and 8 nucleotide compositions, while the reactor 1 and 7 composition clustered partially on the plot. However, those of reactor 2, 10 and 12 were found to cluster on the lower left quadrant of the plot while other treatments’ compositions were found to reveal dissimilarity, except the composition of reactor 5 that were singly positioned on the upper left quadrant of the plot. (PDF) [file pone.0244755.s012.pdf]

a

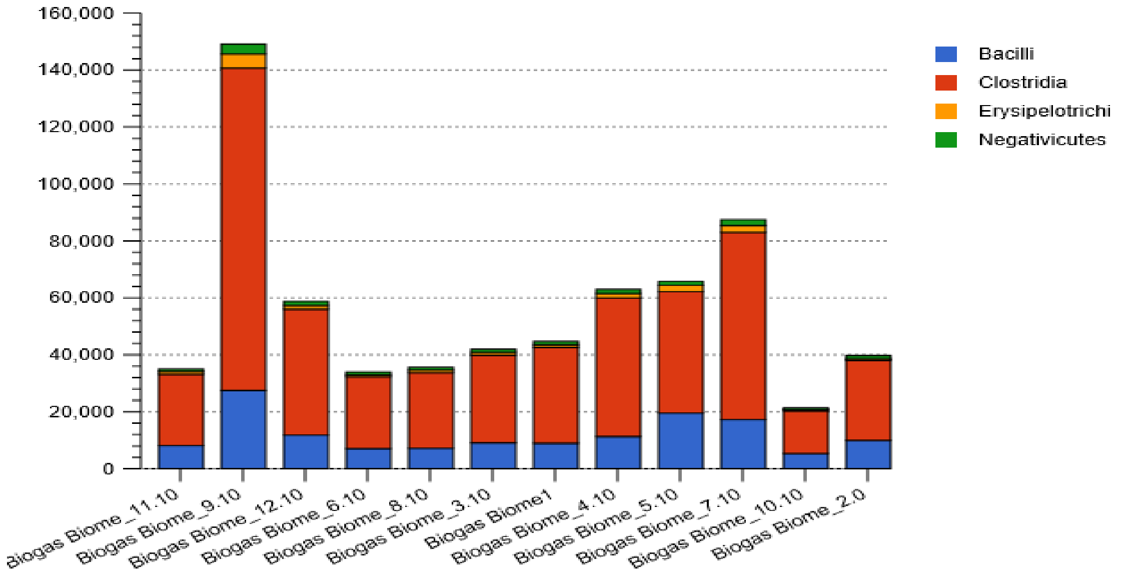

b

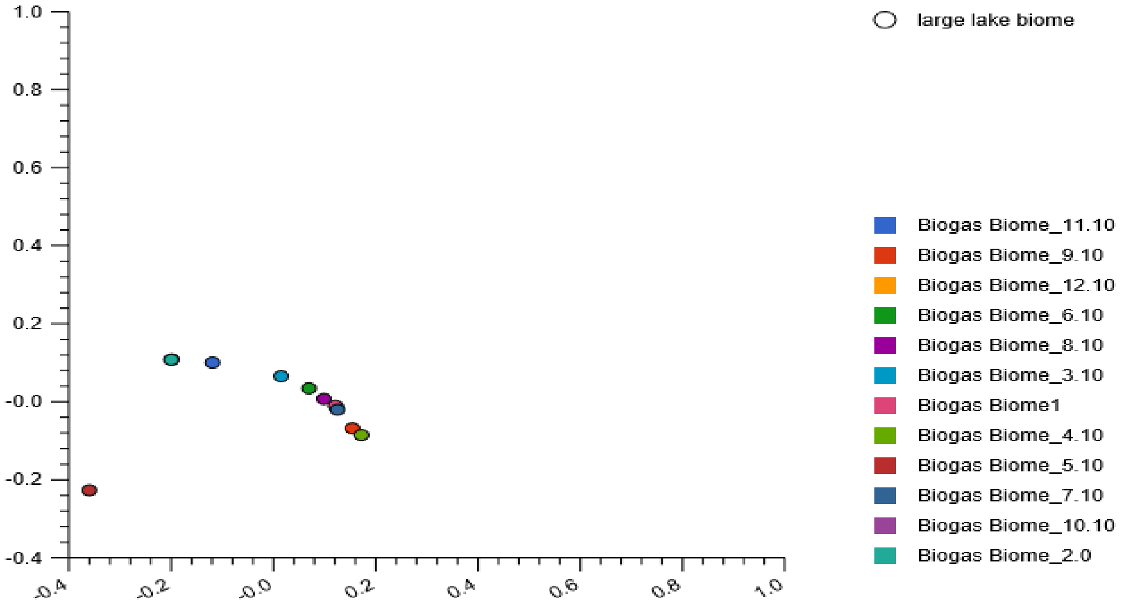

S11 Fig. The stacked barchat (a) revealing four *Firmicutes* classes, the relative abundances and their PCoA plots (b), revealing variance among the twelve reactors based on the Euclidean model. The plot revealed partial similarities between the composition of reactor 2 and 10, reactor 3 and 6, and reactor 7 and 12 while the nucleotide composition of reactor 1 and 9 almost clustered.
