# Supplementary material for: Metagenomics survey unravels diversity of biogas microbiomes with potential to enhance productivity in Kenya
Source: PLoS One. 2021 Jan 4;16(1):e0244755. doi: 10.1371/journal.pone.0244755 (PMC7781671; doi:10.1371/journal.pone.0244755)
Supplement: S12 Fig — Stacked barchat showing eight Firmicute’s orders, relative abundances (a) and their PCoA plot based on the Euclidean model (b). The plot revealed partial similarities between the composition of reactor 2 and 10, reactor 3 and 6, and reactor 7 and 12 while the nucleotide composition of reactor 1 and 9 almost clustered. (PDF) [file pone.0244755.s013.pdf]

a

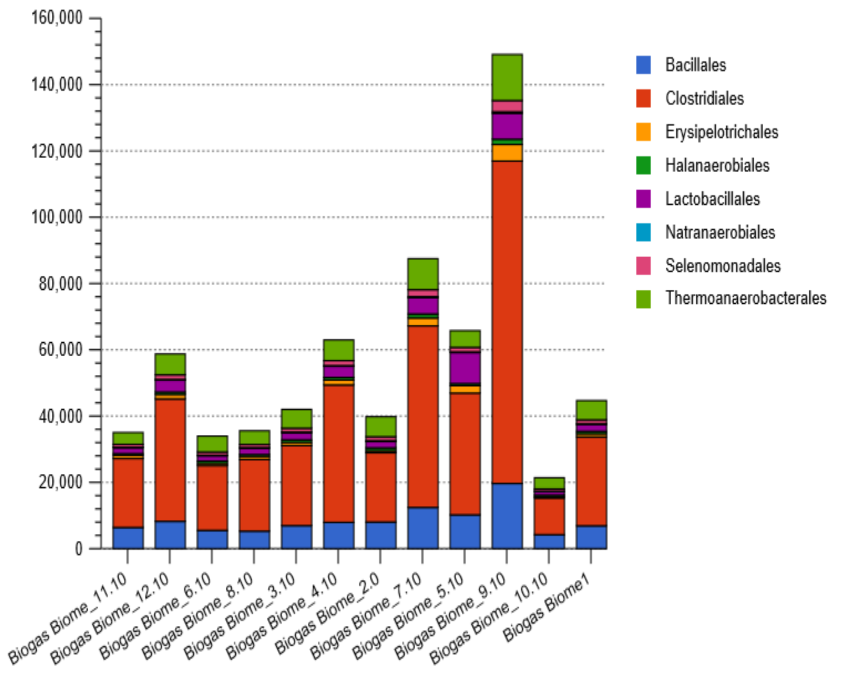

b

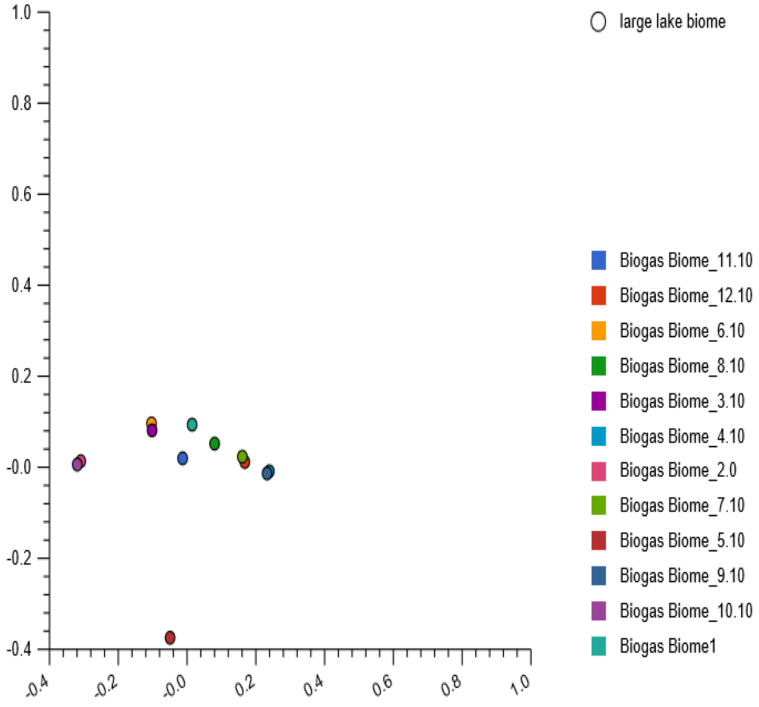

S12 Fig. Stacked barchat (a) showing eight *Firmicute'*s orders, relative abundances and their PCoA plot (b) based on the Euclidean model. The plot revealed partial similarities between the composition of reactor 2 and 10, reactor 3 and 6, and reactor 7 and 12 while the nucleotide composition of reactor 1 and 9 almost clustered.
