# Supplementary material for: Metagenomics survey unravels diversity of biogas microbiomes with potential to enhance productivity in Kenya
Source: PLoS One. 2021 Jan 4;16(1):e0244755. doi: 10.1371/journal.pone.0244755 (PMC7781671; doi:10.1371/journal.pone.0244755)
Supplement: S13 Fig — Stacked barchat showing the four Clostridia orders (a) and their PCoA plots, revealing nucleotide composition variations among the twelve reactors based on the Euclidean model (b). The plots revealed dissimilarities of the nucleotide composition among the majority of the treatments. However, the composition of reactor 5 and 9 were clustered partially on the upper left quadrant, while the nucleotide of reactor 7, 11 and12 clustered on the lower left quadrant of the plot. (PDF) [file pone.0244755.s014.pdf]

a

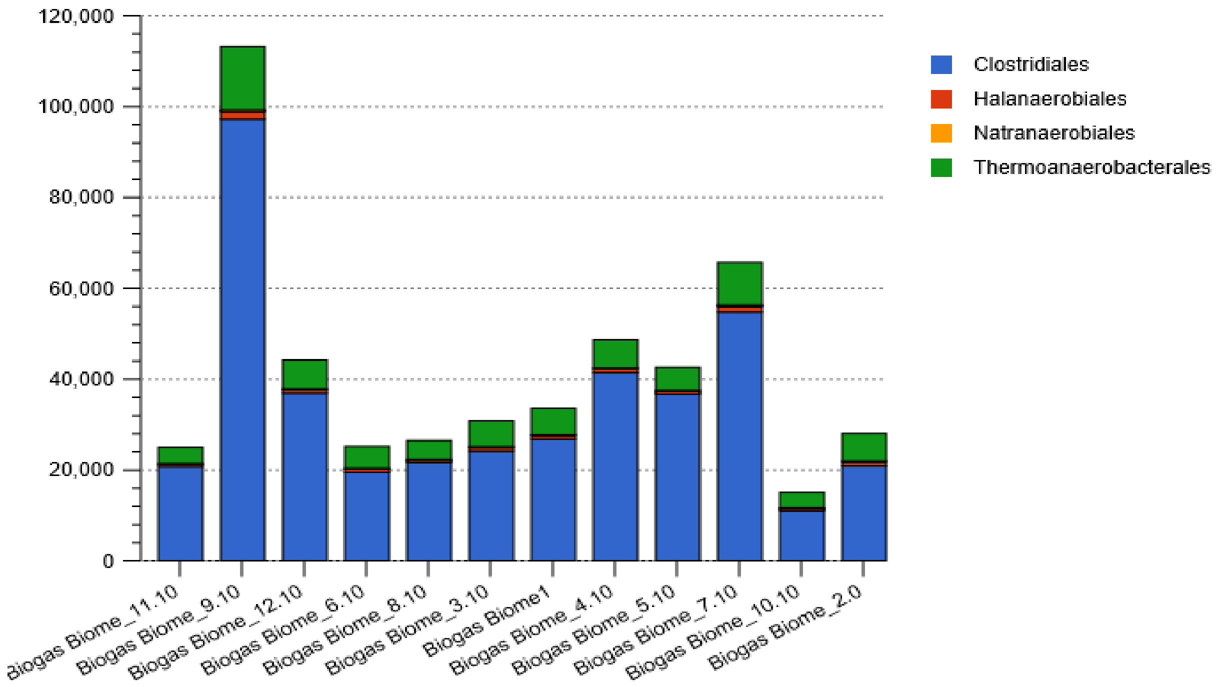


b

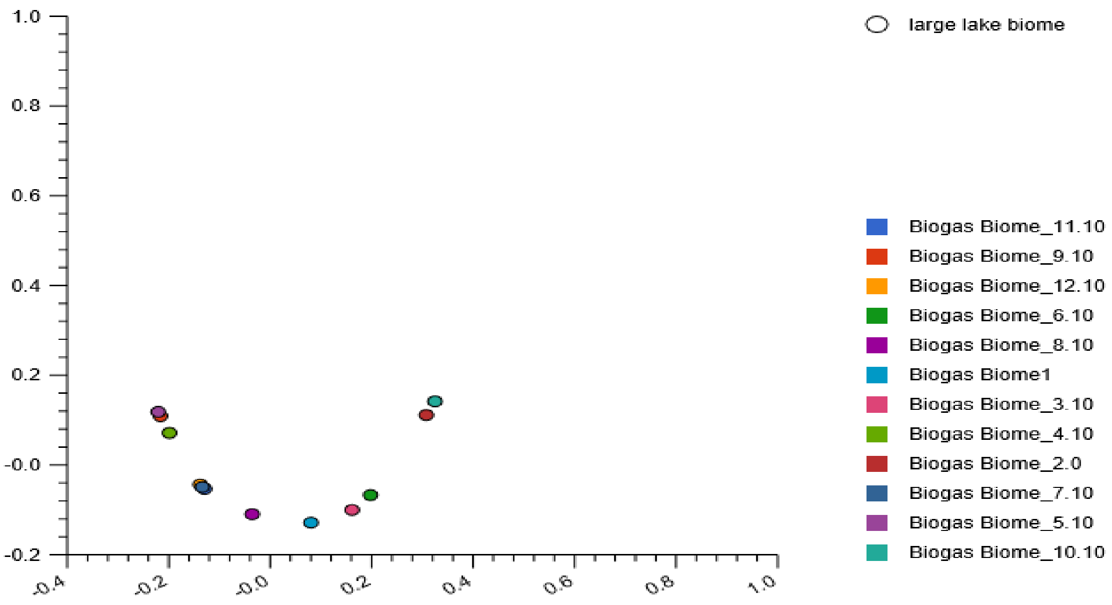


**S13 Fig. Stacked barchat (a) showing the four *Clostridia* orders and their PCoA plots (b), revealing nucleotide composition variations among the twelve reactors based on the Euclidean model**. The plots revealed dissimilarities of the nucleotide composition among the majority of the treatments. However, the composition of reactor 5 and 9 were clustered partially on the upper left quadrant, while the nucleotide of reactor 7, 11 and12 clustered on the lower left quadrant of the plot.
