# Supplementary material for: Metagenomics survey unravels diversity of biogas microbiomes with potential to enhance productivity in Kenya
Source: PLoS One. 2021 Jan 4;16(1):e0244755. doi: 10.1371/journal.pone.0244755 (PMC7781671; doi:10.1371/journal.pone.0244755)
Supplement: S14 Fig — Stacked barchat showing two Bacilli orders, relative abundances (a) and their PCoA plot based on the Euclidean model (b). The nucleotide composition varied among the treatments and pairwise partial clustering of the nucleotides was observed among the majority of the treatments, with an exception of reactor 5 and 8 communities that were distinctively positioned within the plot. (PDF) [file pone.0244755.s015.pdf]

a

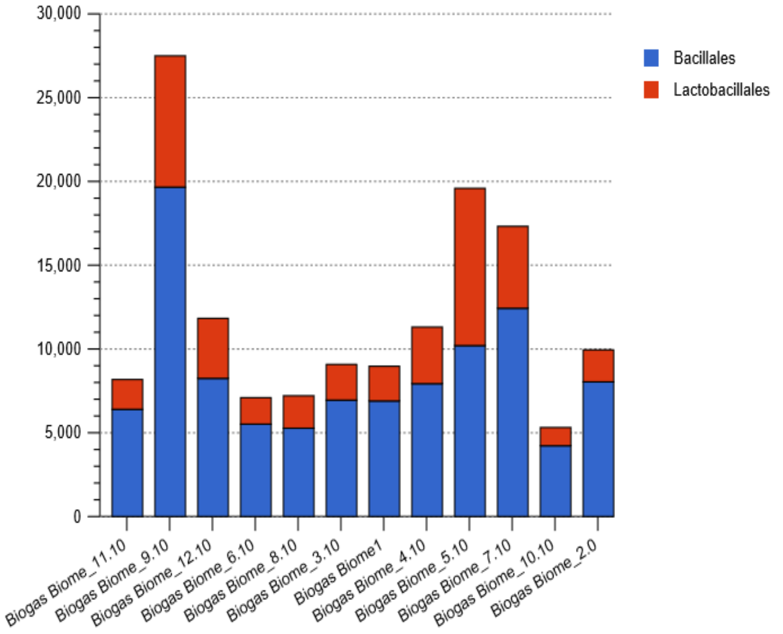

b

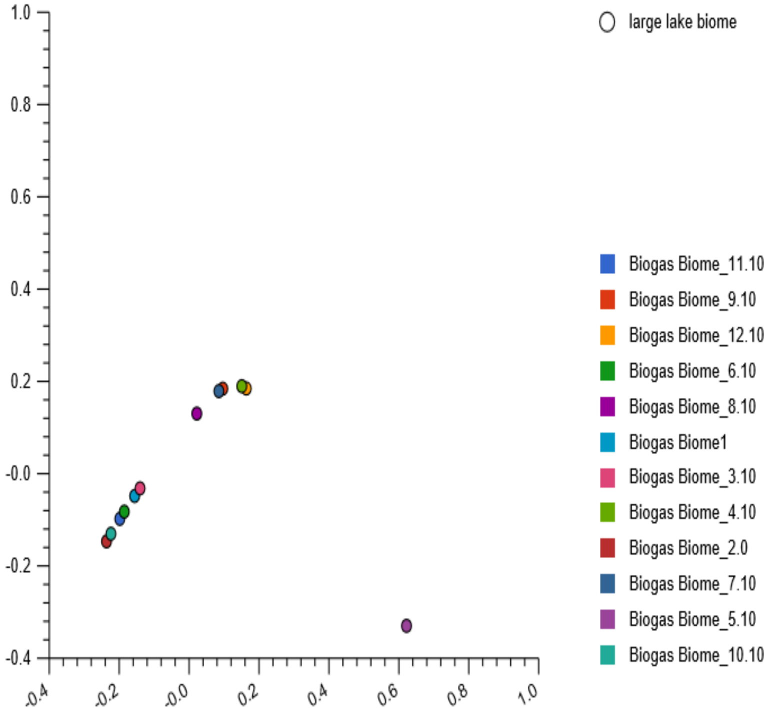

S14 Fig. Stacked barchat (a) showing two Bacilli orders, relative abundances and their PCoA plot (b) based on the Euclidean model. The nucleotide composition varied among the treatments and pairwise partial clustering of the nucleotides was observed among the majority of the treatments, with an exception of reactor 5 and 8 communities that were distinctively positioned within the plot.
