# Supplementary material for: Metagenomics survey unravels diversity of biogas microbiomes with potential to enhance productivity in Kenya
Source: PLoS One. 2021 Jan 4;16(1):e0244755. doi: 10.1371/journal.pone.0244755 (PMC7781671; doi:10.1371/journal.pone.0244755)
Supplement: S15 Fig — The stacked barchat revealing four Bacteroidetes classes, relative abundances (a) and their PCoA plots revealing nucleotide composition variation, based on the Euclidean model (b). The plots revealed partial clustering of reactor 2 and 10 and reactor 8 and 12 communities on the plot. However, the communities of reactor 4 and 9 were found to cluster on the lower right quadrant of the plot. (PDF) [file pone.0244755.s016.pdf]

a

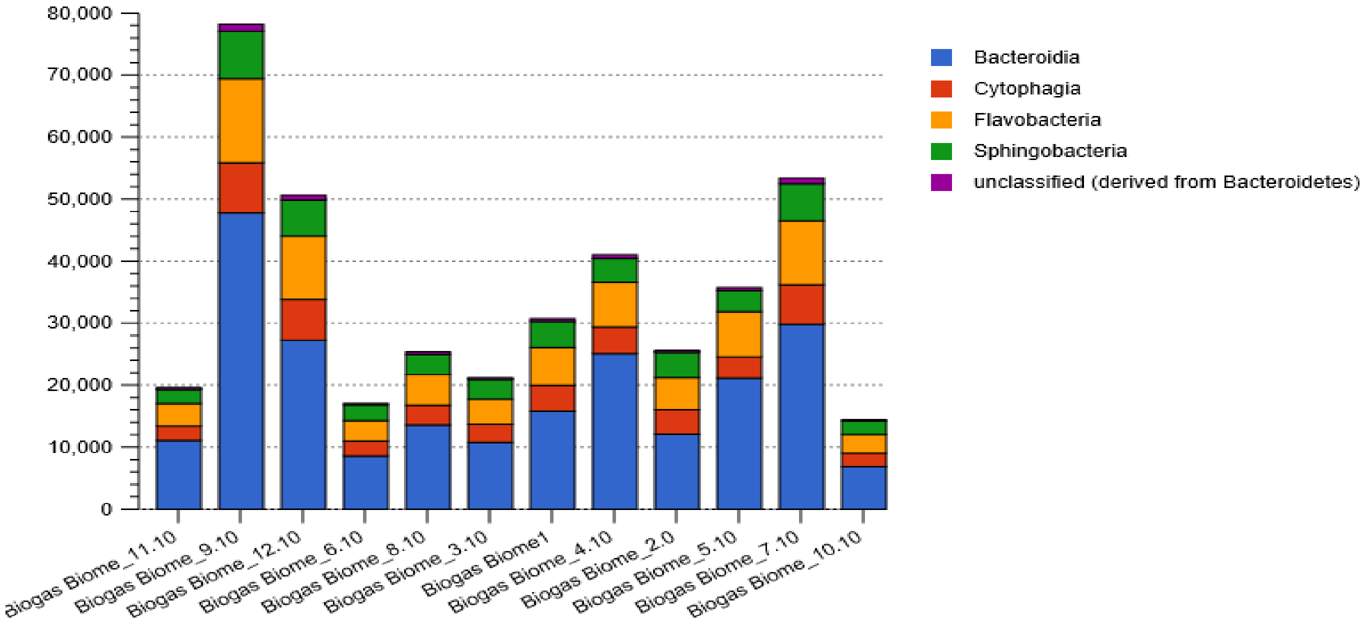

b

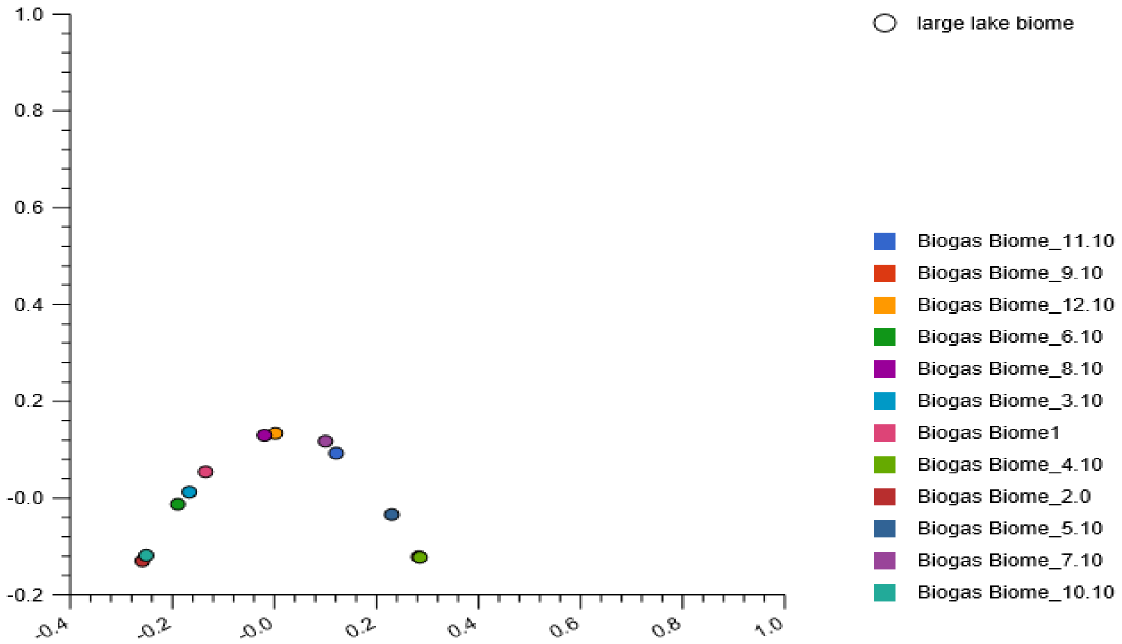

S15 Fig. The stacked barchat (a) revealing four *Bacteroidete*s classes, relative abundances and their PCoA plots (b) revealing nucleotide composition variation, based on the Euclidean model. The plots revealed partial clustering of reactor 2 and 10 and reactor 8 and 12 communities on the plot. However, the communities of reactor 4 and 9 were found to cluster on the lower right quadrant of the plot.
