# Supplementary material for: Metagenomics survey unravels diversity of biogas microbiomes with potential to enhance productivity in Kenya
Source: PLoS One. 2021 Jan 4;16(1):e0244755. doi: 10.1371/journal.pone.0244755 (PMC7781671; doi:10.1371/journal.pone.0244755)
Supplement: S16 Fig — Stacked barchat showing the four Bacteroidete’s orders, the relative abundances (a) and their PCoA plot based on the Euclidean model (b). The nucleotide composition of reactor 2 and 10 and those of reactor 8 and 12 clustered partially on the lower left quadrant of the plot while the composition of reactor 4 and 9 were revealed to cluster on the lower right quadrant of the plot. (PDF) [file pone.0244755.s017.pdf]

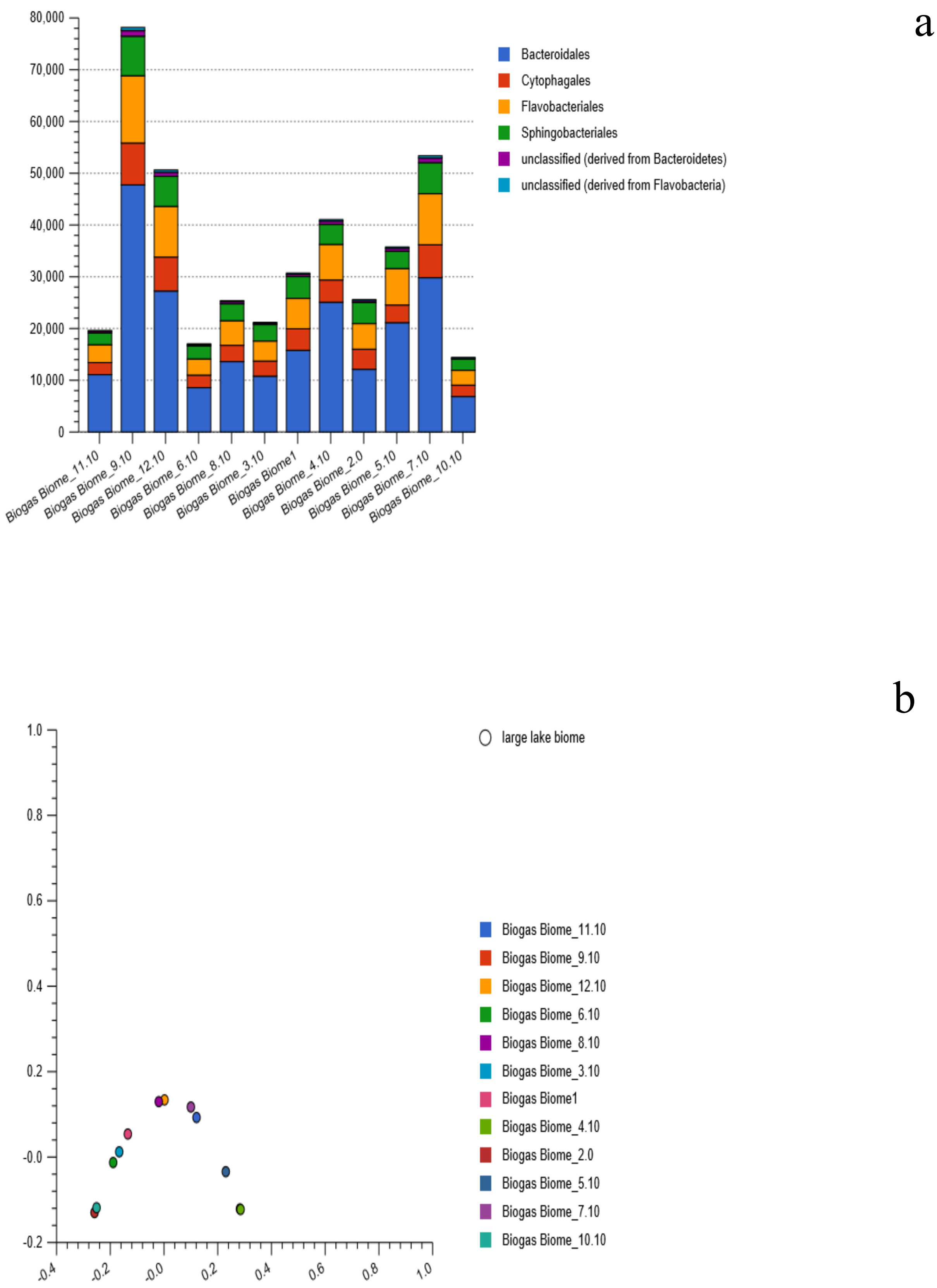

S16 Fig. Stacked barchat (a) showing the four *Bacteroidete*'s orders, the relative abundances and their PCoA plot (b) based on the Euclidean model. The nucleotide composition of reactor 2 and 10 and those of reactor 8 and 12 clustered partially on the lower left quadrant of the plot while the composition of reactor 4 and 9 were revealed to cluster on the lower right quadrant of the plot.
