# Supplementary material for: Metagenomics survey unravels diversity of biogas microbiomes with potential to enhance productivity in Kenya
Source: PLoS One. 2021 Jan 4;16(1):e0244755. doi: 10.1371/journal.pone.0244755 (PMC7781671; doi:10.1371/journal.pone.0244755)
Supplement: S17 Fig — Stacked barchat showing six Actinobacteria orders, the relative abundance (a) and their PCoA plot based on the Euclidean model (b). The nucleotide composition of reactor 7 and 8 positioned on the upper right quadrant of the plot were found to cluster partiall, while the nucleotide of reactor 1, 3, 5, 11 and 12 formed a cluster along the y-axis of the plot. (PDF) [file pone.0244755.s018.pdf]

a

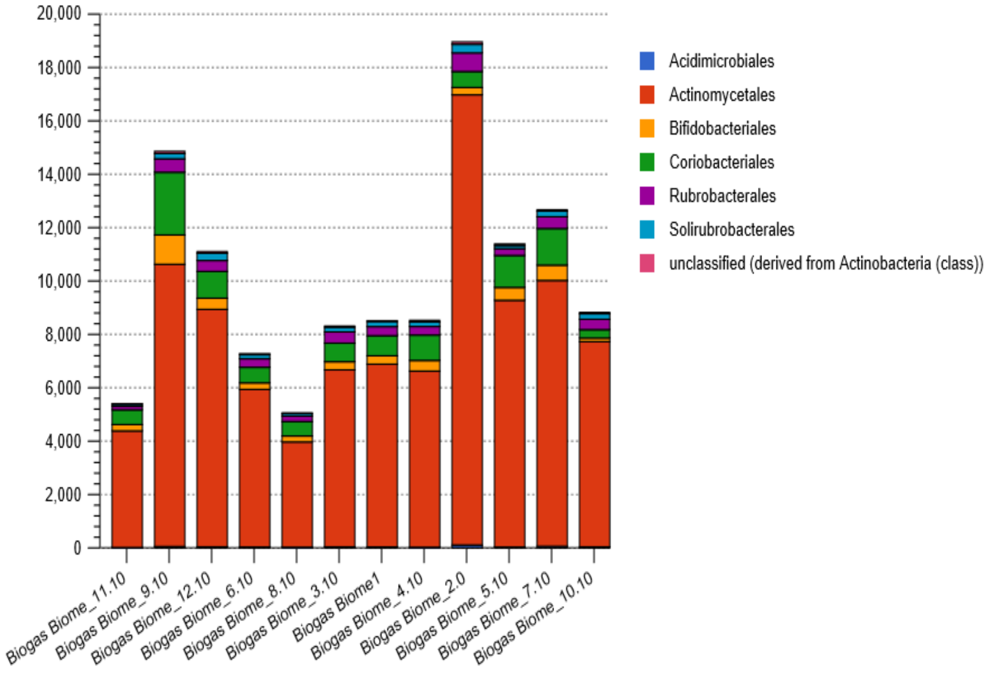

b

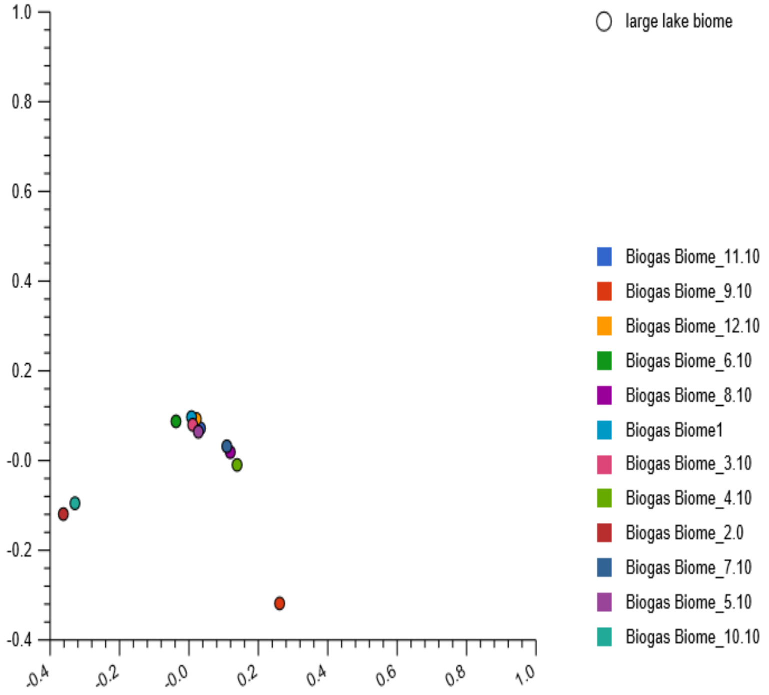

S17 Fig. Stacked barchat (a) showing six actinobacteria orders, the relative abundance and their PCoA plot (b) based on the Euclidean model. The nucleotide composition of reactor 7 and 8 positioned on the upper right quadrant of the plot were found to cluster partiall, while the nucleotide of reactor 1, 3, 5, 11 and 12 formed a cluster along the y-axis of the plot.
