# Supplementary material for: Metagenomics survey unravels diversity of biogas microbiomes with potential to enhance productivity in Kenya
Source: PLoS One. 2021 Jan 4;16(1):e0244755. doi: 10.1371/journal.pone.0244755 (PMC7781671; doi:10.1371/journal.pone.0244755)
Supplement: S18 Fig — The stacked barchat showing four Chloroflexi classes, relative abundances (a) and PCoA plot revealing variance among the twelve reactors, based on the Euclidean model (b). The plots revealed close proximity of reactor 1 and 7 nucleotide composition, and were positioned on the upper left quadrant of the plot. However, the composition of reactor 4, 5, 10 and 12 were found to cluster on the lower right quadrant of the plot. (PDF) [file pone.0244755.s019.pdf]

a

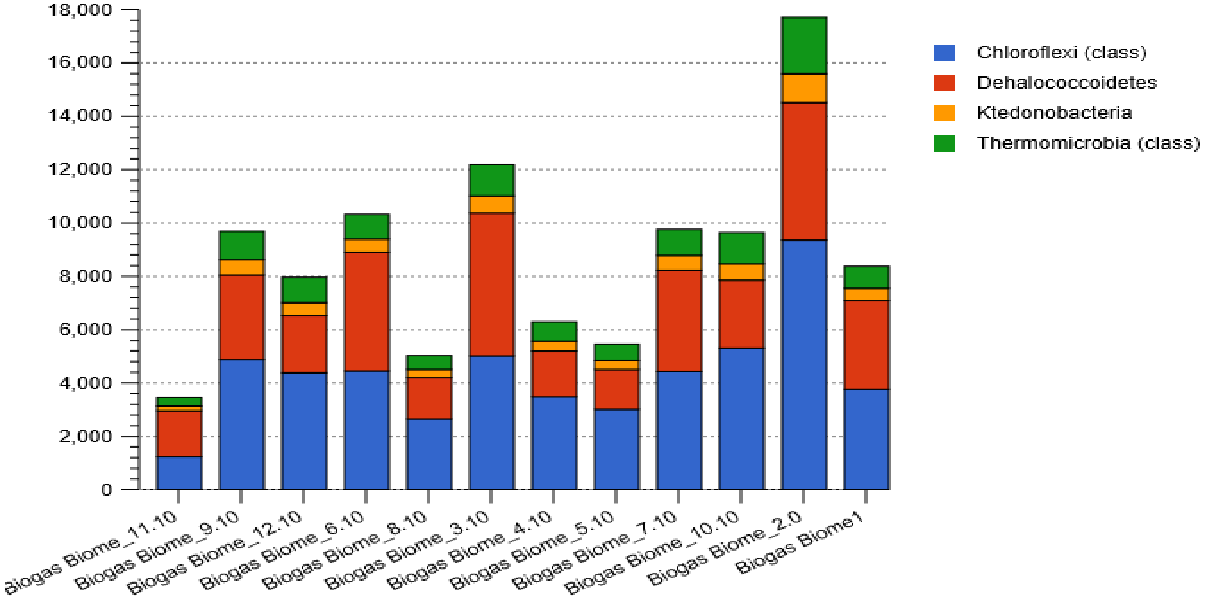

b

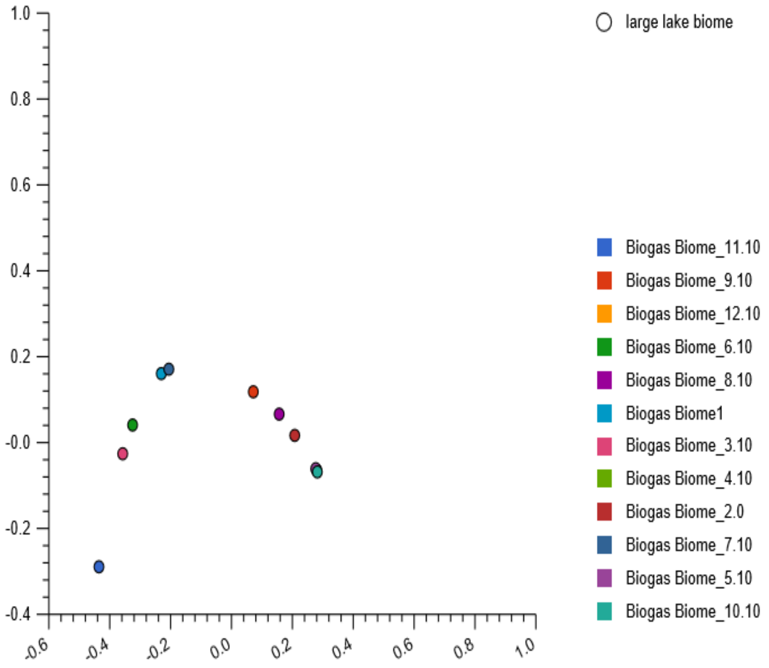

S18 Fig. The stacked barchat (a) showing four *Chloroflexi* classes, relative abundances and PCoA plot (b) revealing variance among the twelve reactors, based on the Euclidean model. The plots revealed close proximity of reactor 1 and 7 nucleotide composition, and were positioned on the upper left quadrant of the plot. However, the composition of reactor 4, 5, 10 and 12 were found to cluster on the lower right quadrant of the plot.
