# Supplementary material for: Metagenomics survey unravels diversity of biogas microbiomes with potential to enhance productivity in Kenya
Source: PLoS One. 2021 Jan 4;16(1):e0244755. doi: 10.1371/journal.pone.0244755 (PMC7781671; doi:10.1371/journal.pone.0244755)
Supplement: S19 Fig — Stacked barchat showing five Chloroflexi orders, relative abundances (a) and their PCoA plot based on the Euclidean model (b). The nucleotide composition of reactor 4, 5, 10 and 12 clustered, on the lower right quadrant of the plot, while the composition of reactor 1 and 7 were found to reveal close proximity on the upper left quadrant of the plot. (PDF) [file pone.0244755.s020.pdf]

a

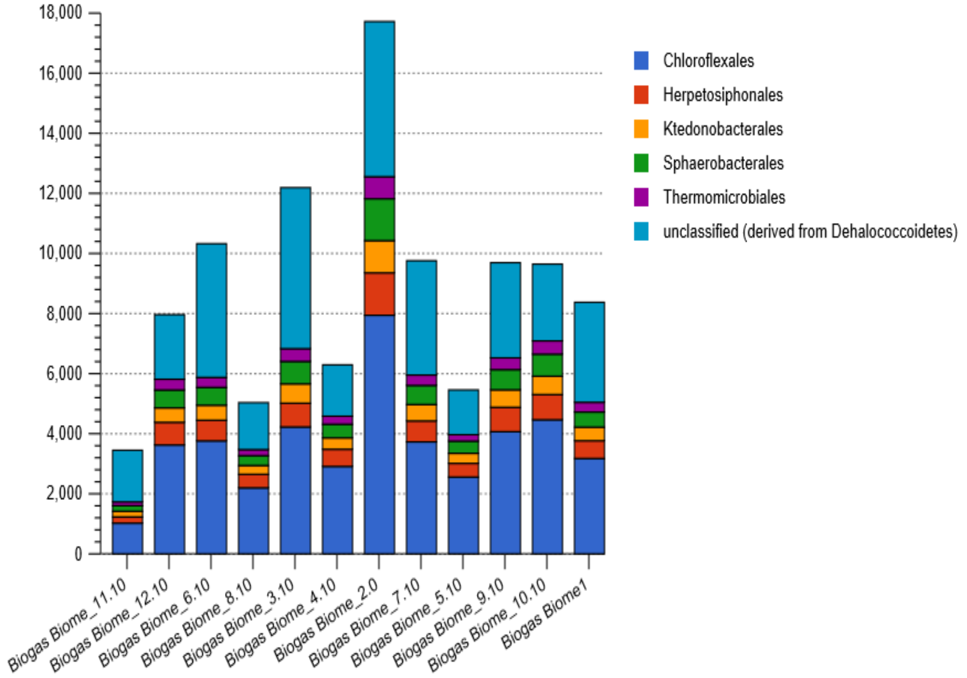


b

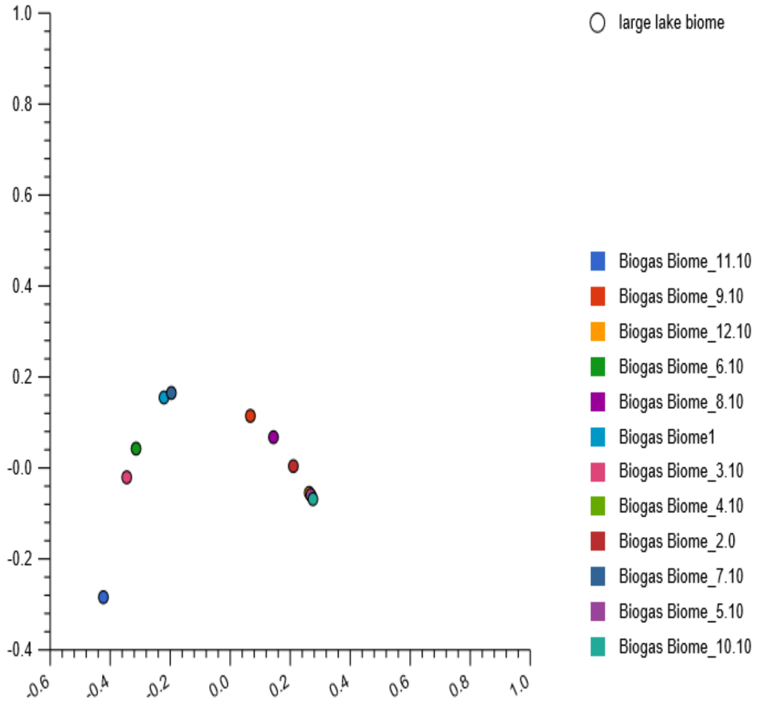


**S19 Fig. Stacked barchat showing five Chloroflexi orders, relative abundances and their PCoA plot based on the Euclidean model.** The nucleotide composition of reactor 4, 5, 10 and 12 clustered, on the lower right quadrant of the plot, while the composition of reactor 1 and 7 were found to reveal close proximity on the upper left quadrant of the plot.
