# Supplementary material for: Metagenomics survey unravels diversity of biogas microbiomes with potential to enhance productivity in Kenya
Source: PLoS One. 2021 Jan 4;16(1):e0244755. doi: 10.1371/journal.pone.0244755 (PMC7781671; doi:10.1371/journal.pone.0244755)
Supplement: S20 Fig — Stacked barchat showing two Chloroflexi class orders, relative abundances (a) and their PCoA plot based on their Euclidean model (b). The nucleotide composition of reactor 1 and 5 (clustered; upper right quadrant) and those of reactor 3 and 7 (clustered; lower right quadrant) were found to similar. Notably, the nucleotide reads of reactor 1 and 10 were revealed to cluster partially with the composition of reactor 3 and 7. Similarly the nucleotides of reactor 8, 11 and 12 clustered partially on the upper left quadrant of the plot. (PDF) [file pone.0244755.s021.pdf]

a

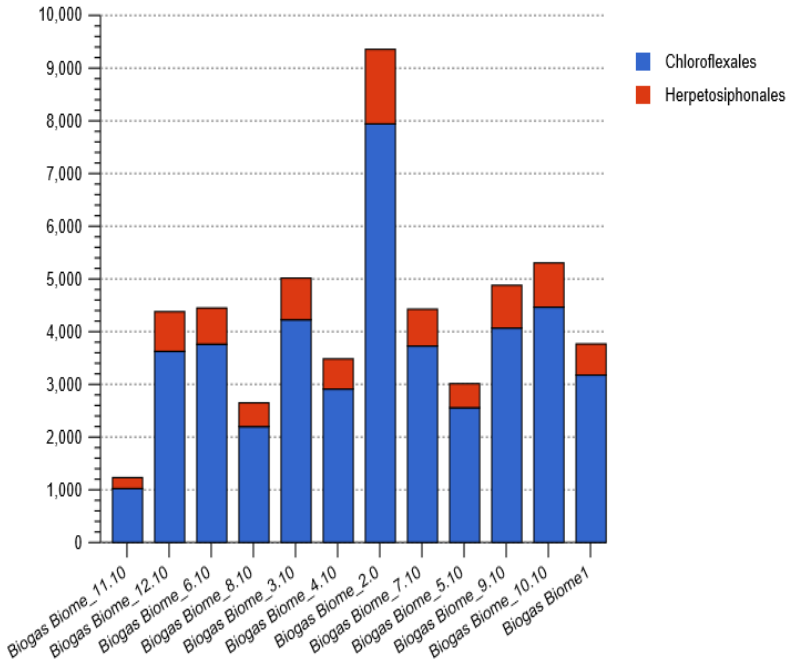

b

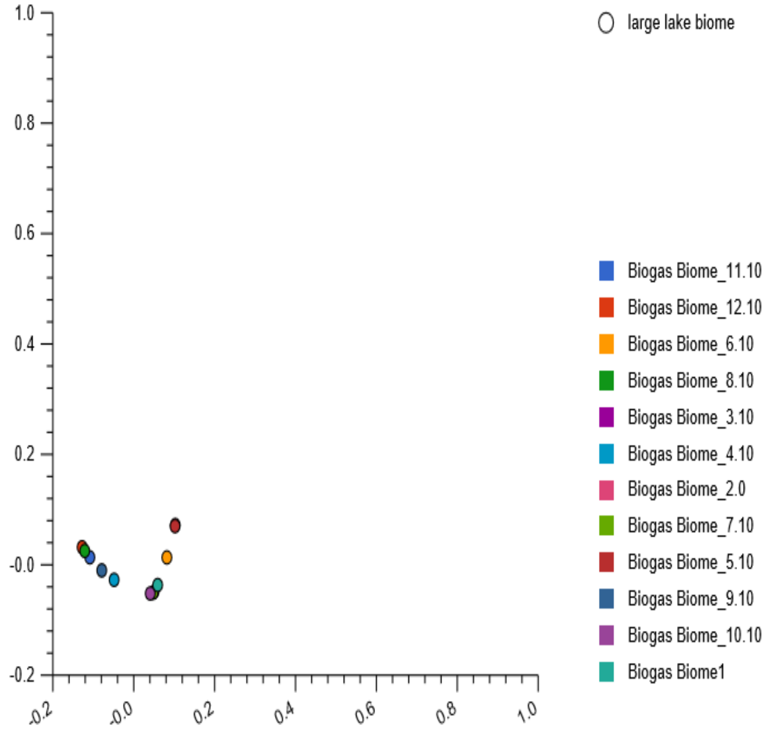

S20 Fig. Stacked barchat (a) showing two Chloroflexi class orders, relative abundances and their PCoA plot (b) based on their Euclidean model. The nucleotide composition of reactor 1 and 5 (clustered; upper right quadrant) and those of reactor 3 and 7 (clustered; lower right quadrant) were found to similar. Notably, the nucleotide reads of reactor 1 and 10 were revealed to cluster partially with the composition of reactor 3 and 7. Similarly the nucleotides of reactor 8, 11 and 12 clustered partially on the upper left quadrant of the plot.
