# Supplementary material for: Metagenomics survey unravels diversity of biogas microbiomes with potential to enhance productivity in Kenya
Source: PLoS One. 2021 Jan 4;16(1):e0244755. doi: 10.1371/journal.pone.0244755 (PMC7781671; doi:10.1371/journal.pone.0244755)
Supplement: S21 Fig — Stacked barchat showing the two Thermomicrobia orders, relative abundances (a) and their PCoA plot based on the Euclidean model (b). The PCoA plot revealed dissimilarities of the nucleotide compositions among the twelve studied treatments. (PDF) [file pone.0244755.s022.pdf]

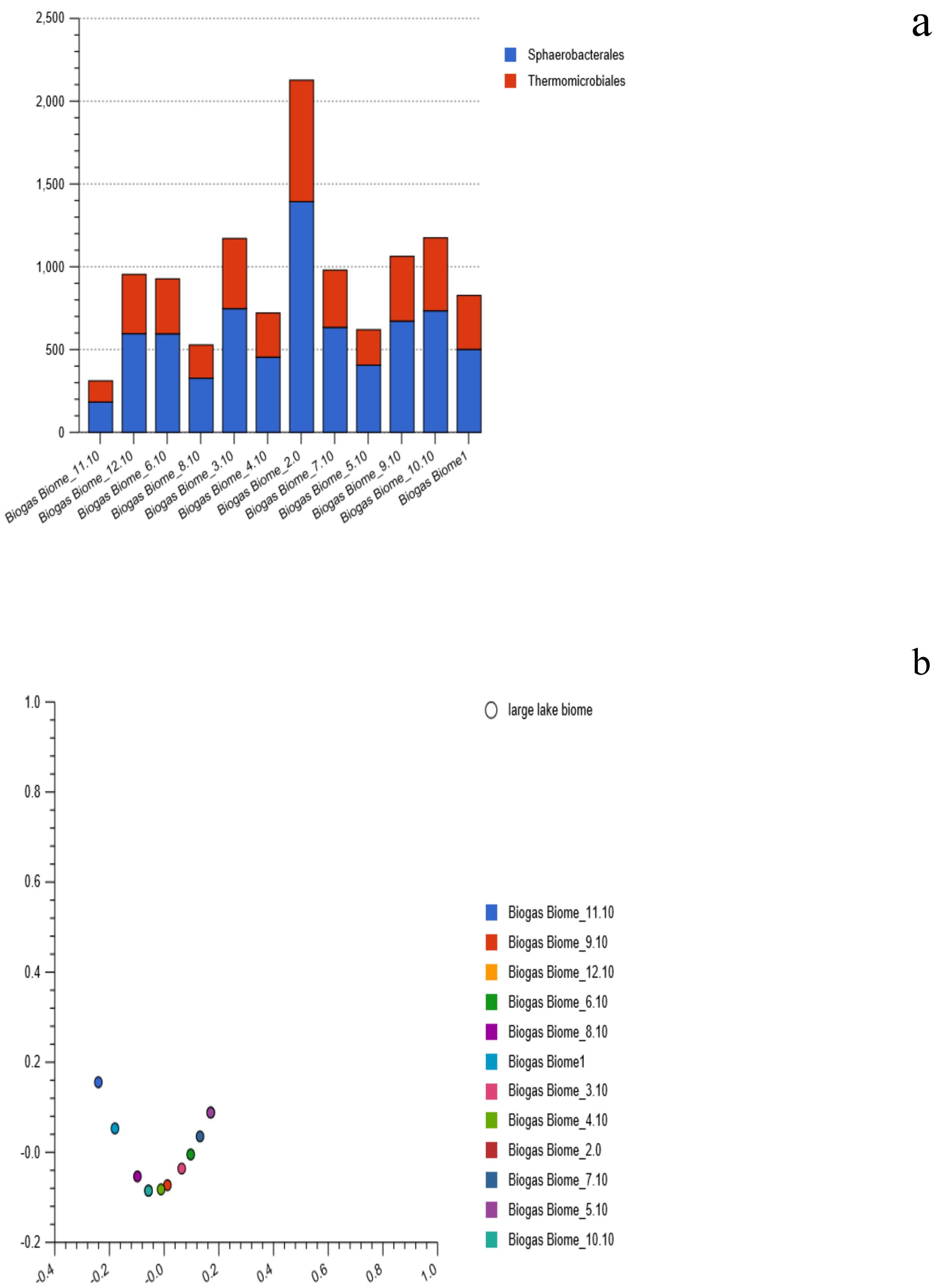

S21 Fig. Stacked barchat (a) showing the two *Thermomicrobia* orders, relative abundances and their PCoA plot (b) based on the Euclidean model. The PCoA plot revealed dissimilarities of the nucleotide compositions among the twelve studied treatments.
