# Supplementary material for: Metagenomics survey unravels diversity of biogas microbiomes with potential to enhance productivity in Kenya
Source: PLoS One. 2021 Jan 4;16(1):e0244755. doi: 10.1371/journal.pone.0244755 (PMC7781671; doi:10.1371/journal.pone.0244755)
Supplement: S22 Fig — Stacked barchat showing Cyanobacteria class, relative abundances (a) and their PCoA plot based on the Euclidean model (b). The plot revealed dissimilarities of the nucleotide composition among the treatments and only the nucleotides of three treatments (reactor 3, 7 and 10) that were found to be distinct within the plot. (PDF) [file pone.0244755.s023.pdf]

a

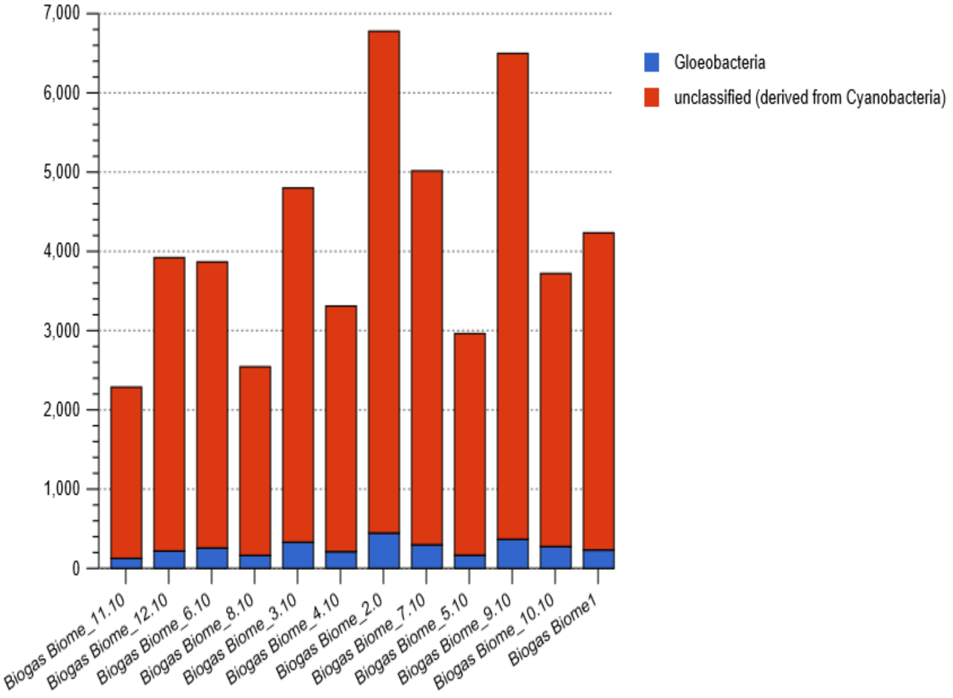

b

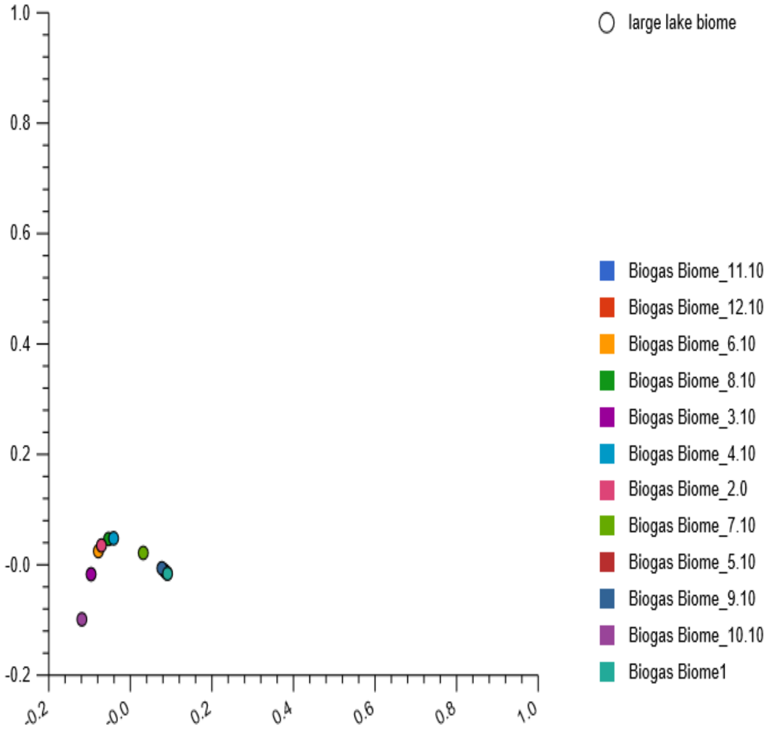

S22 Fig. Stacked barchat (a) showing *Cyanobacteria* class, relative abundances and their PCoA plot (b) based on the Euclidean model. The plot revealed dissimilarities of the nucleotide composition among the treatments and only the nucleotides of three treatments (reactor 3, 7 and 10) that were found to be distinct within the plot.
