# Supplementary material for: Metagenomics survey unravels diversity of biogas microbiomes with potential to enhance productivity in Kenya
Source: PLoS One. 2021 Jan 4;16(1):e0244755. doi: 10.1371/journal.pone.0244755 (PMC7781671; doi:10.1371/journal.pone.0244755)
Supplement: S23 Fig — Stacked barchat showing the five Cyanobacteria orders, relative abundances (a) and their PCoA plot based on the Euclidean model (b). The PCoA plot revealed four out of twelve reactors that were distinctively located in plots. Other treatments formed partial clusters or clustered within the plots. (PDF) [file pone.0244755.s024.pdf]

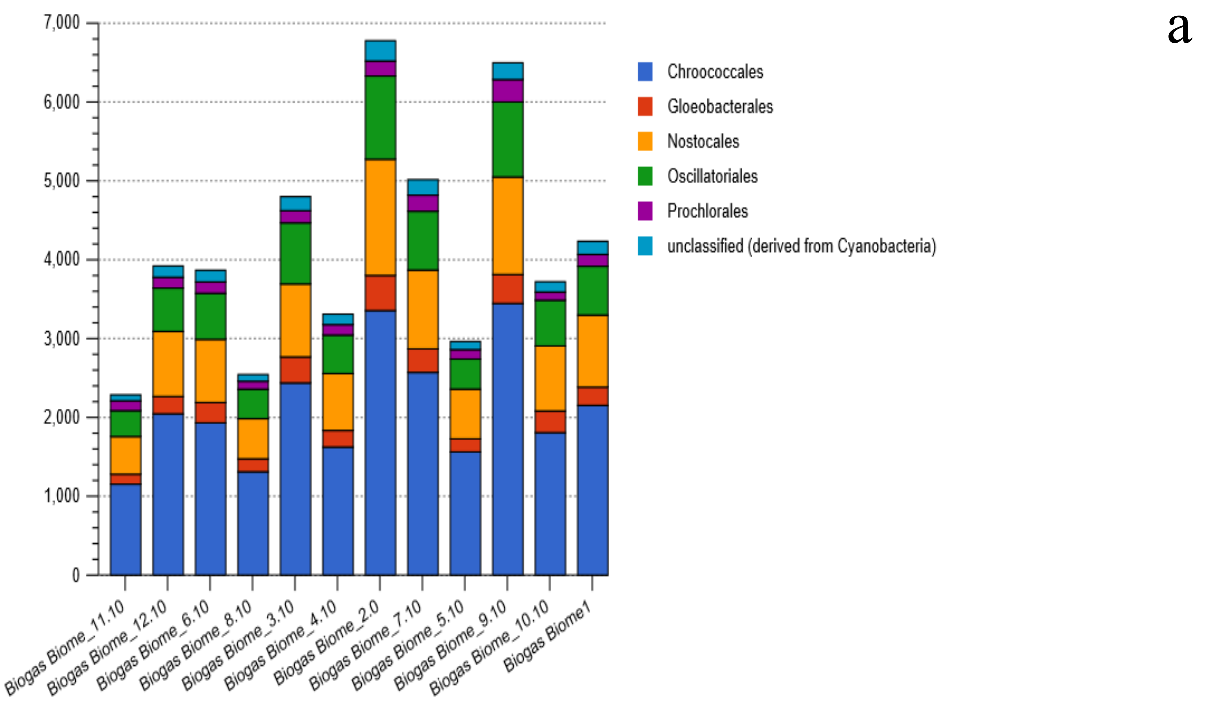

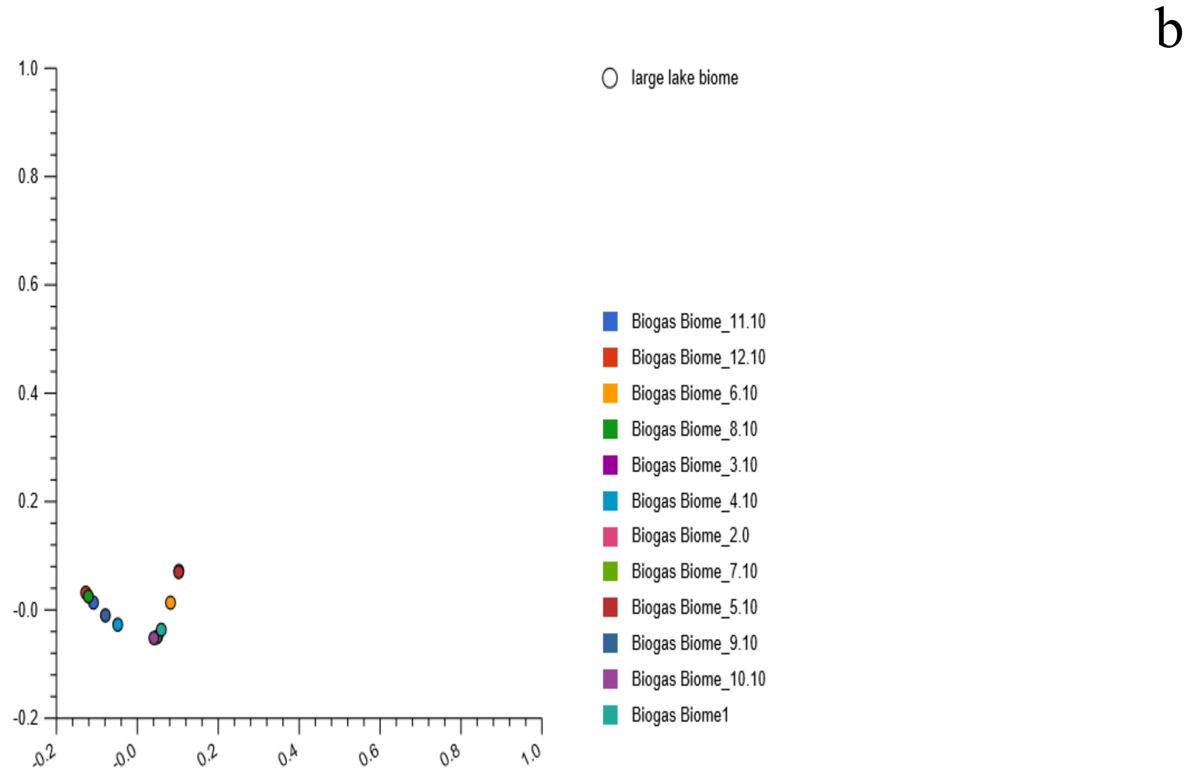

S23 Fig. Stacked barchat (a) showing the five *Cyanobacteria* orders, relative abundances and their PCoA plot (b) based on the Euclidean model. The PCoA plot revealed four out of twelve reactors that were distinctively located in plots. Other treatments formed partial clusters or clustered within the plots.
