# Supplementary material for: Metagenomics survey unravels diversity of biogas microbiomes with potential to enhance productivity in Kenya
Source: PLoS One. 2021 Jan 4;16(1):e0244755. doi: 10.1371/journal.pone.0244755 (PMC7781671; doi:10.1371/journal.pone.0244755)
Supplement: S24 Fig — Stacked barchat showing the four affiliates of the Unclassified Cyanobacteria nucleotide reads, relative abundances (a) and their PCoA plots based on the Euclidean model (b). The plot revealed dissimilarities among the treatments, all distributed in the four plot quadrant. (PDF) [file pone.0244755.s025.pdf]

a

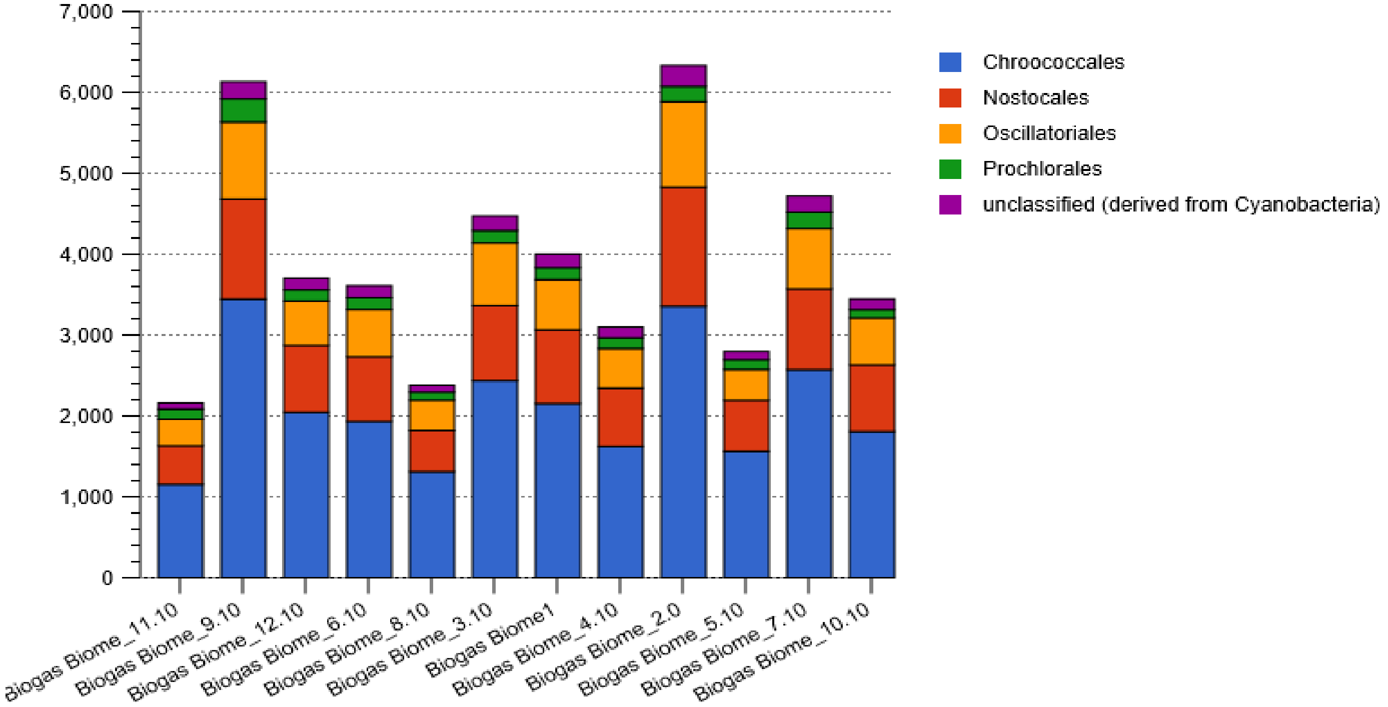

b

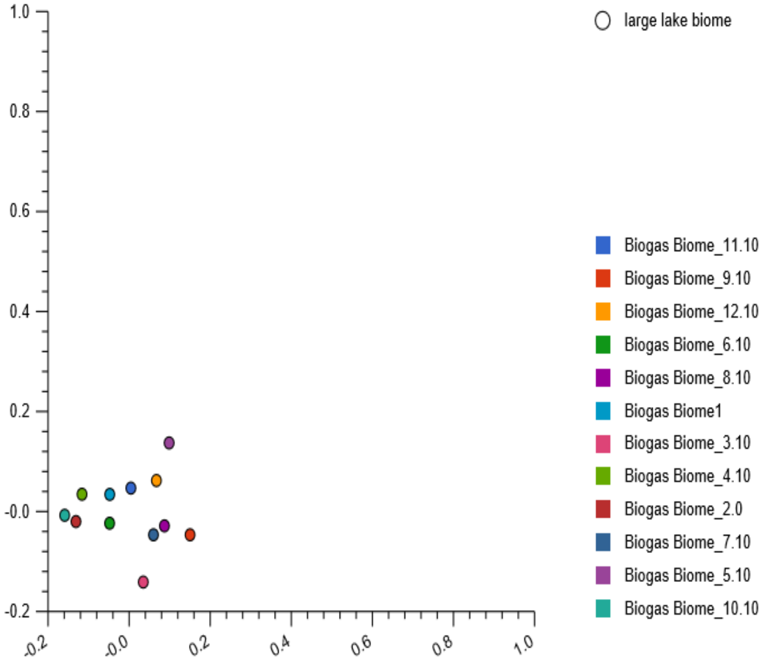

S24 Fig. Stacked barchat (a) showing the four affiliates of the Unclassified *Cyanobacteria* nucleotide reads, relative abundances and their PCoA plots (b), based on the Euclidean model. The plot revealed dissimilarities among the treatments, all distributed in the four plot quadrant.
