# Supplementary material for: Metagenomics survey unravels diversity of biogas microbiomes with potential to enhance productivity in Kenya
Source: PLoS One. 2021 Jan 4;16(1):e0244755. doi: 10.1371/journal.pone.0244755 (PMC7781671; doi:10.1371/journal.pone.0244755)
Supplement: S25 Fig — The stacked barchat showing the two Acidobacteria classes, relative abundances (a) and their PCoA plots for their nucleotide composition based on the Euclidean model (b). The nucleotide composition of reactor 1 and 3 clustered partially on the lower right quadrant of the plot, reactor 10 and 11 compositions were positioned in close proximity on the upper right quadrant of the plot while the nucleotides of reactor 2, 8 and 12 were found to cluster on the upper right quadrant of the plot. (PDF) [file pone.0244755.s026.pdf]

a

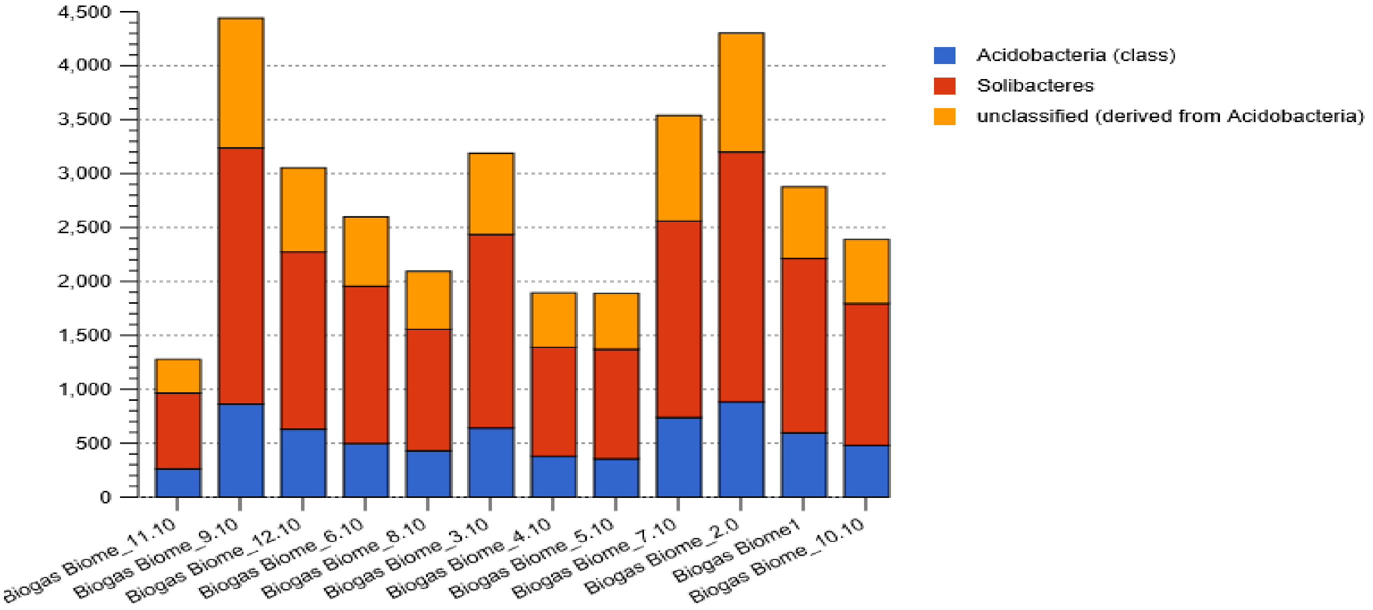

b

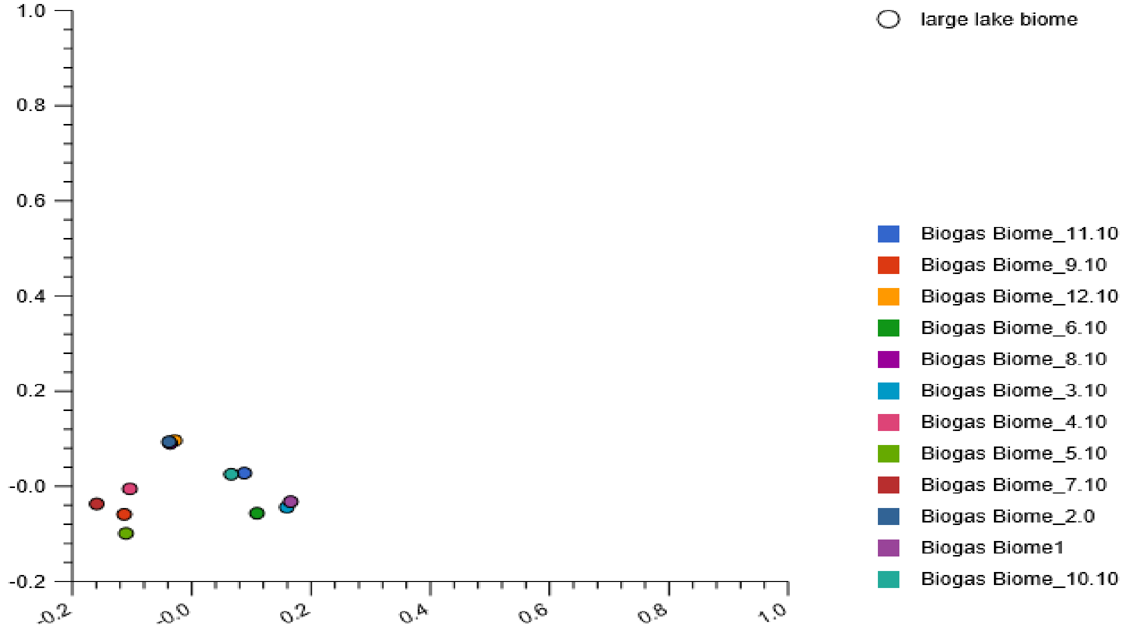

S25 Fig. The stacked barchat (a) showing the two *Acidobacteria* classes, relative abundances and their PCoA plots (b) for their nucleotide composition. The nucleotide composition of reactor 1 and 3 clustered partially on the lower right quadrant of the plot, reactor 10 and 11 compositions were positioned in close proximity on the upper right quadrant of the plot while the nucleotides of reactor 2, 8 and 12 were found to cluster on the upper right quadrant of the plot.
