# Supplementary material for: Metagenomics survey unravels diversity of biogas microbiomes with potential to enhance productivity in Kenya
Source: PLoS One. 2021 Jan 4;16(1):e0244755. doi: 10.1371/journal.pone.0244755 (PMC7781671; doi:10.1371/journal.pone.0244755)
Supplement: S26 Fig — The stacked barchat showing the two Acidobacteria orders, relative abundances (a) and their PCoA plots for their nucleotide composition based on Euclidean model (b). The composition of reactor 1 and 3 clustered partially on the lower right quadrant of the plot, those identified in reactor 10 and 11 were in close proximity, positioned on the upper right quadrant of the plot while the composition of reactor 2, 8 and 12 clustered in the upper right quadrant of the plot. (PDF) [file pone.0244755.s027.pdf]

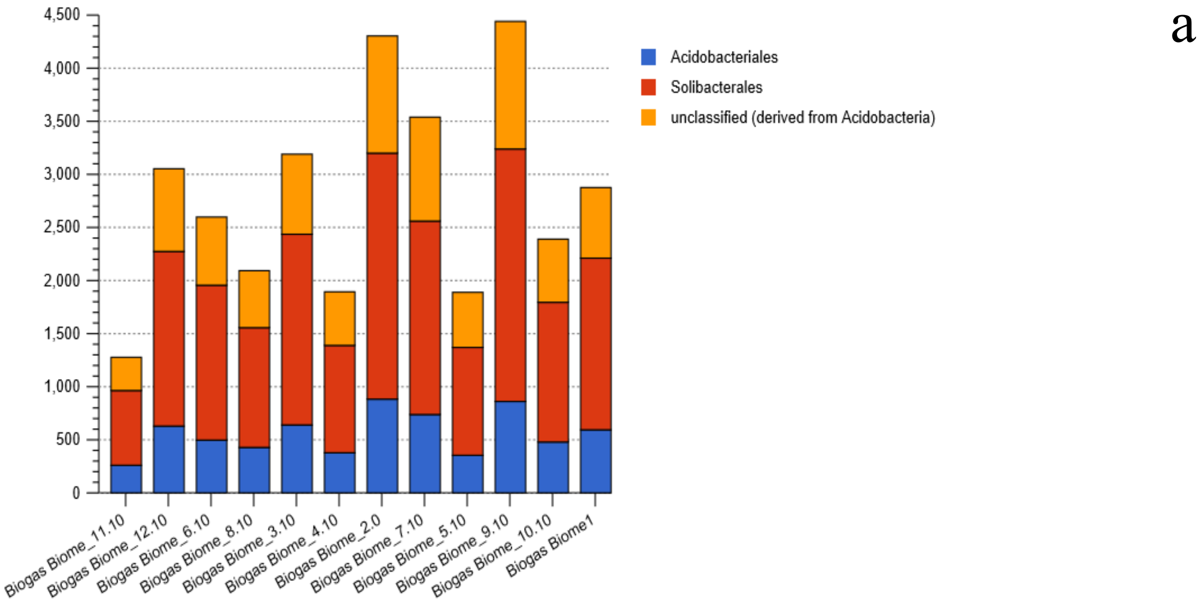

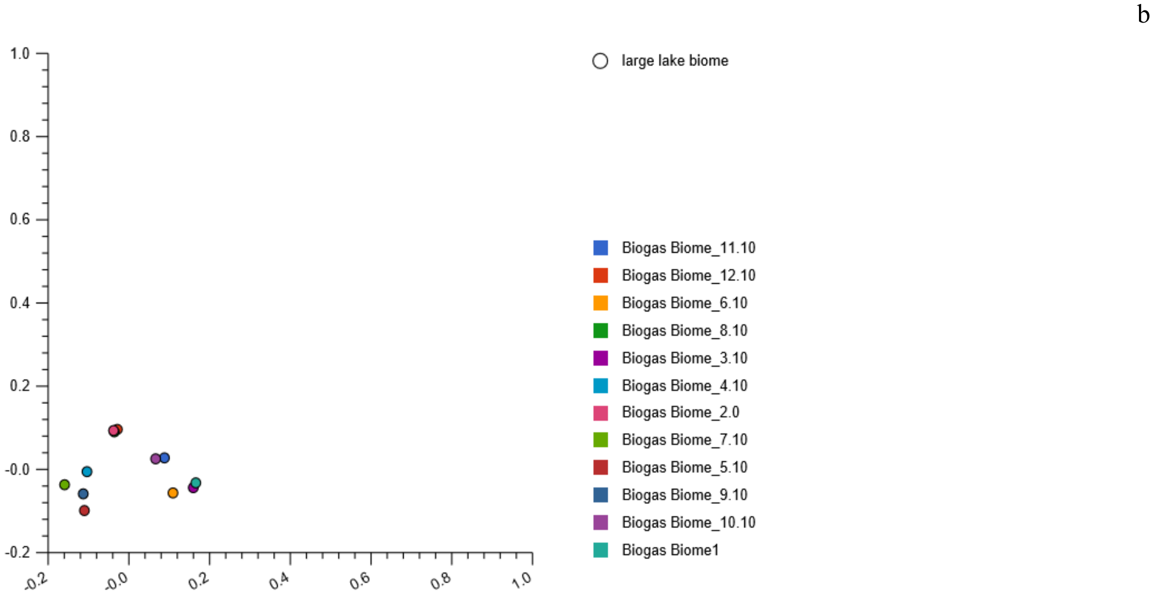

S26 Fig. The stacked barchat (a) showing the two *Acidobacteria* orders, relative abundances and their PCoA plots (b) for their nucleotide composition. The composition of reactor 1 and 3 clustered partially on the lower right quadrant of the plot, those identified in reactor 10 and 11 were in close proximity, positioned on the upper right quadrant of the plot while the composition of reactor 2, 8 and 12 clustered in the upper right quadrant of the plot.
