# Supplementary material for: Metagenomics survey unravels diversity of biogas microbiomes with potential to enhance productivity in Kenya
Source: PLoS One. 2021 Jan 4;16(1):e0244755. doi: 10.1371/journal.pone.0244755 (PMC7781671; doi:10.1371/journal.pone.0244755)
Supplement: S27 Fig — Stacked barchat showing two Deinococcus-Thermus orders, the relative abundances (a) and the PCoA plots based on the Euclidean model (b). The PCoA plots revealed clustering of the nucleotide composition of reactor 3 and 6, and those in reactor 7 and 9 on the upper left quadrant and lower left quadrant of the plot respectively. However, those identified in reactor 4 were in close proximity with those of reactor 7. The composition of reactor 11 and 12 partially clustered on the lower right quadrant of the plot. The nucleotides of reactor 2 and 10 were the only nucleotide reads located in the upper right quadrant of the plot. (PDF) [file pone.0244755.s028.pdf]

a

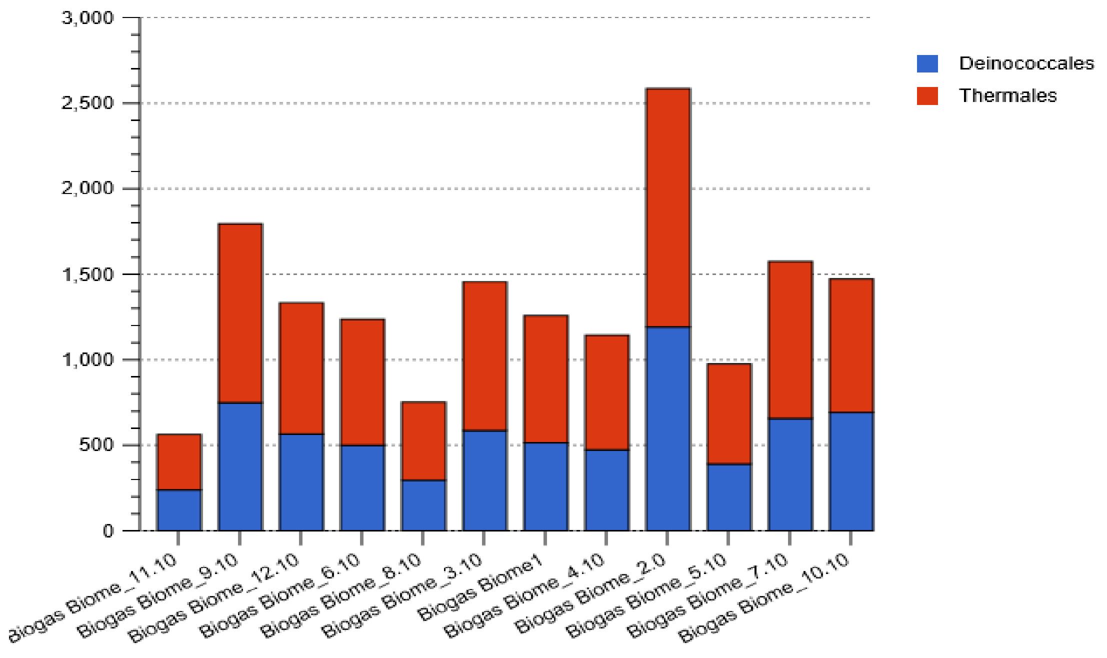

b

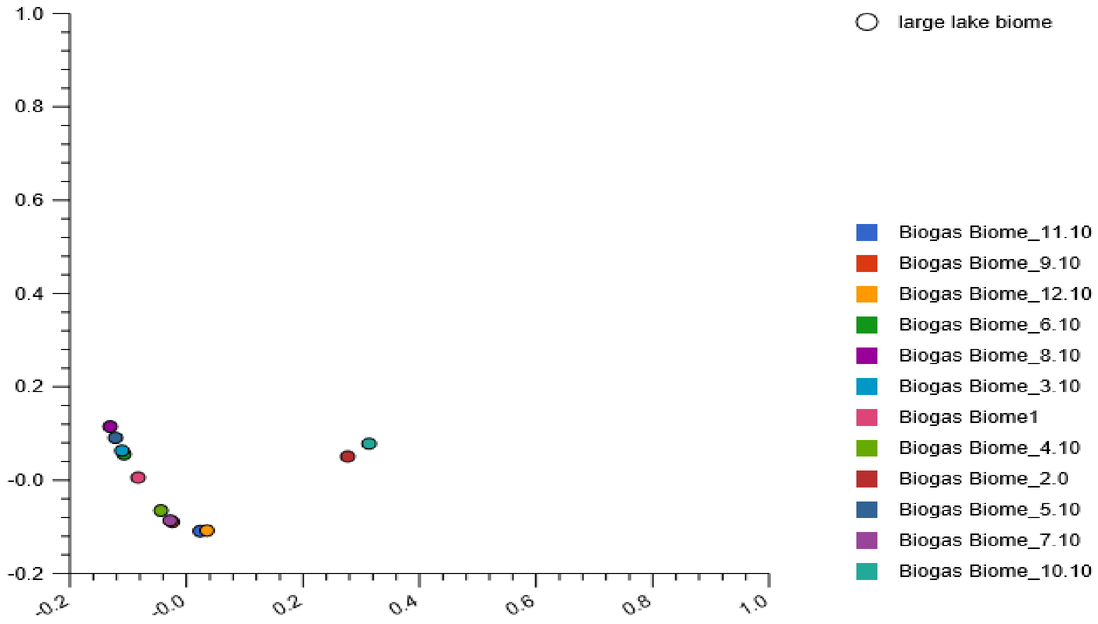

S27 Fig. Stacked barchat (a) showing two *Deinococcus-Thermus* orders, the relative abundances and the PCoA plots (b) based on the Euclidean model. The PCoA plots revealed clustering of the nucleotide composition of reactor 3 and 6, and those in reactor 7 and 9 on the upper left quadrant and lower left quadrant of the plot respectively. However, those identified in reactor 4 were in close proximity with those of reactor 7. The composition of reactor 11 and 12 partially clustered on the lower right quadrant of the plot. The nucleotides of reactor 2 and 10 were the only nucleotide reads located in the upper right quadrant of the plot.
