# Supplementary material for: Metagenomics survey unravels diversity of biogas microbiomes with potential to enhance productivity in Kenya
Source: PLoS One. 2021 Jan 4;16(1):e0244755. doi: 10.1371/journal.pone.0244755 (PMC7781671; doi:10.1371/journal.pone.0244755)
Supplement: S28 Fig — Stacked barchat showing Verrucomicrobia classes, relative abundances (a) and their PCoA plot based on the Euclidean model (b). The PCoA plot revealed dissimilarities of the nucleotide composition among the treatments, except those identified in reactor 3 and 7 that clustered partially on the lower right quadrant of the plot. (PDF) [file pone.0244755.s029.pdf]

a

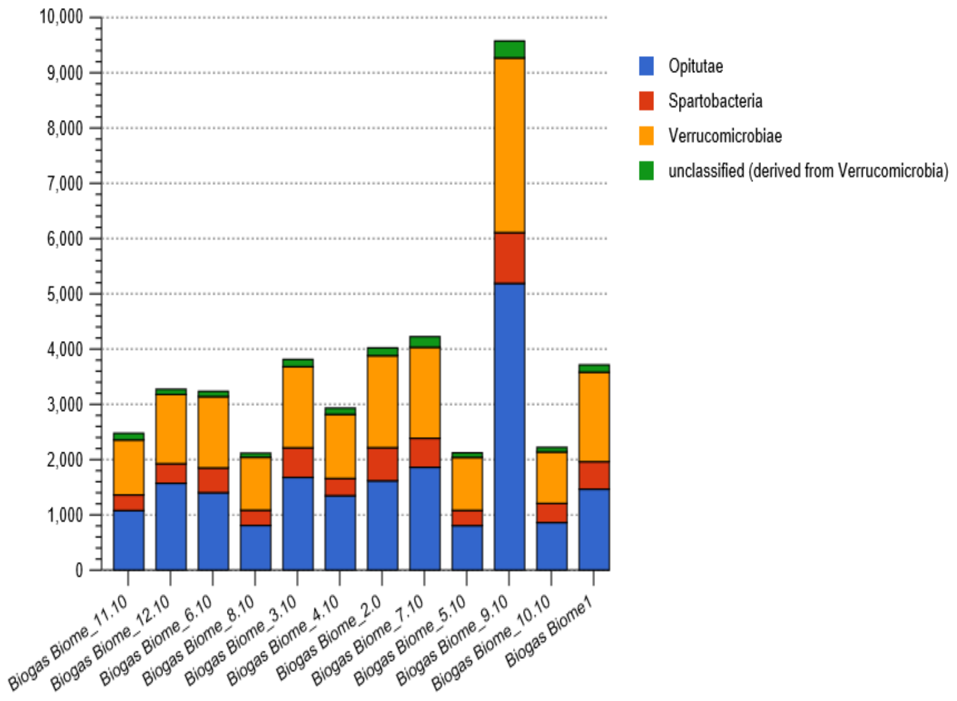

b

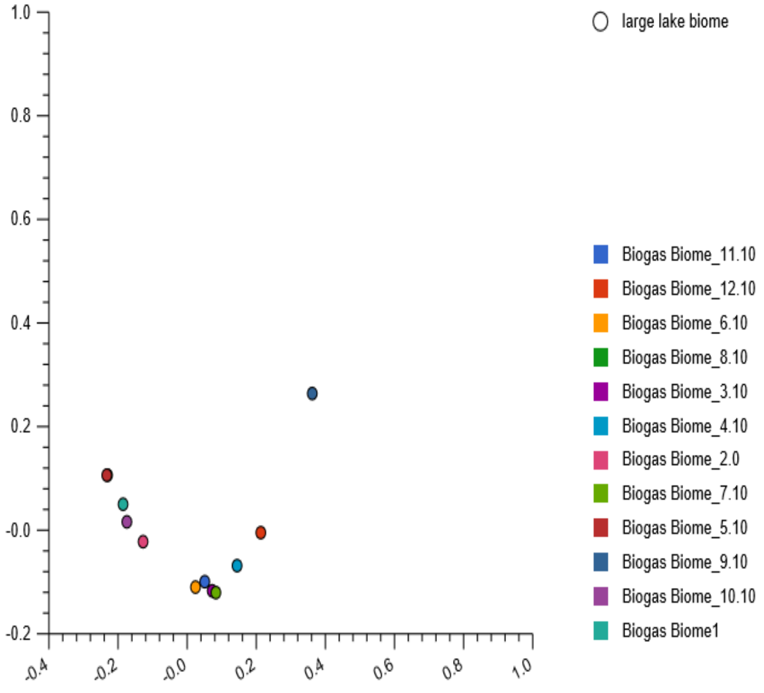

S28 Fig. Stacked barchat (a) showing *Verrucomicrobia* classes, relative abundances and their PCoA plot (b) based on the Euclidean model. The PCoA plot revealed dissimilarities of the nucleotide composition among the treatments, except those identified in reactor 3 and 7 that clustered partially on the lower right quadrant of the plot.
