# Supplementary material for: Metagenomics survey unravels diversity of biogas microbiomes with potential to enhance productivity in Kenya
Source: PLoS One. 2021 Jan 4;16(1):e0244755. doi: 10.1371/journal.pone.0244755 (PMC7781671; doi:10.1371/journal.pone.0244755)
Supplement: S29 Fig — Stacked barchat showing three Verrucomicrobia orders, relative abundances (a) and their PCoA plot based Euclidean model (b). The composition of reactor 3, 6, 7 and 11 clustered partially, on the lower left quadrant of the plot; those indentified in reactor 1 and 10 were also clustered partially, on the upper right quadrant, while the reads of reactor 5 and 8 were found to cluster on the upper right quadrant of the plot. (PDF) [file pone.0244755.s030.pdf]

a

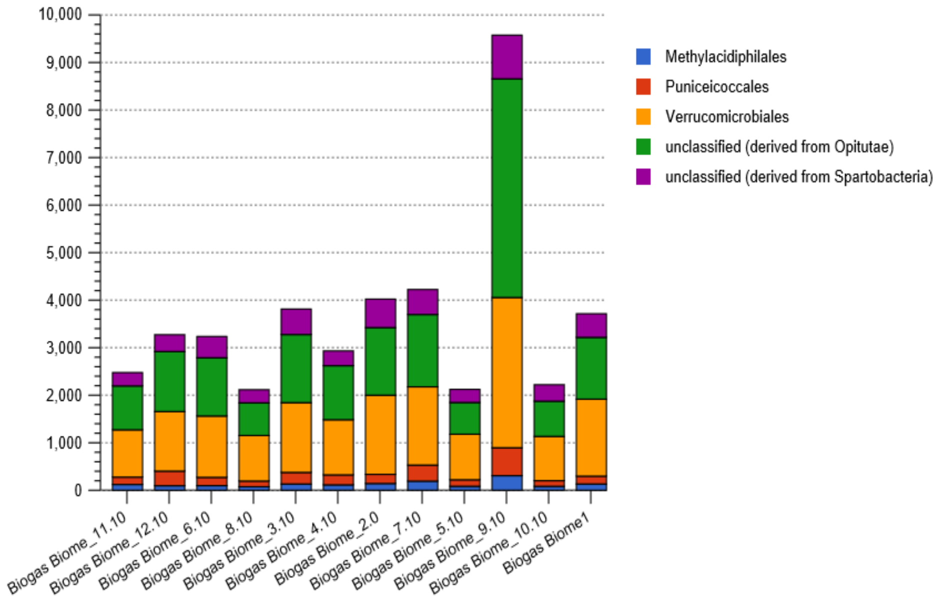

b

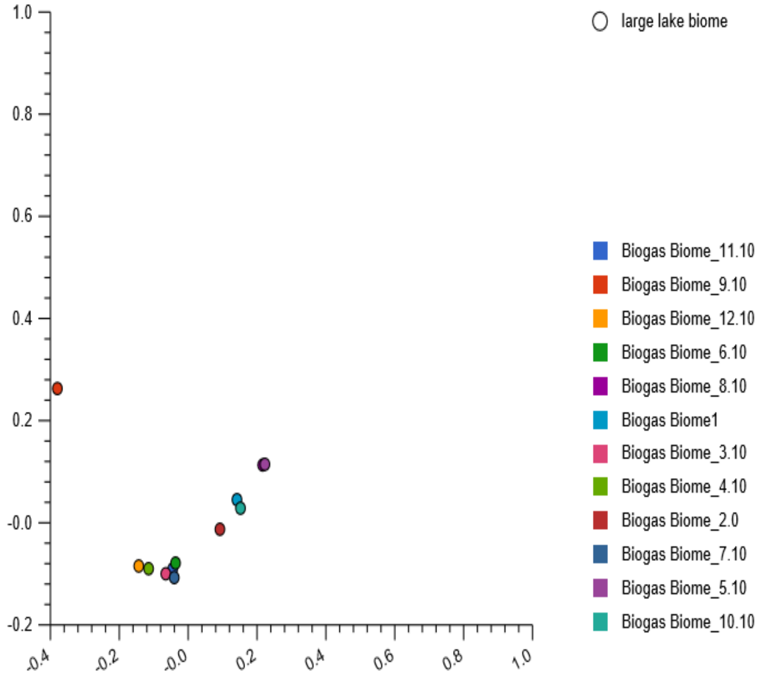

S29 Fig. Stacked barchat (a) showing three *Verrucomicrobia* orders, relative abundances and their PCoA plot (b) based Euclidean model. The composition of reactor 3, 6, 7 and 11 clustered partially, on the lower left quadrant of the plot; those identified in reactor 1 and 10 were also clustered partially, on the upper right quadrant, while the reads of reactor 5 and 8 were found to cluster on the upper right quadrant of the plot.
