# Supplementary material for: Metagenomics survey unravels diversity of biogas microbiomes with potential to enhance productivity in Kenya
Source: PLoS One. 2021 Jan 4;16(1):e0244755. doi: 10.1371/journal.pone.0244755 (PMC7781671; doi:10.1371/journal.pone.0244755)
Supplement: S30 Fig — Stacked barchat showing three Ternericutes orders, relative abundances (a) and their PCoA plot based on the Euclidean model (b). The nucleotide composition in reactor 4 and 9 and those detected in reactor 6 and 8 partially clustered in the upper right quadrant of the plot. Other treatments comprised dissimilar nucleotide composition. (PDF) [file pone.0244755.s031.pdf]

a

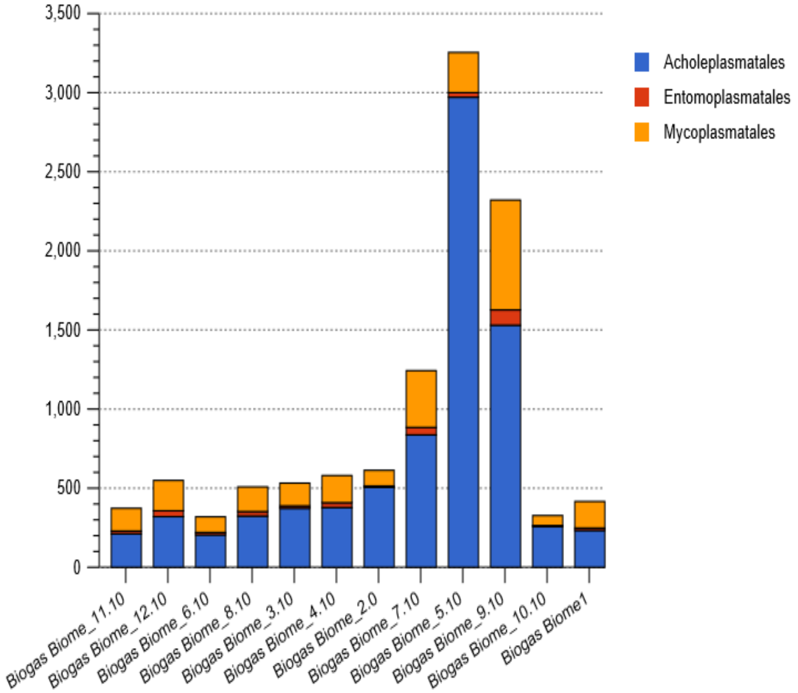

b

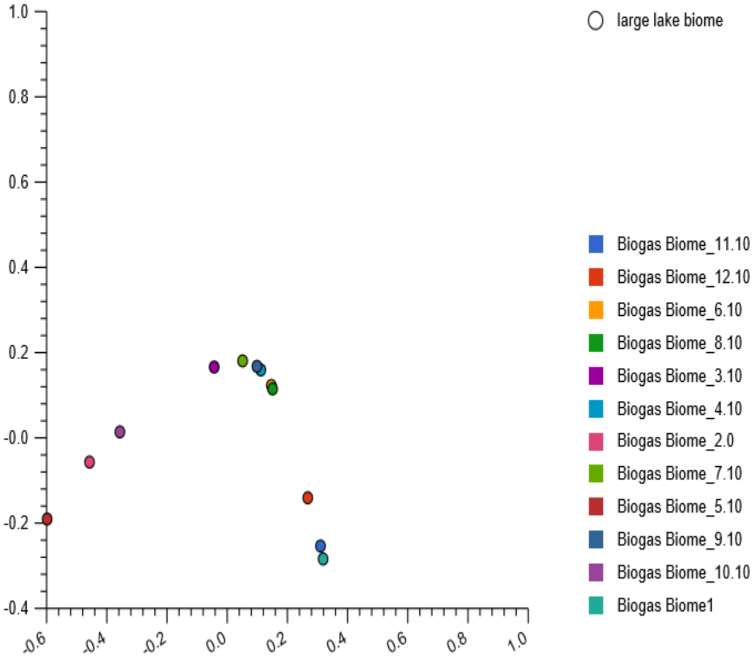

S30 Fig. Stacked barchat (a) showing three *Terneicutes* orders, relative abundances and their PCoA plot (b) based on the Euclidean model. The nucleotide composition in reactor 4 and 9 and those detected in reactor 6 and 8 partially clustered in the upper right quadrant of the plot. Other treatments comprised dissimilar nucleotide composition.
