# Supplementary material for: Metagenomics survey unravels diversity of biogas microbiomes with potential to enhance productivity in Kenya
Source: PLoS One. 2021 Jan 4;16(1):e0244755. doi: 10.1371/journal.pone.0244755 (PMC7781671; doi:10.1371/journal.pone.0244755)
Supplement: S31 Fig — The stacked barchart showing the four Archaea phyla, relative abundances (a) and their PCoA plot based on the Euclidean model (b). The compositions of reactor 4 and 12 clustered, on the lower left quadrant of the plot; while those of reactor 3 and 6 and reactor 7 and 9 clustered partially; on the lower right quadrant and upper left quadrant of the plot. Other treatments were distinctively dissimilar, positioned within the plot. (PDF) [file pone.0244755.s032.pdf]

a

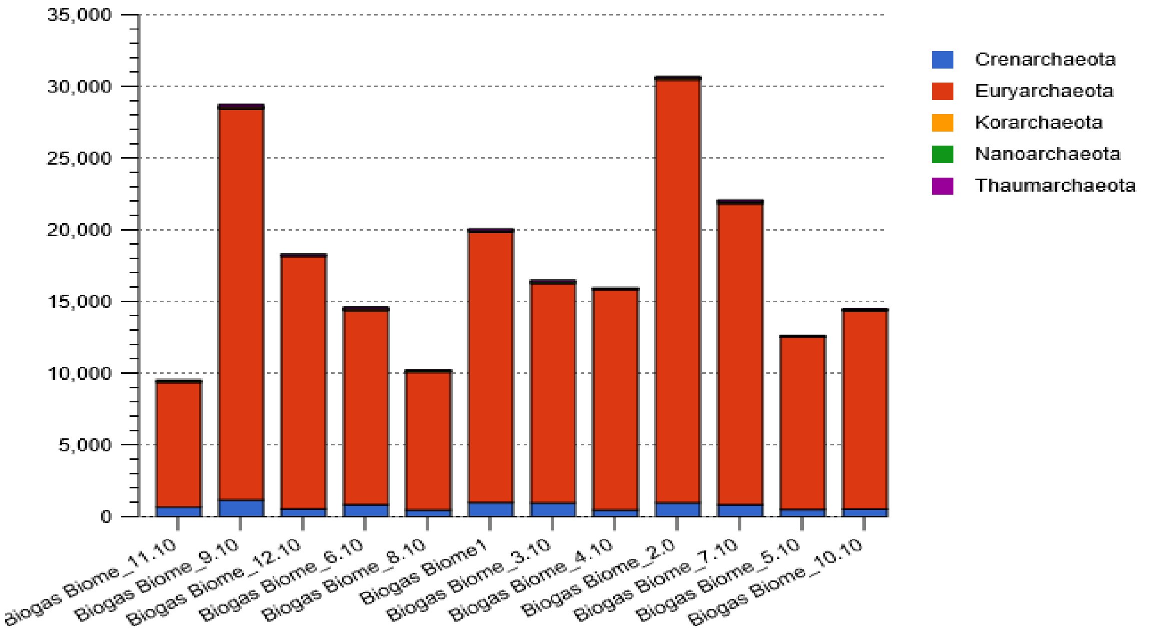

b

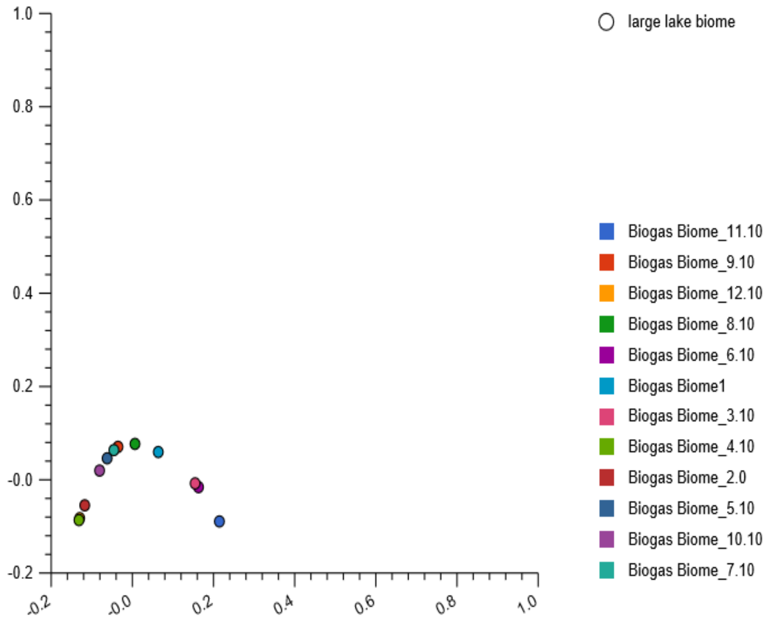

S31 Fig. The stacked barchart (a) showing the four Archaeal phyla, relative abundances and their PCoA plot (b) based on the Euclidean model. The compositions of reactor 4 and 12 clustered, on the lower left quadrant of the plot; while those of reactor 3 and 6 and reactor 7 and 9 clustered partially; on the lower right quadrant and upper left quadrant of the plot. Other treatments were distinctively dissimilar, positioned within the plot.
