# Supplementary material for: Metagenomics survey unravels diversity of biogas microbiomes with potential to enhance productivity in Kenya
Source: PLoS One. 2021 Jan 4;16(1):e0244755. doi: 10.1371/journal.pone.0244755 (PMC7781671; doi:10.1371/journal.pone.0244755)
Supplement: S32 Fig — Stacked barchat showing nine Archaea classes, and the affiliates of the unclassified reads, relative abundances (a) and their PCoA plot based on the Euclidean model (b). The nucleotide compositions in the respective treatments were distinctively dissimilar. (PDF) [file pone.0244755.s033.pdf]

a

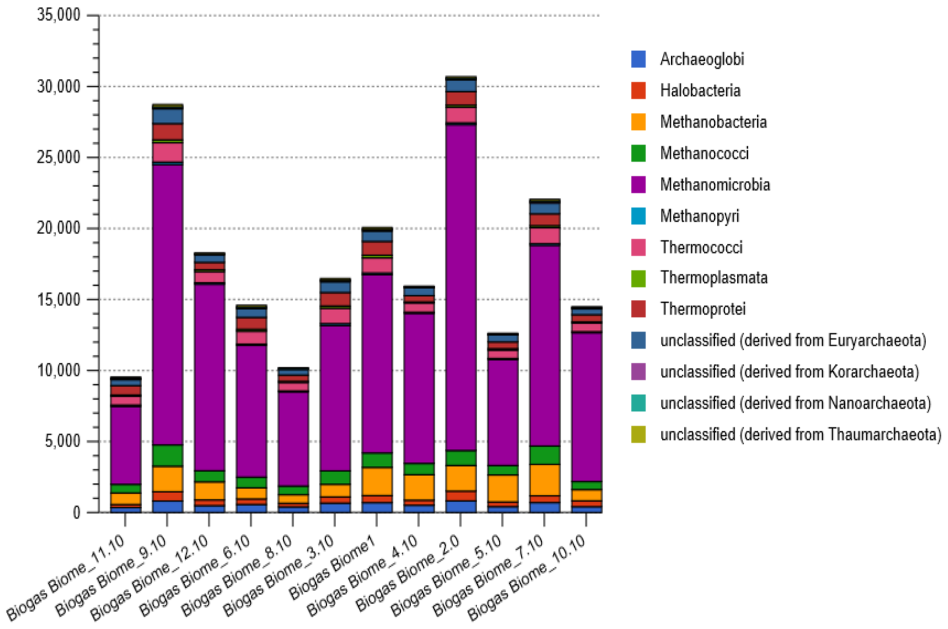

b

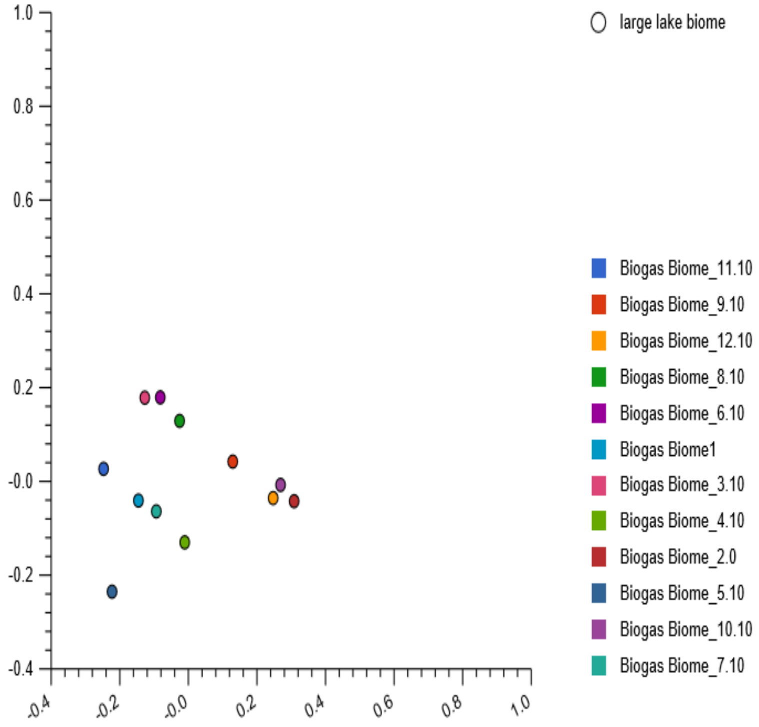

S32 Fig. Stacked barchat (a) showing nine archaea classes, and the affiliates of the unclassified reads, relative abundances and their PCoA plot (b) based on the Euclidean model. The nucleotide compositions in the respective treatments were distinctively dissimilar.
