# Supplementary material for: Metagenomics survey unravels diversity of biogas microbiomes with potential to enhance productivity in Kenya
Source: PLoS One. 2021 Jan 4;16(1):e0244755. doi: 10.1371/journal.pone.0244755 (PMC7781671; doi:10.1371/journal.pone.0244755)
Supplement: S33 Fig — Stacked barchat showing sixteen Archaea orders, relative abundances (a) and their PCoA plot based on the Euclidean model (b). The nucleotide of reactor 3 and 7, were clustered partially on the lower left quadrant of the plot. However, the compositions of the other ten treatments were distinctively dissimilar, all distributed in the plot. (PDF) [file pone.0244755.s034.pdf]

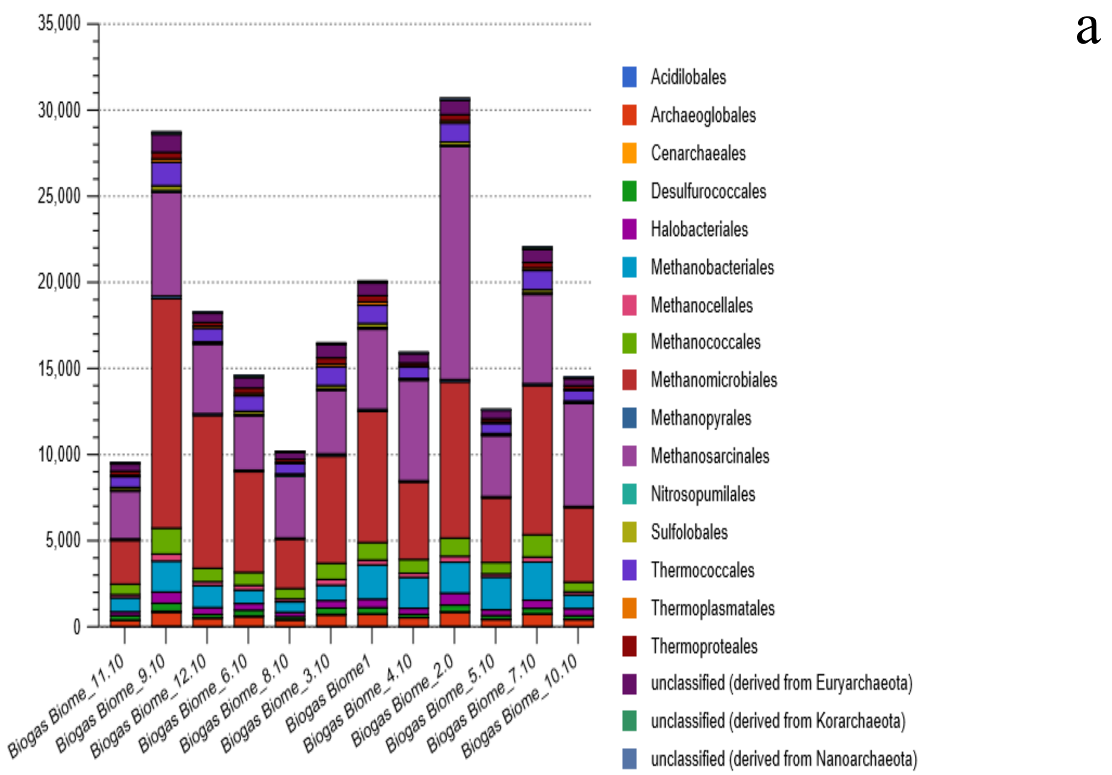

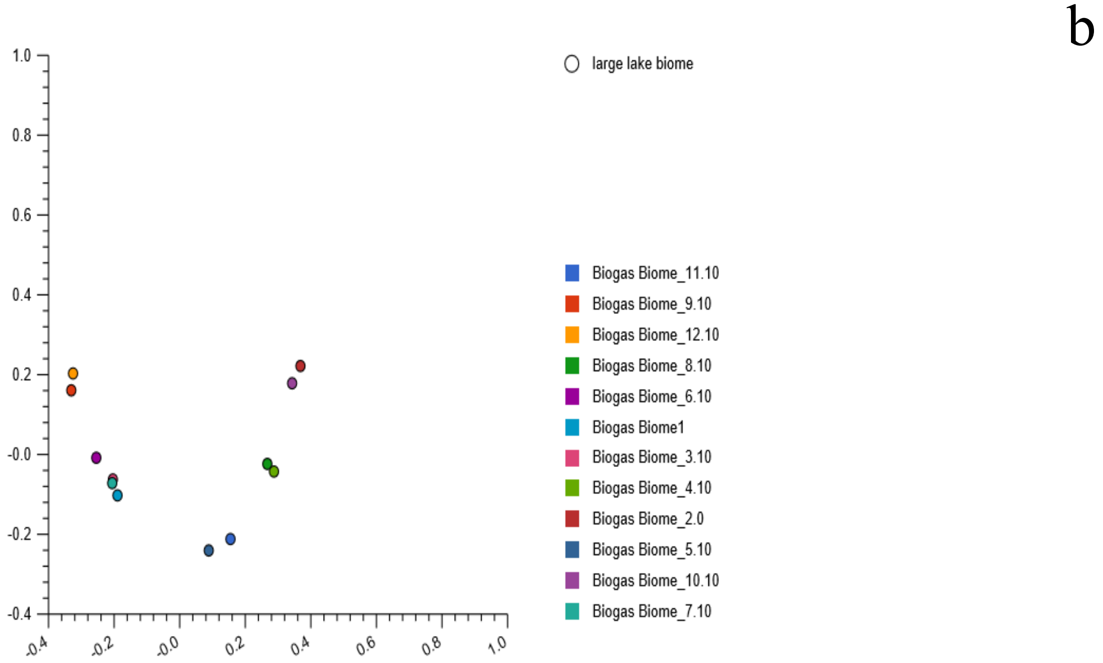

S33 Fig. Stacked barchat showing sixteen Archaeal orders, relative abundances and their PCoA plot based on the Euclidean model. The nucleotide of reactor 3 and 7, were clustered partially on the lower left quadrant of the plot. However, the compositions of the other ten treatments were distinctively dissimilar, all distributed in the plot.
