# Supplementary material for: Metagenomics survey unravels diversity of biogas microbiomes with potential to enhance productivity in Kenya
Source: PLoS One. 2021 Jan 4;16(1):e0244755. doi: 10.1371/journal.pone.0244755 (PMC7781671; doi:10.1371/journal.pone.0244755)
Supplement: S34 Fig — The stacked barchat showing the eight Eurychaeota classes, the relative abundances (a) and their PCoA plots revealing dissimilarities of the nucleotide composition based on the Euclidean model (b). The nucleotide composition of reactor 1 and 7, were positioned on the upper left quadrant of the plot while the composition of reactor 6 and 8, were positioned on the lower left quadrant, near the y-axis were in close proximity. (PDF) [file pone.0244755.s035.pdf]

a

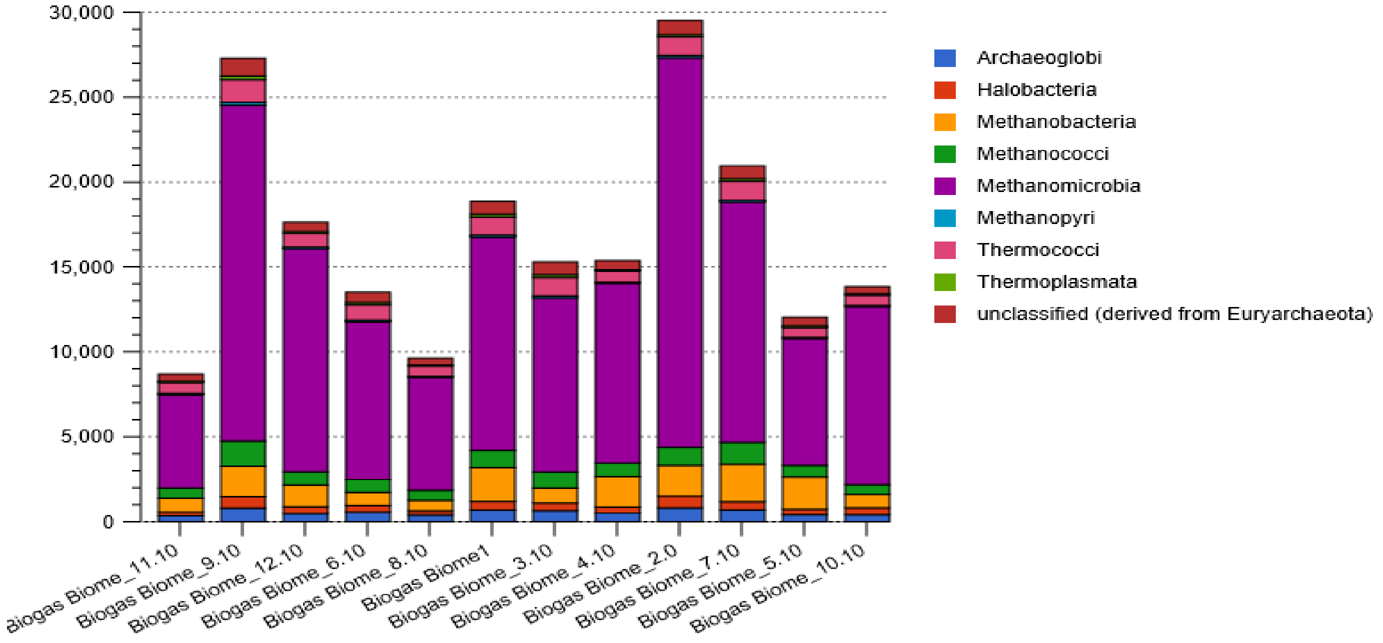

b

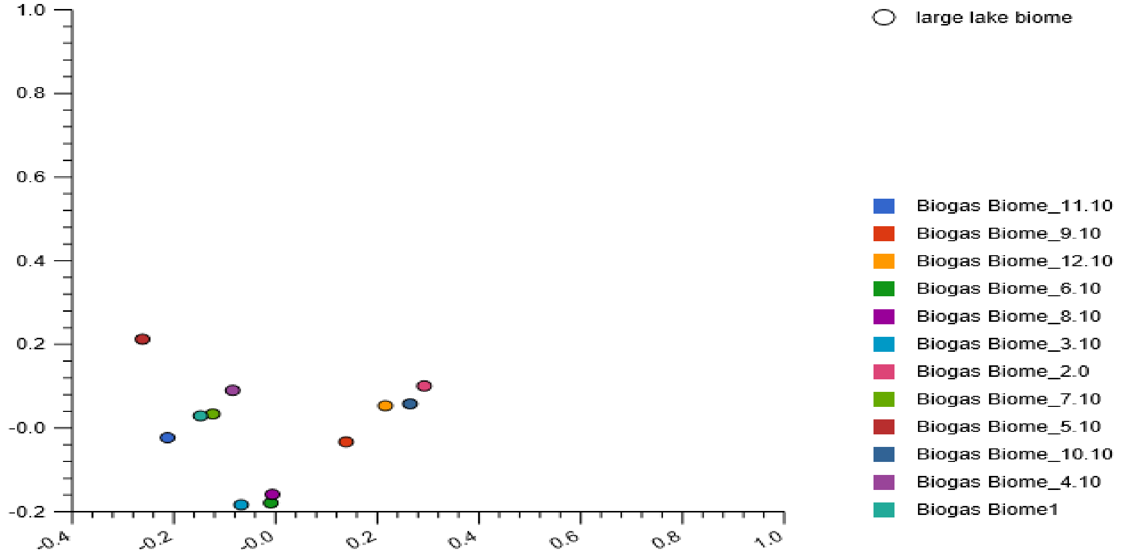

S34 Fig. The stacked barchat (a) showing the eight *Eurychaeota* classes, the proportion of relative abundances and their PCoA plots (b) revealing dissimilarities of the nucleotide composition among the twelve studied treatments. The nucleotide composition of reactor 1 and 7, were positioned on the upper left quadrant of the plot while the composition of reactor 6 and 8, were positioned on the lower left quadrant, near the y-axis were in close proximity.
