# Supplementary material for: Metagenomics survey unravels diversity of biogas microbiomes with potential to enhance productivity in Kenya
Source: PLoS One. 2021 Jan 4;16(1):e0244755. doi: 10.1371/journal.pone.0244755 (PMC7781671; doi:10.1371/journal.pone.0244755)
Supplement: S35 Fig — Stacked barchat showing ten Euryarchaeota orders, relative abundances (a) and their PCoA plot based Euclidean model (b). Their nucleotide compositions were found toreveal dissimilarity, all distributed within the four quadrants. (PDF) [file pone.0244755.s036.pdf]

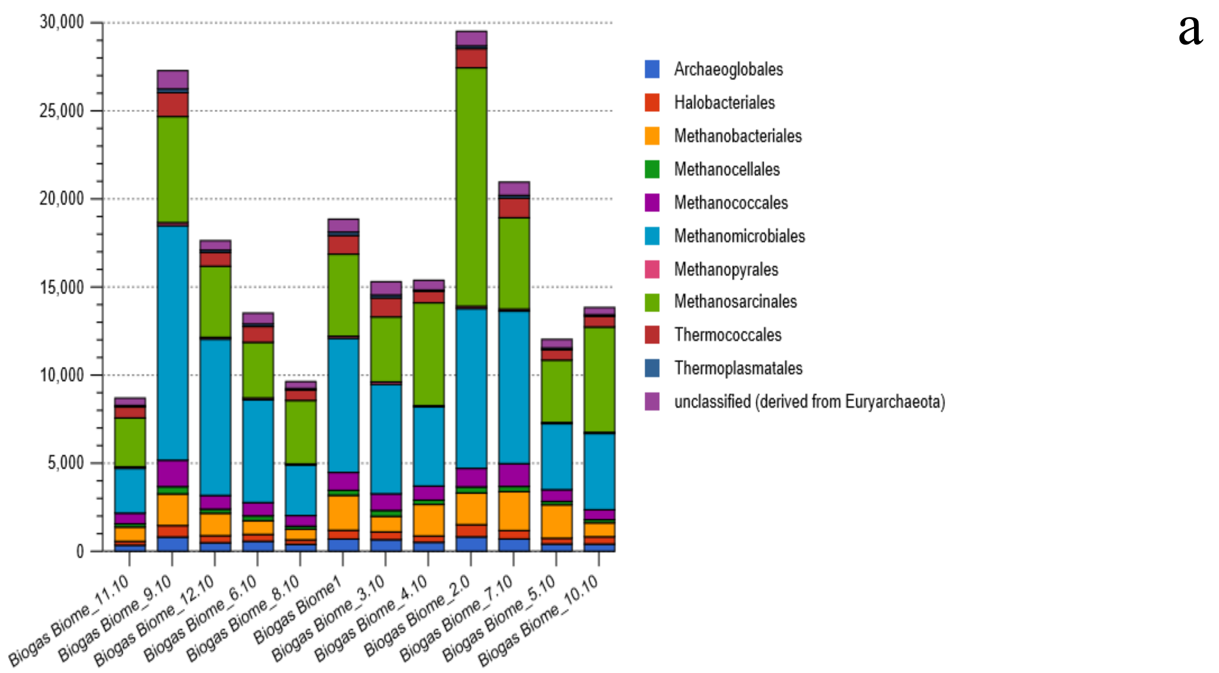

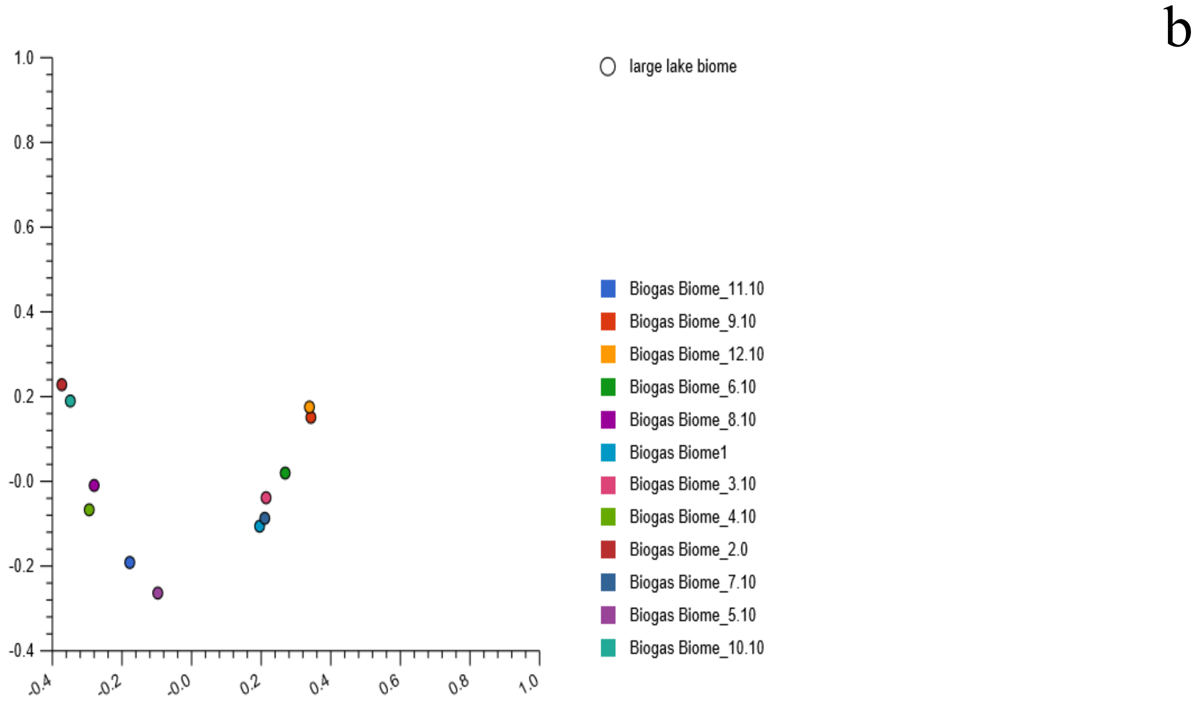

S35 Fig. Stacked barchat (a) showing ten *Euryarchaeota* orders, relative abundances and their PCoA plot (b) based Euclidean model. Their nucleotide compositions were found toreveal dissimilarity, all distributed within the four quadrants. The nucleotide composition of reactor 1 and 7, were positioned on the upper left quadrant of the plot while the composition of reactor 6 and 8, were positioned on the lower left quadrant, near the y-axis were in close proximity.
