# Supplementary material for: Metagenomics survey unravels diversity of biogas microbiomes with potential to enhance productivity in Kenya
Source: PLoS One. 2021 Jan 4;16(1):e0244755. doi: 10.1371/journal.pone.0244755 (PMC7781671; doi:10.1371/journal.pone.0244755)
Supplement: S36 Fig — The stacked barchat showing three Methanomicrobia orders, the relative abundances (a) and their PCoA plot based on the Euclidean model (b). The PCoA plot revealed partial clustering of reactor 1 and 7 nucleotide composition on the upper left quadrant of the plot. Further the composition of reactor 2 and 10 and those of reactor 4 and 8 were found to be in close proximity on the lower right quadrant of the plot. (PDF) [file pone.0244755.s037.pdf]

a

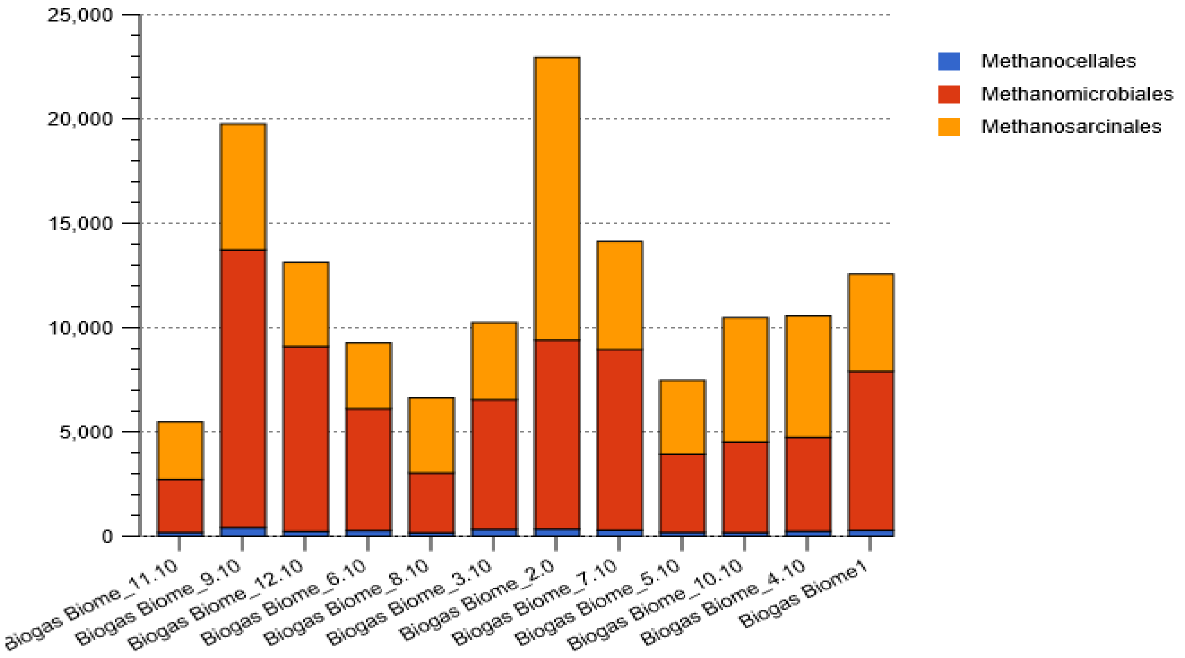

b

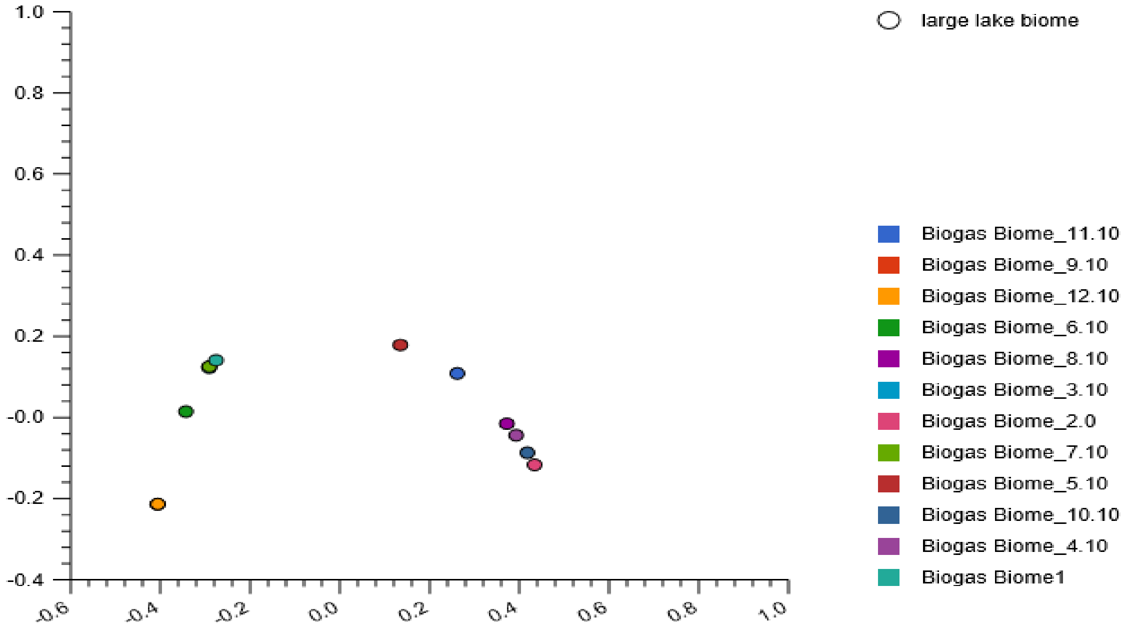

S36 Fig. The stacked barchat (a) showing three *Methanomicrobia* orders, the proportion of relative abundances and their PCoA plot (b) based on the Euclidean model. The PCoA plot revealed partial clustering of reactor 1 and 7 nucleotide composition on the upper left quadrant of the plot. Further the composition of reactor 2 and 10 and those of reactor 4 and 8 were found to be in close proximity on the lower right quadrant of the plot.
