# Supplementary material for: Metagenomics survey unravels diversity of biogas microbiomes with potential to enhance productivity in Kenya
Source: PLoS One. 2021 Jan 4;16(1):e0244755. doi: 10.1371/journal.pone.0244755 (PMC7781671; doi:10.1371/journal.pone.0244755)
Supplement: S37 Fig — Stacked barchat showing four Thermoprotei orders, relative abundances and their PCoA plot based on the Euclidean model (b). However, the nucleotide composition of reactor 3 and 8 were closely positioned in the lower left quadrant of the plot. (PDF) [file pone.0244755.s038.pdf]

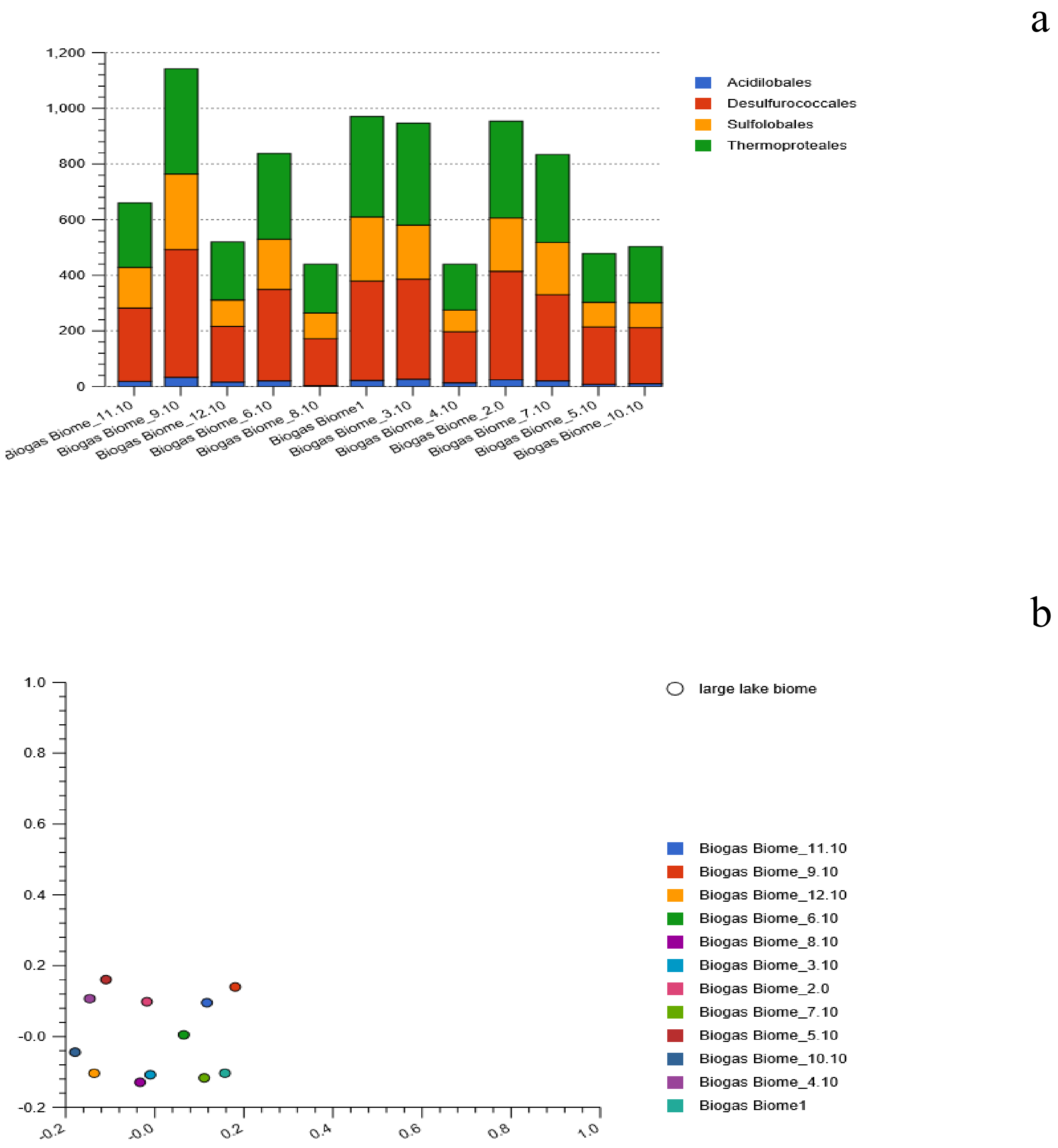

S37 Fig. Stacked barchat (a) showing four Thermoprotei orders and their PCoA plot (b) based on the Euclidean model. The plot reveal nucleotide composition dissimilarities among the twelve treatments. The PCoA plot revealed partial clustering of reactor 1 and 7 nucleotide composition on the upper left quadrant of the plot. Further the composition of reactor 2 and 10 and those of reactor 4 and 8 were found to be in close proximity on the lower right quadrant of the plot.
