# Supplementary material for: Metagenomics survey unravels diversity of biogas microbiomes with potential to enhance productivity in Kenya
Source: PLoS One. 2021 Jan 4;16(1):e0244755. doi: 10.1371/journal.pone.0244755 (PMC7781671; doi:10.1371/journal.pone.0244755)
Supplement: S39 Fig — Stacked barchat showing five fungal phyla, the relative abundances (a) and their PCoA plots based on Euclidean model (b). The plot revealed dissimilarities of the nucleotide composition among the twelve studied treatments and were distributed within the four plot quadrant. (PDF) [file pone.0244755.s040.pdf]

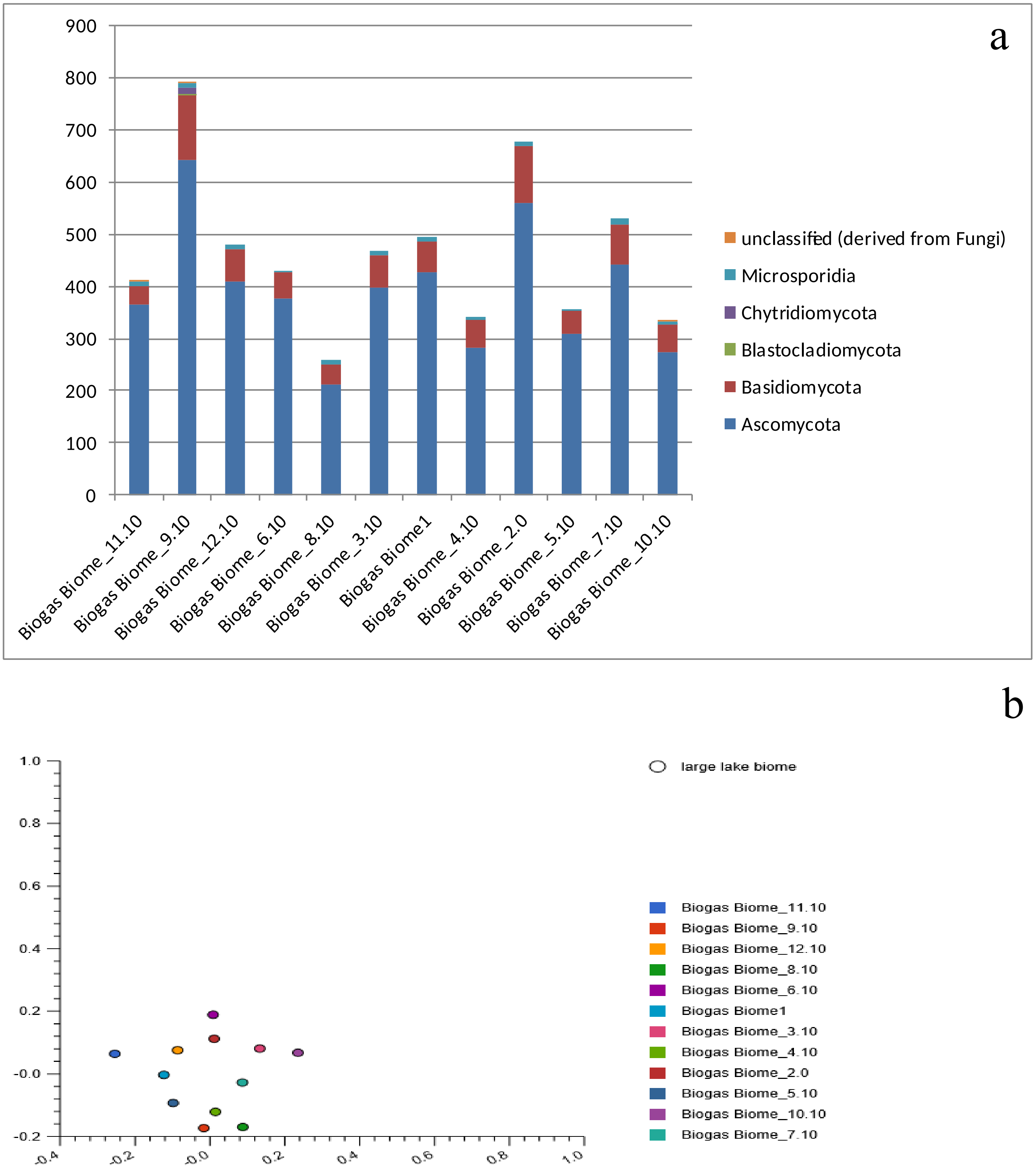

S39 Fig. Stacked barchat (a) showing five Fungal phyla, proportion of the relative abundances and their PCoA plots (b) based on Euclidean model. The plot revealed dissimilarities of the nucleotide composition among the twelve studied treatments and were distributed within the four plot quadrant.
