# Supplementary material for: Metagenomics survey unravels diversity of biogas microbiomes with potential to enhance productivity in Kenya
Source: PLoS One. 2021 Jan 4;16(1):e0244755. doi: 10.1371/journal.pone.0244755 (PMC7781671; doi:10.1371/journal.pone.0244755)
Supplement: S40 Fig — Stacked barchat showing thirteen fungal classes, relative abundances (a) and their PCoA plot based on the Euclidean model (b). The PCoA plot revealed nucleotide composition dissimilarities among the twelve studied treatments. (PDF) [file pone.0244755.s041.pdf]

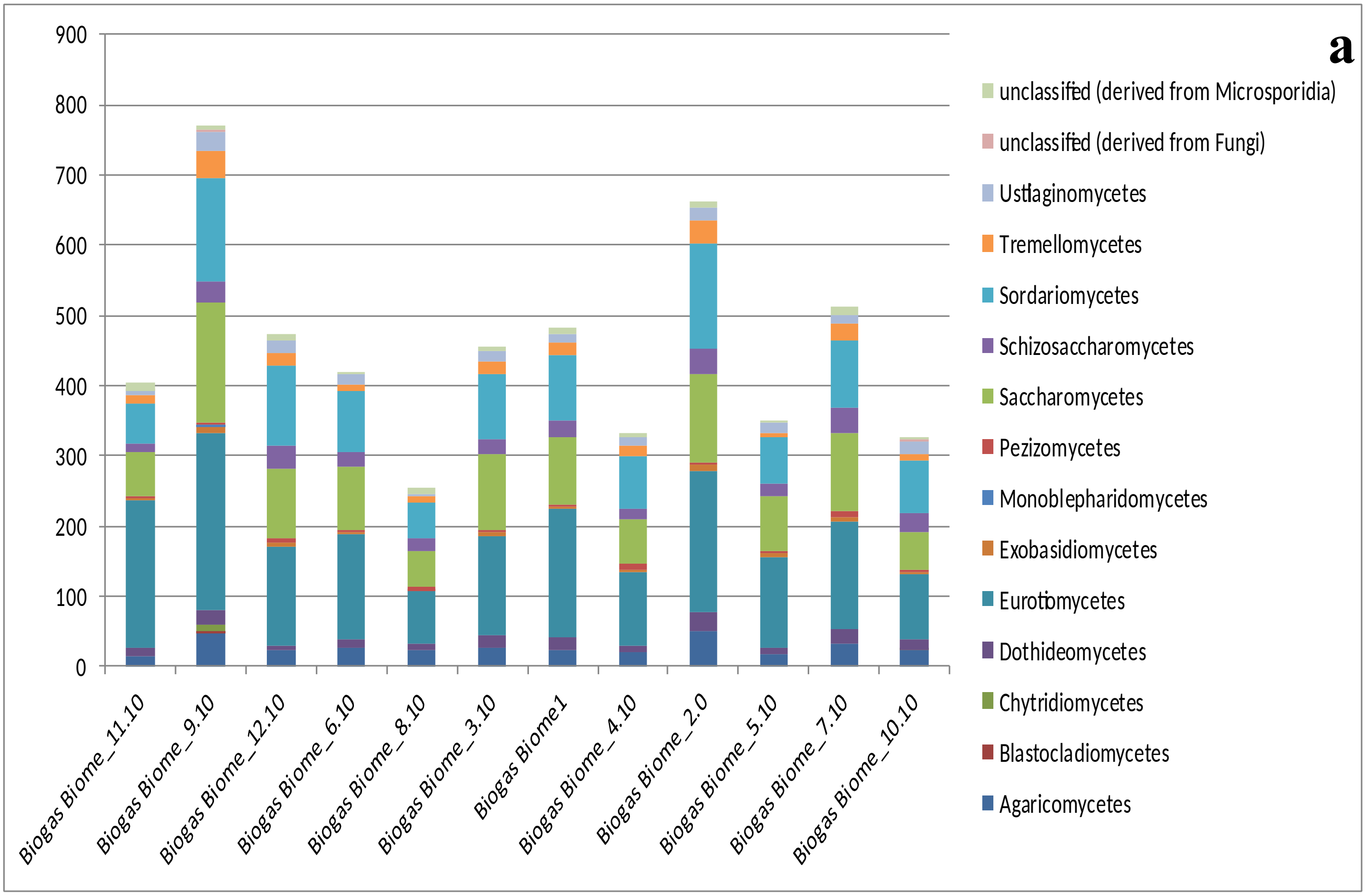

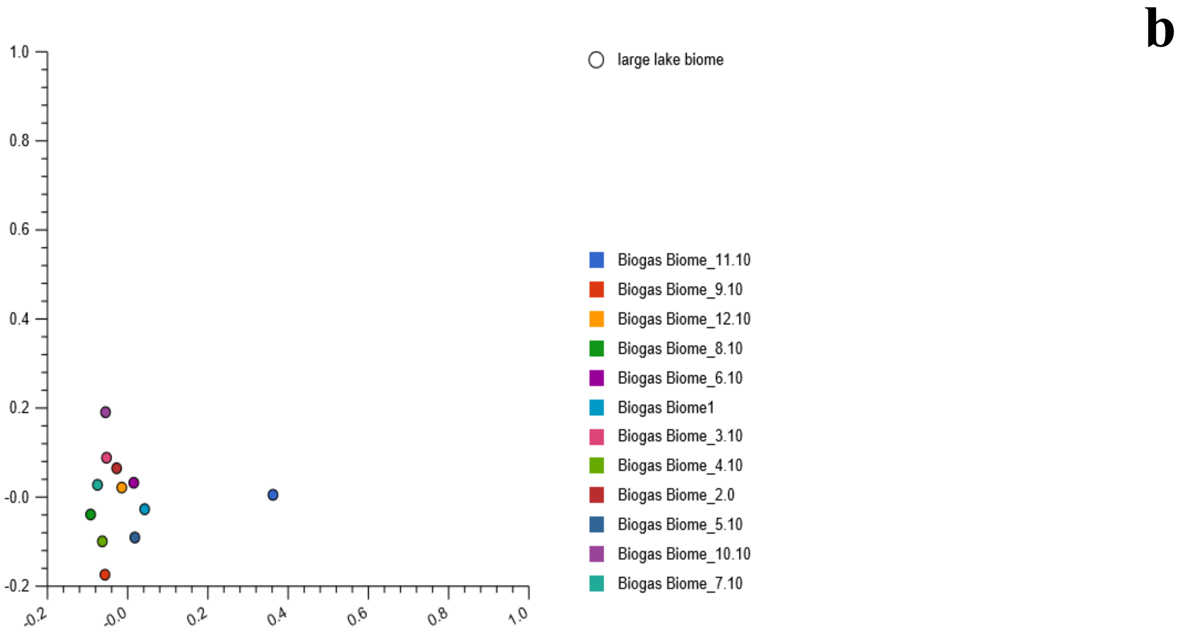

SFig. 40: Stacked barchat (a) showing thirteen eukaryotes classes, relative abundances and their PCoA plot (b) based on the Euclidean model. The plot revealed partial clustering of the nucleotide composition of reactor 6 and 9, positioned on the lower left quadrant of the plot, reactor 7 and 10, located near the y-axis, and reactor 2 and 11 located on the upper right quadrant of the plot. The nucleotide compositions of reactor 3 were in close proximity to those identified in reactor 7, positioned on the lower right quadrant of the plot. However, the other treatments' nucleotide compositions were found to be dissimilar.
