# Supplementary material for: Metagenomics survey unravels diversity of biogas microbiomes with potential to enhance productivity in Kenya
Source: PLoS One. 2021 Jan 4;16(1):e0244755. doi: 10.1371/journal.pone.0244755 (PMC7781671; doi:10.1371/journal.pone.0244755)
Supplement: S41 Fig — Stacked barchat showing 23 fungal orders, relative abundances (a) and their PCoA plot based on the Euclidean model (b). The nucleotide compositions of reactor 2 and 10, were positioned on the lower left quadrant; reactor 3 and 6, upper left quadrant; reactor 4 and 9, upper right quadrant; and reactor 8 and 11, on the lower right quadrant of the plot. All clustering partially in their respective quadrant. (PDF) [file pone.0244755.s042.pdf]

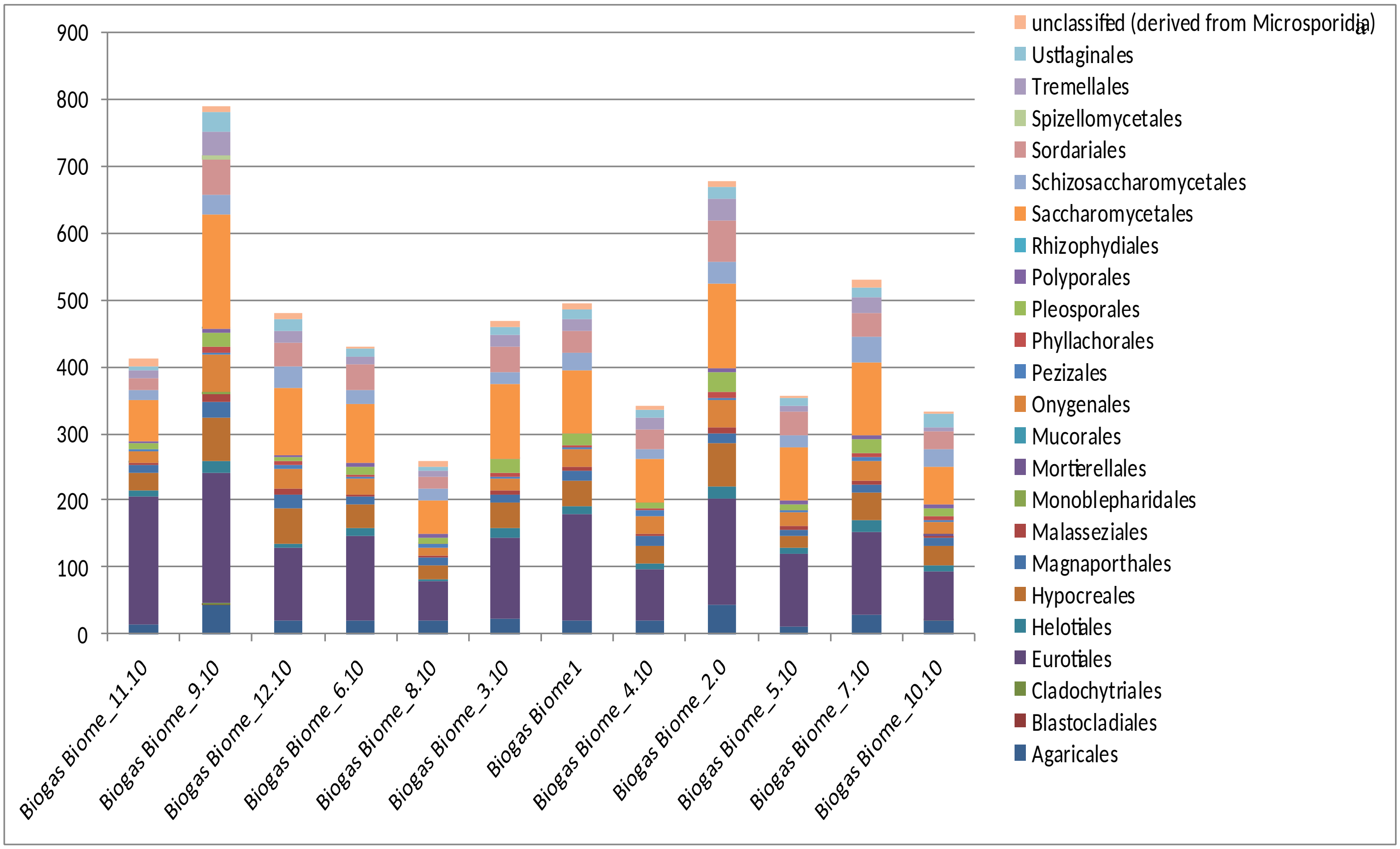

b

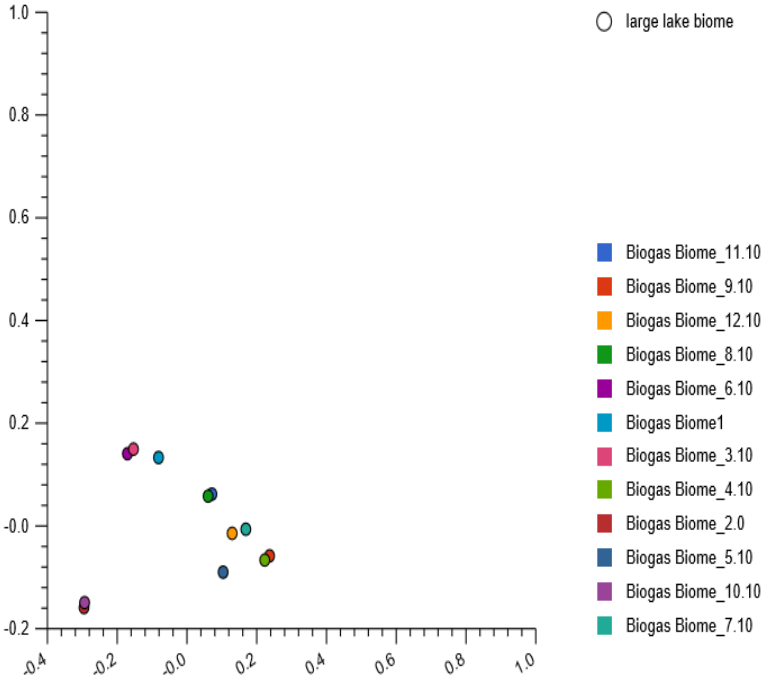

S41 Fig. Stacked barchat (a) showing 23 fungal orders, relative abundances and their PCoA plot (b) based on the Euclidean model. The nucleotide compositions of reactor 2 and 10, were positioned on the lower left quadrant; reactor 3 and 6, upper left quadrant; reactor 4 and 9, upper right quadrant; and reactor 8 and 11, on the lower right quadrant of the plot. All clustering partially in their respective quadrant.
