# Supplementary material for: Metagenomics survey unravels diversity of biogas microbiomes with potential to enhance productivity in Kenya
Source: PLoS One. 2021 Jan 4;16(1):e0244755. doi: 10.1371/journal.pone.0244755 (PMC7781671; doi:10.1371/journal.pone.0244755)
Supplement: S42 Fig — The stacked barchat showing the five Ascomycota classes, relative abaundances (a) and their PCoA plots based on the Euclidean model (b). The PCoA plots revealed dissimilarity of the nucleotide composition among the twelve treatments. However, the composition of reactor 11 was positioned singly on the lower left quadrant of the plot while those of reactor 10 were located along the y-axis (Negative PCoA 1 and Negative PCoA 2). (PDF) [file pone.0244755.s043.pdf]

a

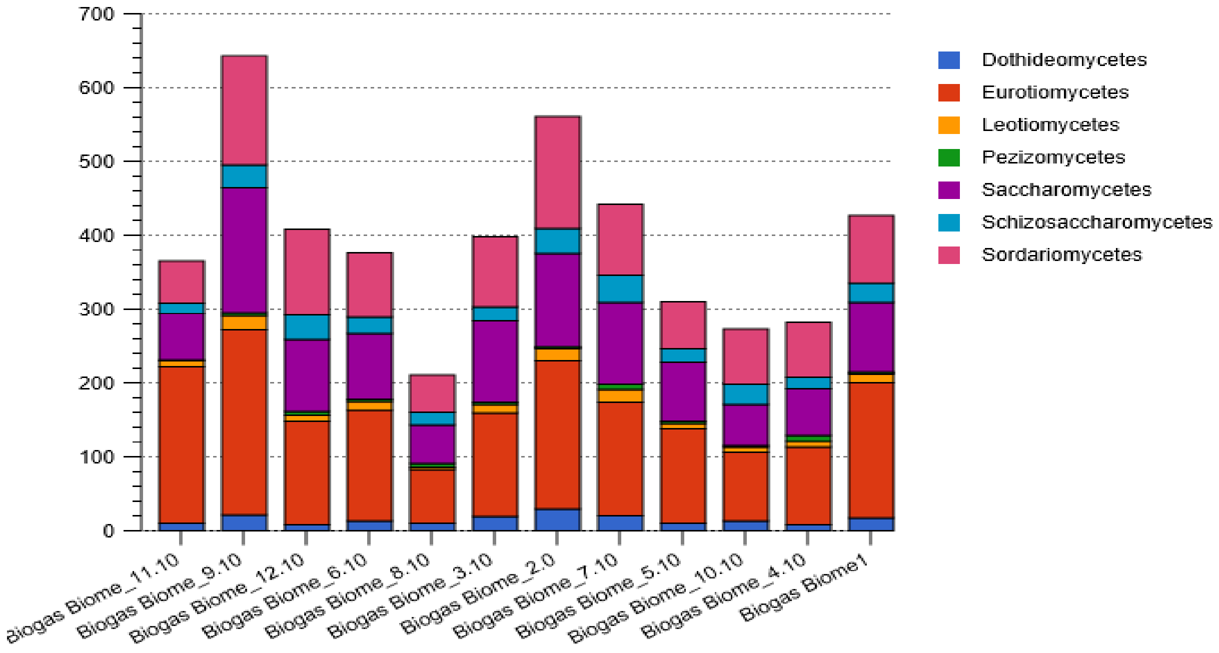

b

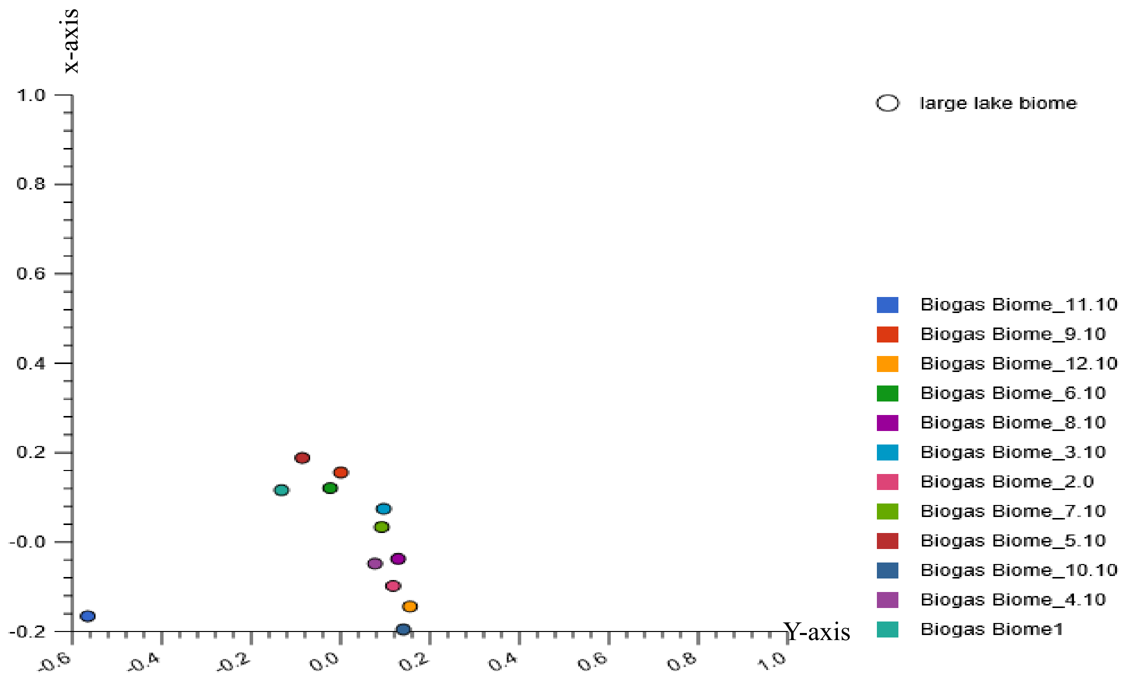

S42 Fig. The stacked barchat (a) showing the five Ascomycota classes, relative abaundances and their PCoA plots (b), based on the Euclidean model at the class level. The PCoA plots revealed dissimilarity of the nucleotide composition among the twelve treatments. However, the composition of reactor 11 was positioned singly on the lower left quadrant of the plot while those of reactor 10 were located along the y-axis (Negative PCoA 1 and Negative PCoA 2).
