# Supplementary material for: Metagenomics survey unravels diversity of biogas microbiomes with potential to enhance productivity in Kenya
Source: PLoS One. 2021 Jan 4;16(1):e0244755. doi: 10.1371/journal.pone.0244755 (PMC7781671; doi:10.1371/journal.pone.0244755)
Supplement: S43 Fig — The stacked barchat revealing eleven Ascomycota orders, relative abundances (a) and their PCoA plot based on the Euclidean model (b). The model revealed partial clustering of the identified nucleotide compositions of reactor 7 and 8 while those identified in reactor 2 and 8 and reactor 2 and 4 were found to reveal close proximity, located on the upper right quadrant of the plot. The Ascomycota’s nucleotide compositions of reactor 11 were singly positioned on the upper left quadrant of the plot. (PDF) [file pone.0244755.s044.pdf]

a

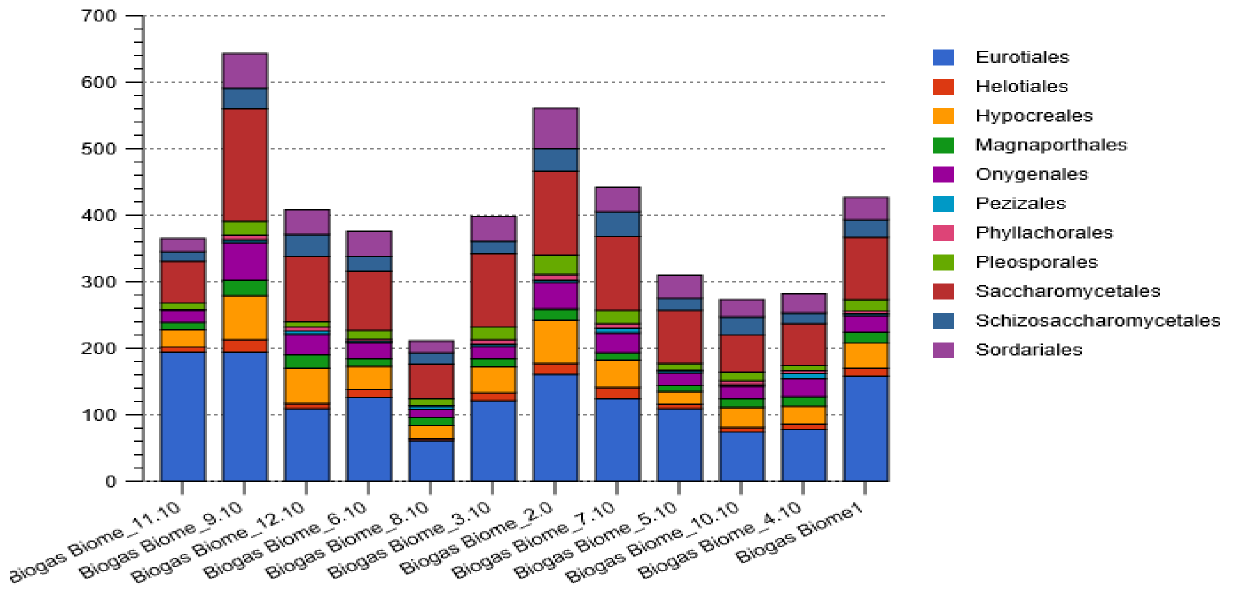

b

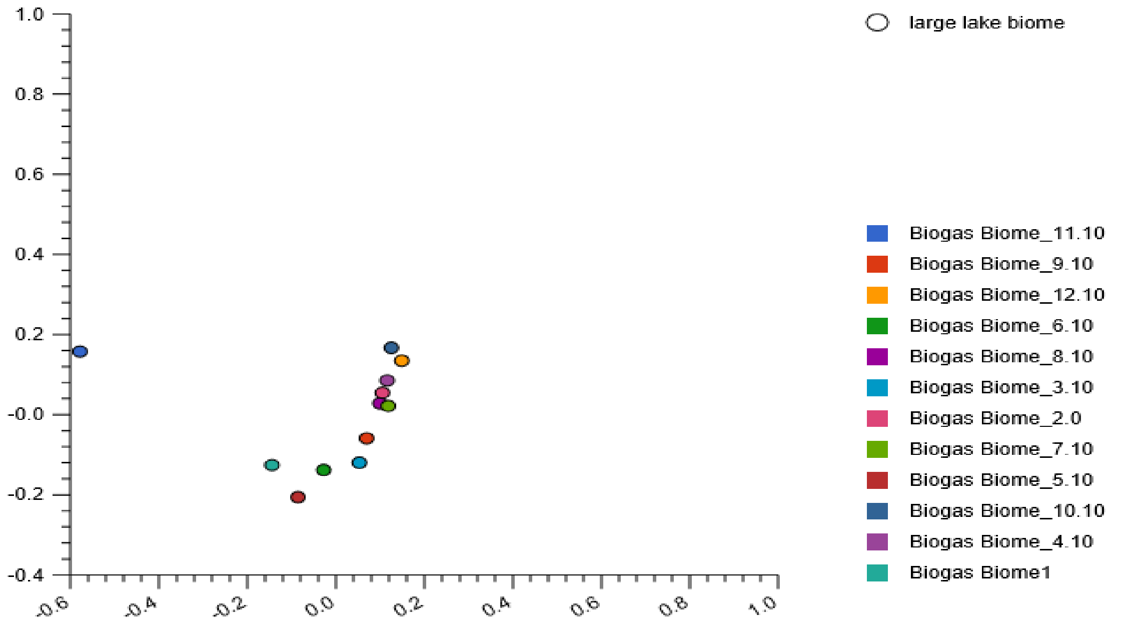

S43 Fig. The stacked barchat (a) revealing eleven Ascomycota orders, proportions of relative abundances and their PCoA plot (b); based on the Euclidean model revealed dissimilarities among the twelve treatments. The PCoA plots revealed partial clustering of the identified nucleotide reads in reactor 1 and 10 while other treatments revealed dissimilarities of the nucleotide composition.
