# Supplementary material for: Metagenomics survey unravels diversity of biogas microbiomes with potential to enhance productivity in Kenya
Source: PLoS One. 2021 Jan 4;16(1):e0244755. doi: 10.1371/journal.pone.0244755 (PMC7781671; doi:10.1371/journal.pone.0244755)
Supplement: S44 Fig — Stacked barchat showing three Sordariomycetes orders, relative abundances (a) and their PCoA plot based on the Euclidean model (b). The PCoA plots revealed partial clustering of the identified nucleotide reads in reactor 1 and 10 while other treatments revealed dissimilarities of the nucleotide composition. (PDF) [file pone.0244755.s045.pdf]

a

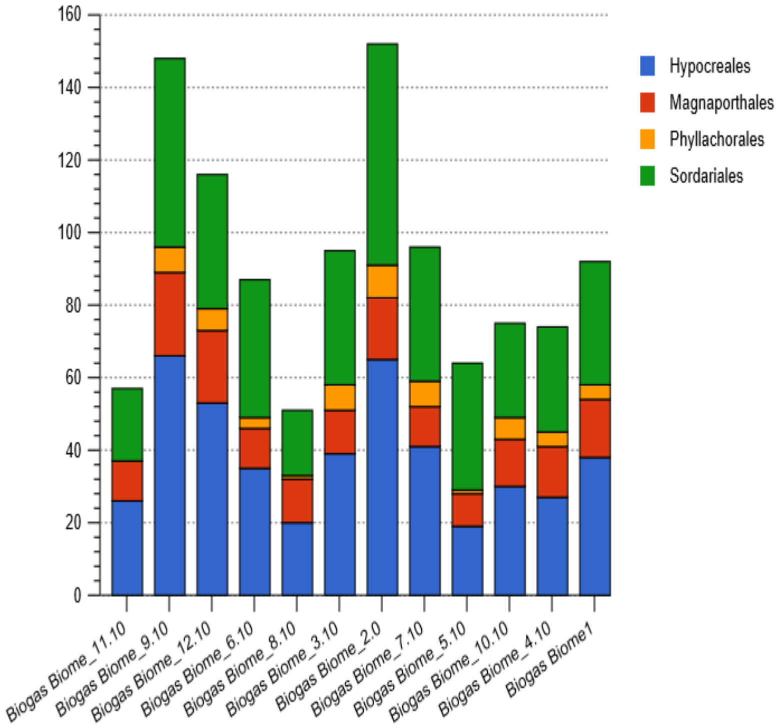

b

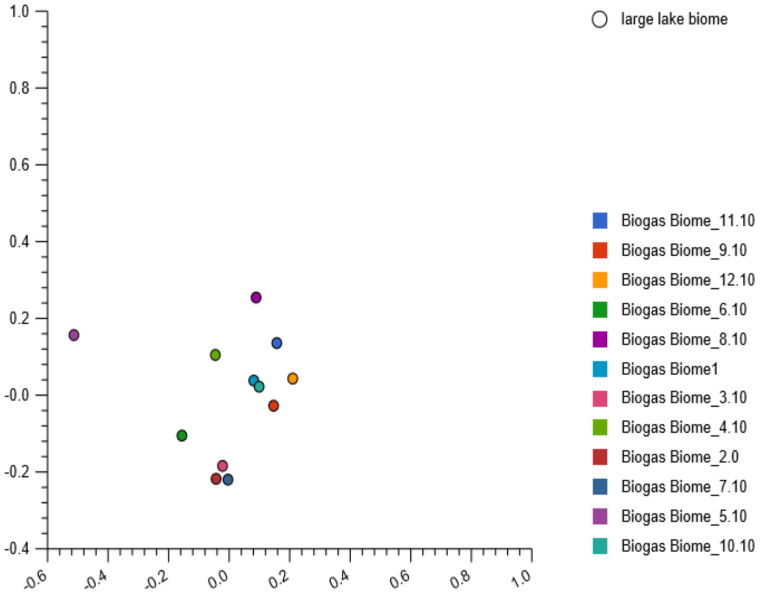

SFig. 44: Stacked barchat showing three Sordariomycetes orders, relative abundances and their PCoA plot based on the Euclidean model. The PCoA plots revealed partial clustering of the identified nucleotide reads in reactor 1 and 10 while other treatments revealed dissimilarities of the nucleotide composition.
