# Supplementary material for: Metagenomics survey unravels diversity of biogas microbiomes with potential to enhance productivity in Kenya
Source: PLoS One. 2021 Jan 4;16(1):e0244755. doi: 10.1371/journal.pone.0244755 (PMC7781671; doi:10.1371/journal.pone.0244755)
Supplement: S45 Fig — Stacked barchat showing the two Eurotiomycetes orders, the relative abundances (a) and their PCoA plot based on the Euclidean model (b). The nucleotide composition of reactor 1 and 3 clustered on the x-axis (positive PCoA 1 and Positive PCoA2); those of reactor 2 and 7, were positioned on the lower left quadrant while the communities of reactor 6 and 8, were found in close proximity on the lower right quadrant of the plot. However, the nucleotide compositions of reactor 11 were singly located on the upper right quadrant of the plot. (PDF) [file pone.0244755.s046.pdf]

a

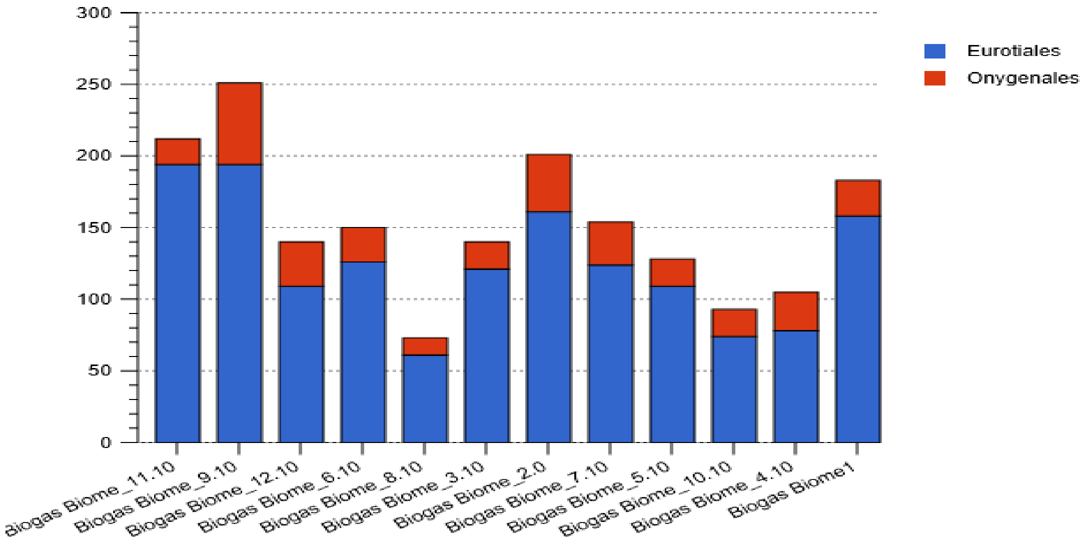

b

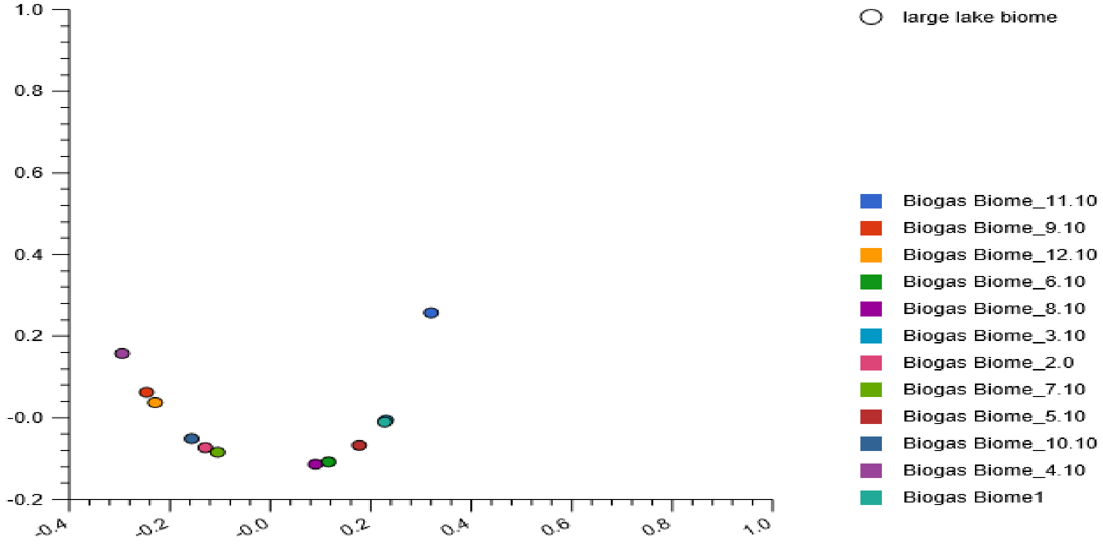

S45 Fig. Stacked barchat (a) showing the two *Eurotiomycete*s orders, the proportion of relative abundances and their PCoA plot (b) based on the Euclidean model. The nucleotide composition of reactor 1 and 3 clustered on the x-axis (positive PCoA 1 and Positive PCoA2); those of reactor 2 and 7, were positioned on the lower left quadrant while the communities of reactor 6 and 8, were found in close proximity on the lower right quadrant of the plot. However, the nucleotide compositions of reactor 11 were singly located on the upper right quadrant of the plot.
