# Supplementary material for: Metagenomics survey unravels diversity of biogas microbiomes with potential to enhance productivity in Kenya
Source: PLoS One. 2021 Jan 4;16(1):e0244755. doi: 10.1371/journal.pone.0244755 (PMC7781671; doi:10.1371/journal.pone.0244755)
Supplement: S46 Fig — Stacked barchat showing four Basidiomycota classes, the relative abundances (a) and their PCoA plot based on the Euclidean model (b). The nucleotide composition of reactor 4 and 9 were found in close proximity, positioned on the upper right quadrant of the plot. The composition of reactor 3 and 11 were also closely located on the upper right quadrant, near the x-axis. The rest of the communities’ nucleotide compositions in other treatments were distributed within the four plot quadrant. (PDF) [file pone.0244755.s047.pdf]

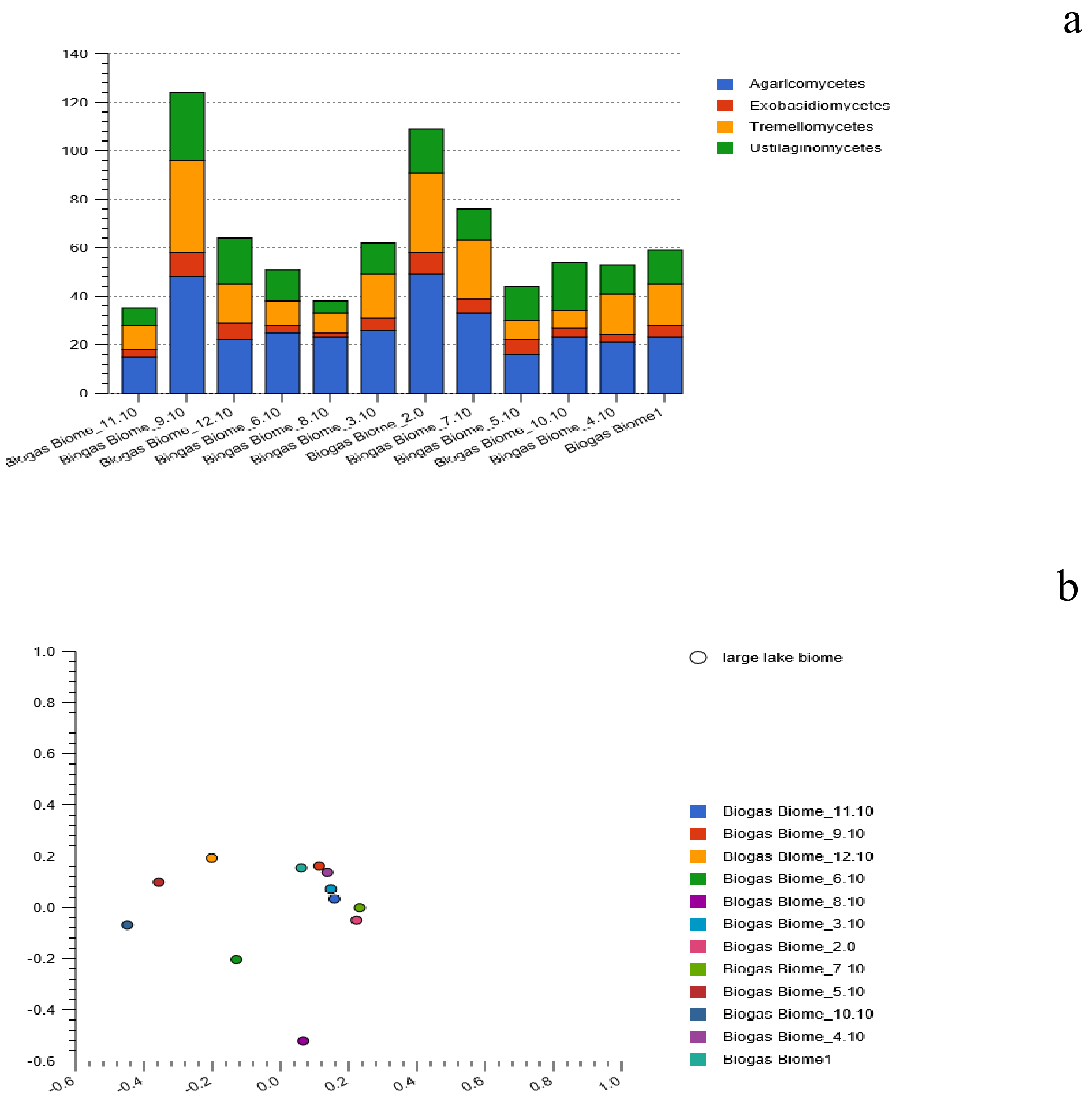

S46 Fig. Stacked barchat (a) showing four *Basidiomycota* classes, the proportion of the relative abundances and their PCoA plot (b) based on the Euclidean model. The nucleotide composition of reactor 4 and 9 were found in close proximity, positioned on the upper right quadrant of the plot. The composition of reactor 3 and 11 were also closely located on the upper right quadrant, near the x-axis. The rest of the communities' nucleotide compositions in other treatments were distributed within the four plot quadrant.
