# Supplementary material for: Metagenomics survey unravels diversity of biogas microbiomes with potential to enhance productivity in Kenya
Source: PLoS One. 2021 Jan 4;16(1):e0244755. doi: 10.1371/journal.pone.0244755 (PMC7781671; doi:10.1371/journal.pone.0244755)
Supplement: S47 Fig — Stacked barchat showing four Basidiomycota orders, relative abundances (a) and their PCoA plot based on the Euclidean model (b). The nucleotide compositions in the twelve treatments were dissimilar and were distributed within the four quadrants. (PDF) [file pone.0244755.s048.pdf]

a

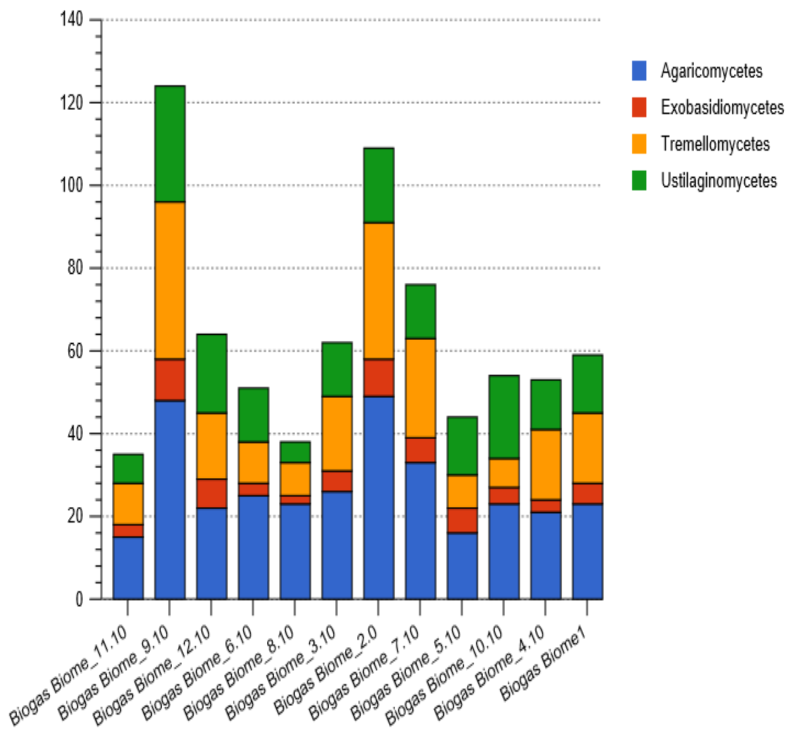

b

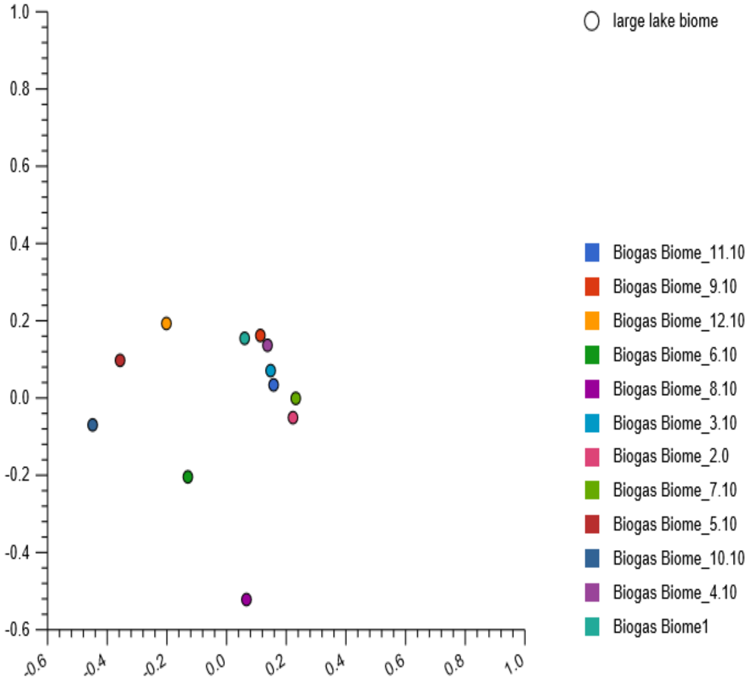

S47 Fig. Stacked barchat (a) showing four *Basidiomycota* orders, relative abundances and their PCoA plot (b) based on the Euclidean model. The nucleotide compositions in the twelve treatments were dissimilar and were distributed within the four quadrants.
