# Supplementary material for: Metagenomics survey unravels diversity of biogas microbiomes with potential to enhance productivity in Kenya
Source: PLoS One. 2021 Jan 4;16(1):e0244755. doi: 10.1371/journal.pone.0244755 (PMC7781671; doi:10.1371/journal.pone.0244755)
Supplement: S48 Fig — Stacked barchart showing the proportion of the Agaricomycetes order, relative abundances (a) and their PCoA plot based on the Euclidean model (b). The nucleotide composition affiliated to Agaricomycetes in reactor 1 and 3 were almost similar, positioned on the upper left quadrant of the plot. Their composition in reactor 7 and 9 were in close proximity, located in the lower left quadrant of the plot. The Agaricomycetes nucleotides in reactor 11 and 12 clustered partially on the lower left quadrant of the plot, those of reactor 5 were singly located in the upper right quadrant of the plot. (PDF) [file pone.0244755.s049.pdf]

a

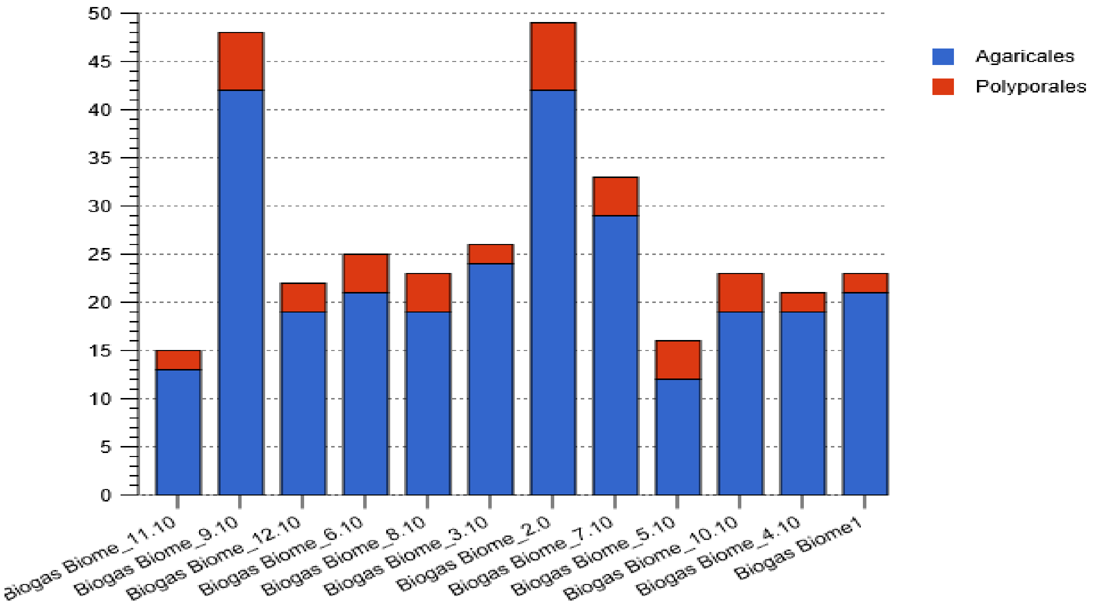

b

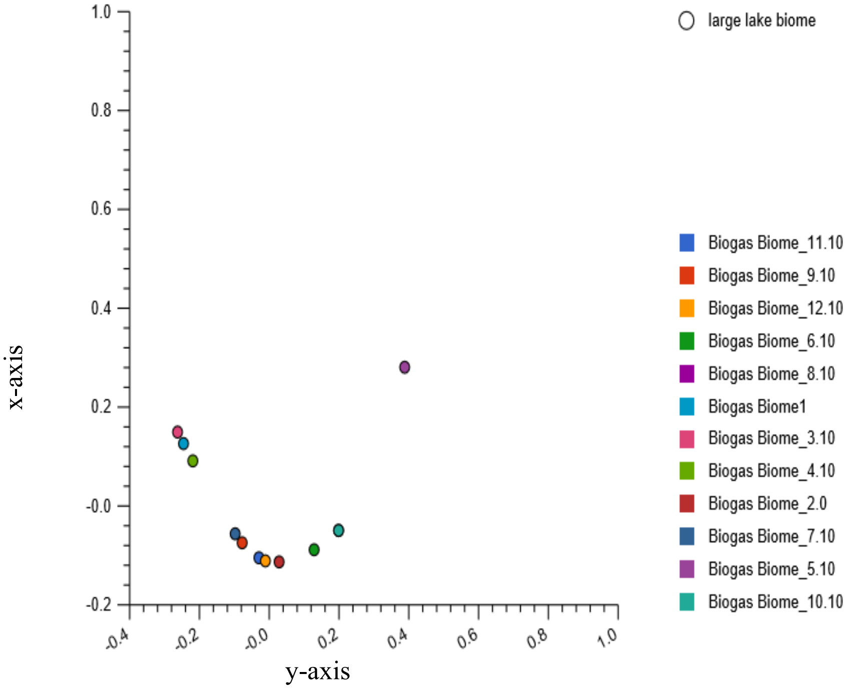

S48 Fig. Stacked barchart (a) showing the proportion of the *Agaricomycetes* relative abundances and their PCoA plot (b) based on the Euclidean model at the order level. The nucleotide composition for *Agaricomycete*s in reactor 1 and 3 were almost similar, located in the upper left quadrant of the Euclidean plot. Their nucleotide composition in reactor 7 and 9 were in close proximity, located in the lower left quadrant of the plot. The *Agaricomycete*s nucleotides in reactor 11 and 12 partially clustered in the lower left quadrant of the plot. However, those detected in reactor 5 were singly located in the in the upper right quadrant of the plot.
