# Supplementary material for: Metagenomics survey unravels diversity of biogas microbiomes with potential to enhance productivity in Kenya
Source: PLoS One. 2021 Jan 4;16(1):e0244755. doi: 10.1371/journal.pone.0244755 (PMC7781671; doi:10.1371/journal.pone.0244755)
Supplement: S49 Fig — This is an indication of special substrates in the two treatments that were absent in other studied treatnents. (PDF) [file pone.0244755.s050.pdf]

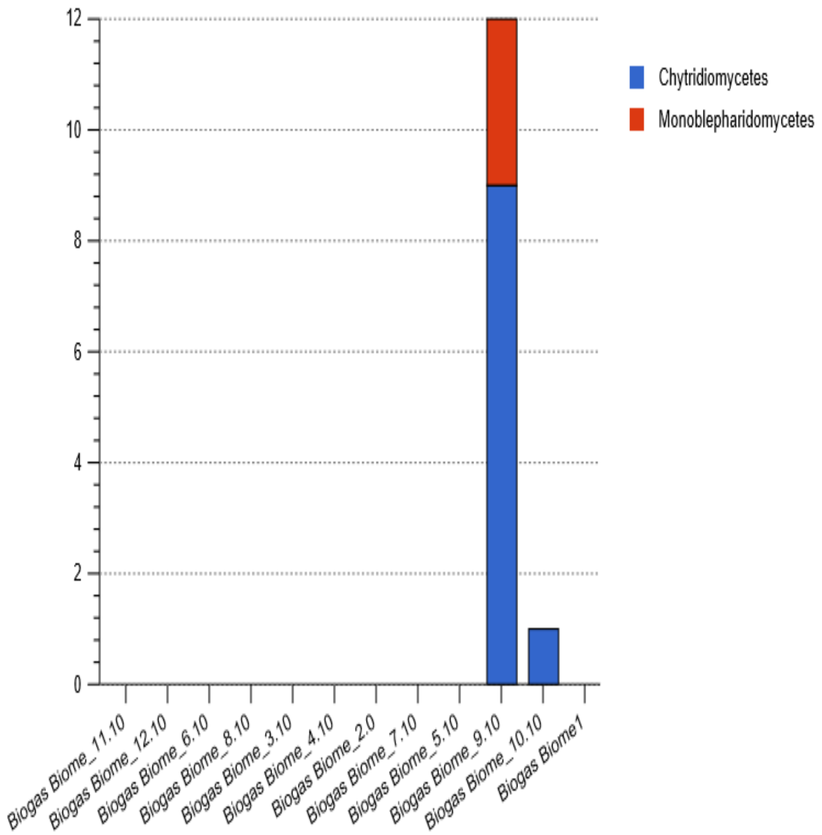


**SFig. 49: Stacked barchat showing the two *Chytridiomycota* classes and their relative abundances in the two treatments.**

This is an indication of special substrates in the two treatments that were absent in other studied treatnents.
