# Supplementary material for: Metagenomics survey unravels diversity of biogas microbiomes with potential to enhance productivity in Kenya
Source: PLoS One. 2021 Jan 4;16(1):e0244755. doi: 10.1371/journal.pone.0244755 (PMC7781671; doi:10.1371/journal.pone.0244755)
Supplement: S51 Fig — The orders are considered rare due to the fact that they were detected in only three treatments. (PDF) [file pone.0244755.s052.pdf]

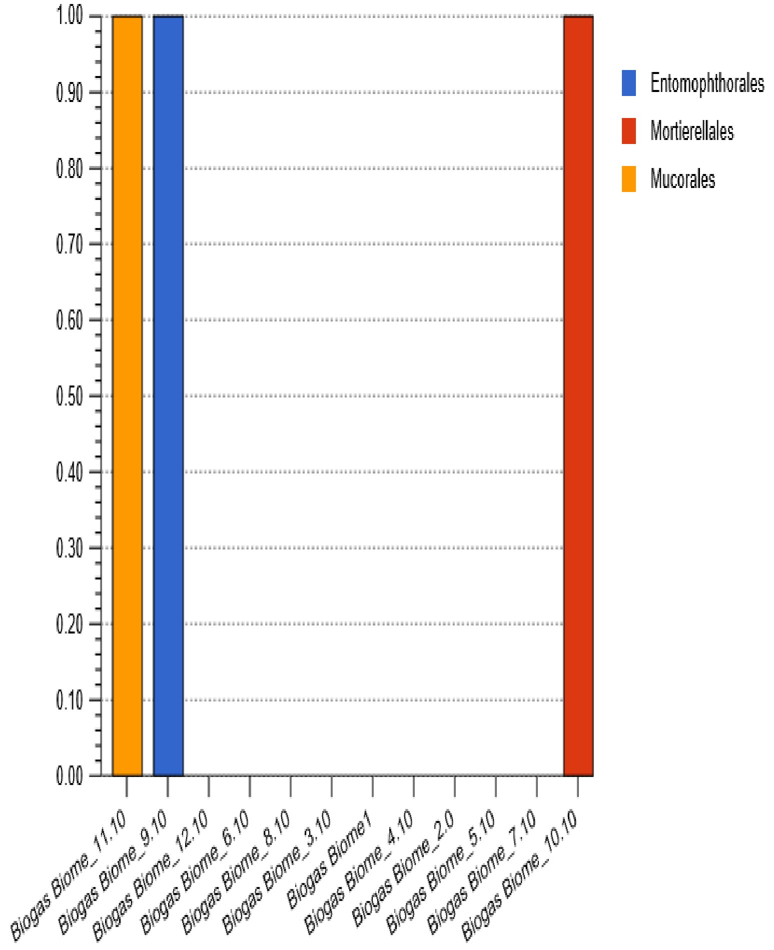

S51 Fig. **Stacked barchat showing the three orders affiliated to unclassified fungal nucleotide reads.** The orders are considered rare due to the fact that they were detected in only three treatments.
