# Supplementary material for: Metagenomics survey unravels diversity of biogas microbiomes with potential to enhance productivity in Kenya
Source: PLoS One. 2021 Jan 4;16(1):e0244755. doi: 10.1371/journal.pone.0244755 (PMC7781671; doi:10.1371/journal.pone.0244755)
Supplement: S52 Fig — The PCoA analysis revealing β-diversity of the twelve treatmenets at three taxa level: a) Domain, b) Phylum, and c) Class level. At the class level, the reads/nucleotide sequences of reactor 2 and 10 were partially similar, located in the lower left quadrant, those of reactor 8 and 11 were located on the upper right quadrant, reactor 4 and 9 compositions on the lower right quadrant, while those of reactor 3 and 6 were located on the lower left quadrant of the plot. (PDF) [file pone.0244755.s053.pdf]

a) Domain

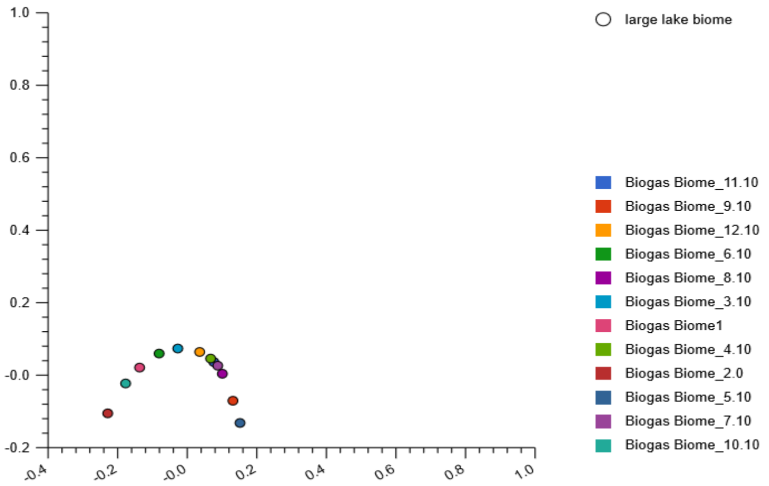

b) Phylum

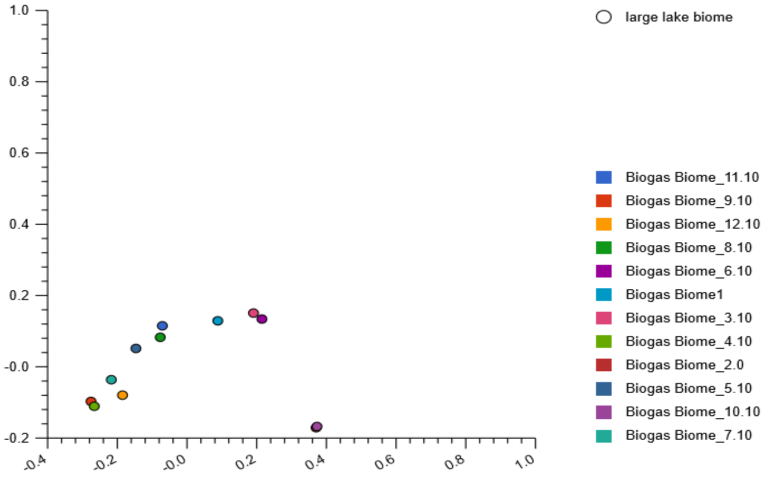

c) Class

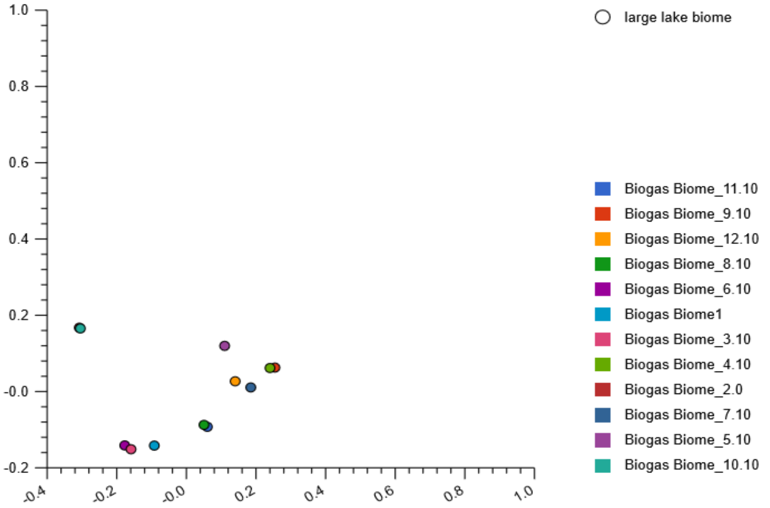

S52 Fig. The PCoA analysis revealing β-diversity of the twelve reactors at the three taxa level: a) Domain, b) Phylum, and c) class level. At the class level, the reads/nucleotide sequences of reactor 2 and 10 were partially similar, located in the lower left quadrant, those of reactor 8 and 11 were located on the upper right quadrant, reactor 4 and 9 compositions on the lower right quadrant, while those of reactor 3 and 6 were located on the lower left quadrant of the plot.
