# Supplementary material for: Metagenomics survey unravels diversity of biogas microbiomes with potential to enhance productivity in Kenya
Source: PLoS One. 2021 Jan 4;16(1):e0244755. doi: 10.1371/journal.pone.0244755 (PMC7781671; doi:10.1371/journal.pone.0244755)
Supplement: Attachment — Submitted filename: Response to Reviewers.pdf [file pone.0244755.s054.pdf]

Samuel Mwangangi Muturi
Department of Biological Sciences,
University of Eldoret.
e-mail: samuelmuturi37@gmail.com

To:
The Academic Editor,
PLOS one Journal

Dear Sir,

<u>REF: RESPONSE TO THE EDITOR AND REVIEWERS COMMENT ON THE MANUSCRIPT NO. PONE-D-20-20281</u>

Thank you for reviewing and accepting our manuscript to be published in PLOS one Journal. To the best of our knowledge, attached below are raised concerns and their respective responses/actions.

A). ACADEMIC EDITOR COMMENTS

1. **Summary of the PLOS ONE's style requirements responses-** including those for file naming

i). PLOS guidelines for title page were followed as recommended.
ii). Authors, and affiliations; abstract and introduction are ok, in that order. PLOS guidelines followed.
iii). "ABSTRACT" revised to "Abstract"
iv). Level 1 headings: Bold type, 18pt font and in sentence case.
v). Level 2 headings: Bold type, 16pt font and in sentence case.
vi). Guidelines for Figure citations and labeling followed as recommended.
vii). Guidelines for File and figure naming were also followed.
viii). The documents is presented in double-space paragraph format.
ix). Continuous line numbers inserted.
x). PLOS Guidelines for formatting tables and table Citations in the text followed.
xi). Guidelines for Reference Citations in the text followed as recommended.
xii). PLOS Guidelines for Supporting Information Citations in the text followed.
xiii). Removed funding, and competing interests information from the acknowledgments section.
xiv). References style revised as per the PLOS guidelines and identifiers added.
xv). Supporting information section, level 1 heading: Bold type font 18 pt.
xvi). Supporting Information Captions formatted as per the PLOS guidelines.
xvii). Guidelines for file Naming for Supporting Information followed.
xviii). PACE used to prepare figures

xix). We chose to present our manuscripts as: i). Results ii). Discussion iii). Conclusion and iv). Materials and methods. As per the PLOS guidelines, the said elements can be presented in any order.
xx). The order: Acknowledgements, supporting materials captions and References, was revised to Acknowledgements, References, Supporting information captions as recommended in the ending sections.
xxi). Figure captions are inserted immediately after the first paragraph in which the figure is cited. Figure files are uploaded separately.
xxii). Tables are inserted immediately after the first paragraph in which they are cited.
xxiii). Supporting information files are uploaded separately.
2. Title - Revised as recommended or stated by the Editor

3. Datasets uploaded in a stable, public repository. The following are the accession no. mgm4776224.3; mgm4776218.3; mgm4776192.3; mgm4776124.3; mgm4775917.3; mgm4775884.3; mgm4775867.3; mgm4775851.3; mgm4775675.3; mgm4775550.3; mgm4775461.3; mgm4775281.3.

4. Authors contribution removed/deleted from the manuscript

I agree with the following statement. Note that most authors including those affiliated to the funding organization, joined the project at advanced level of the project. So they basically reviewed the manuscript. I have accurately revised the author roles in the online submission
Please assist in revising the statement below.

"The funder provided support in the form of salaries and research materials for authors [SMM], but did not have any additional role in the study design, data collection and analysis, decision to publish, or preparation of the manuscript. The specific roles of each author are articulated in the 'author contributions' section."

B). REVIEWER'S COMMENTS
1. **Abstract-** The abstract is too long and should be more concise to the important findings in the present work.
Response: We have revised and shortened the abstract, from 318 words to 295 words.

2. "Furthermore, 39.75% to 46.70% and 53.07% to 60.11% genes were conveyed to known and unknown proteins'- Perhaps to include the examples of known protein in the sentence. **Response:** Our focus is taxonomy of the identified microbes not their encoded proteins. The statistics state the percentages and nature (type of encoded proteins) of the genes and what was used in our taxonomic classifications or identification. We feel this is enough as far as the manuscript is concerned; else, we shall be entering into functionality of the identified microbes, contrary to the aim of the Manuscript.

3. “Hence, only four out of twelve treatments had high β-diversities, which could indicate genetic plasticity due to species evolution”- Is there any solid evidence for this statement?

Response: Yes, the PCoA plot were generated at ($P \leq 0.05$), and present a solid evidence for the above statement. We used nucleotide sequences (genetic composition) to determine β-diversity. High β-diversity is a function of evolution. Evolution leads to genetic versatility that may result to new species and strains. Also included citations; see Randall Hughes et al., 2008; Legendre P. and Caceres M. 2013.

4. Fig. 1 & 2-Proper unit should be indicated in the Y-axis. **Response**: Revised to add more details on the Y-axis of the two figures and other figures.

5. Figure 3- How about sections that are not indicating of any percentage? Any discussion for this?

Response: We’ve expanded the Krona radial space filling display” to capture the comments and to reveal other un-visible percentages see Fig 3a & b.tiff.

6. “…….implying metabolic and genetic plasticity among the identified Clostridia species.”- More justifications are required for this statement.

Response: To the best of our knowledge, the statement is correct. The word is ‘implying’ which create an experimental gap that can be confirmed later. Also (Randall Hughes et al., 2008; Legendre P. and Caceres M. 2013). The statements is not stated as part of our findings but stated as part of our thought/suggestion based on literature.

7. Figure 4- Higher resolution is required.
Response: The resolution of the figure and other figures were improved through the PACE.

8. “The local communities of the other treatments were observed to be significantly dissimilar”- why?
Response: Because all our analysis was conducted at $P \leq 0.05$. On the PCoA plot the “other treatments” were clearly separated at 95% confidence level or threshold.

9. “Generally the reactor treatments comprised high salts concentrations….”- How high is the salt concentration?

Response: Our systems had almost similar salt components and concentrations (unpublished) as those reported by Jha et al., 2013 and Langer et al., 2019.

Furthermore, cow-dung treating reactors contain high amount of salts and ammonia (Usack J. and Angenent L. 2015; Gallert C., Bauer S. and Winter J., 1998; Fang C., Boe K. and Angelidaki I., 2011; Zhang et al., 2019; Rath K. and Rousk J., 2015; De Vrieze et al., 2007).

10. “…low amino acid affinities among their identified species, which would suggest another selection pressure in the AD system”- Is there any evidence on this statement?

Response: Yes based on the literature, low substrates lead to selection pressure, where more competitive species i.e those with high substrates affinities dominates the niche. The phenomenon has been previously reported in methanosarcina and methanosaeta species on acetate substrate [see Goux et al., 2015; Konopka et al., 2015 among others].

11. “Due to this co-operation, we further postulate the existence of a holobiontic mechanism in our treatments….” More evidences are required to support this statement.
Response: We have revised the statement to replace “that is used to regulate” with “which could be regulating”. Also have cited; Bispo PdC. et al., 2017; https://doi.org/10.1371/journal.pone.0188300. The term “Postulate” express our feeling on the synergism among the involved microbes based on our best knowledge concerning the biogas systems.

12. How about the methodology to determine the reactor performance as indicated in Table 3?

Response: The missing statement on the method and material to determine performance included.

Others:

- ✓ Also replaced “Geographic positioning system” with “Global positioning system” in the methodology section. The term is better than the previous one.
- ✓ Revised and inserted more institutions in the Ethics statements section.
- ✓ Revised Bioinformatics and statistics section (see the revised Manuscript).

To the best of my knowledge, we have incorporated all the raised suggestions/comments and the manuscript looks better.

Thank you very much.

Yours Faithfully

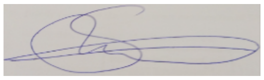

Samuel M. Muturi
Corresponding Author
